# Sex-specific modulation of early life vocalization and cognition by *Fmr1* gene dosage in a mouse model of Fragile X Syndrome

**DOI:** 10.1186/s13293-024-00594-3

**Published:** 2024-02-21

**Authors:** Gabriele Giua, Daniela Iezzi, Alba Caceres-Rodriguez, Benjamin Strauss, Pascale Chavis, Olivier J. Manzoni

**Affiliations:** 1 INMED, INSERM U1249, Marseille, France; 2 Aix-Marseille University, Marseille, France; 3 INSERM U901, Parc Scientifique de Luminy, Marseille cedex 09, BP13 – 13273 France

**Keywords:** Sex, Autism spectrum disorder, Fragile X Syndrome, Ultrasonic vocalizations, Deep learning, Homing behavior

## Abstract

**Background:**

Pup-dam ultrasonic vocalizations (USVs) are essential to cognitive and socio-emotional development. In autism and Fragile X Syndrome (FXS), disruptions in pup-dam USV communication hint at a possible connection between abnormal early developmental USV communication and the later emergence of communication and social deficits.

**Methods:**

Here, we gathered USVs from PND 10 FXS pups during a short period of separation from their mothers, encompassing animals of all possible genotypes and both sexes (i.e., *Fmr1-/y* vs. *Fmr1+/y* males and *Fmr1+/+*, *+/-*, and *-/-* females). This allowed comparing the influence of sex and gene dosage on pups’ communication capabilities. Leveraging DeepSqueak and analyzing vocal patterns, intricate vocal behaviors such as call structure, duration, frequency modulation, and temporal patterns were examined. Furthermore, homing behavior was assessed as a sensitive indicator of early cognitive development and social discrimination. This behavior relies on the use of olfactory and thermal cues to navigate and search for the maternal or nest odor in the surrounding space.

**Results:**

The results show that FMRP-deficient pups of both sexes display an increased inclination to vocalize when separated from their mothers, and this behavior is accompanied by significant sex-specific changes in the main features of their USVs as well as in body weight. Analysis of the vocal repertoire and syntactic usage revealed that *Fmr1* gene silencing primarily alters the USVs’ qualitative composition in males. Moreover, sex-specific effects of *Fmr1* silencing on locomotor activity and homing behavior were observed. FMRP deficiency in females increased activity, reduced nest-reaching time, and extended nest time. In males, it prolonged nest-reaching time and reduced nest time without affecting locomotion.

**Conclusions:**

These findings highlight the interplay between *Fmr1* gene dosage and sex in influencing communicative and cognitive skills during infancy.

**Supplementary Information:**

The online version contains supplementary material available at 10.1186/s13293-024-00594-3.

## Abstract (plain language summary)

In this study, we investigated ultrasonic vocalizations (USVs) and homing behavior in a mouse model of Fragile X Syndrome (FXS), a leading genetic cause of autism spectrum disorder (ASD) caused by a mutation of the X-chromosome linked *Fmr1* gene. Disruptions in pup-dam USV communication and cognitive skills may be linked to the later emergence of communication and social deficits in ASD. USVs were collected from 10-day-old FXS pups of all possible genotypes and both sexes during a short period of separation from their mothers. We utilized DeepSqueak, an advanced deep learning system, to examine vocal patterns and intricate vocal behaviors, including call structure, duration, frequency modulation, and their temporal patterns. Homing, a sensitive indicator of early cognitive development and social discrimination was assessed at P13. The results showed that FXS pups of both sexes displayed an increased inclination to vocalize when separated from their mothers. Examination of the vocal repertoire and its syntactic usage revealed that the silencing of the *Fmr1* gene primarily alters the qualitative composition of ultrasonic communication in males. The sex-specific changes observed in USVs were accompanied by modifications in body weight. Regarding homing behavior, the deficiency of FMRP led to opposite deficits in activity, time to reach the nest, and nesting time depending on sex. Taken together, these findings highlight the interplay between *Fmr1* gene dosage and sex in shaping communication and cognition during infancy.

**Supplementary Information:**

The online version contains supplementary material available at 10.1186/s13293-024-00594-3.

## Highlights

We investigated early life communicative and cognitive abilities in a mouse model of Fragile X Syndrome (FXS), a prominent genetic cause of autism spectrum disorder resulting from a mutation in the *Fmr1* gene on the X-chromosome.

Ultrasonic vocalizations (USVs) and homing behavior were collected respectively from 10- and 13-day-old FXS pups, encompassing all possible genotypes and both sexes, during a brief separation from their mothers.

Both male and female FXS pups exhibited an increased inclination to vocalize when separated from their mothers, along with significant sex-specific alterations in the primary characteristics of their USVs, homing behavior, and body weight. Silencing of the *Fmr1* gene primarily influenced the qualitative composition of ultrasonic communication in males.

In homing behavior, FMRP’s deficiency led to contrary deficits in activity, time to reach the nest, and nesting time depending on sex.

The interaction between *Fmr1* gene dosage and sex impacted on communication and cognition during early infancy.

**Supplementary Information:**

The online version contains supplementary material available at 10.1186/s13293-024-00594-3.

## Introduction

Ultrasonic vocalizations (USVs), are essential to mouse communication and their social behavioral [1], notably in the context of conditions marked by compromised social interaction and communication, such as neurodevelopmental disorders (NDDs) and autism spectrum disorders (ASDs) [1–6]. In most mouse strains, the developmental trajectory of USVs, particularly within the 30 to 110 kHz range [7], remains consistent. The frequency of USV calls usually escalates during the initial 5–6 days following birth, reaching a peak around the sixth or seventh postnatal day (PND). Call rates then begin to diminish and typically cease by the end of the second postnatal week. The precise timing of these transitions could be strain-dependent, with C57BL/6 mice, for instance, typically displaying the highest USV rate around PND 3 [8, 9]. This form of communication is mostly observed during isolation-induced USVs in pups, interaction-induced USVs in both young and adult mice, and courtship-induced USVs in adult mice [5].

First described as “whistles of loneliness” due to their role in provoking maternal care and fostering communication between mother and offspring [10], USVs are a vital communication mechanism for mouse pups in their early weeks of life, and their frequency tends to increase when the pups are isolated from their nest, mother, and siblings [5].

Given its prevalence (1.4 per 10,000 males and 0.9 per 10,000 females in the total population [11]), Fragile X Syndrome (FXS) is the foremost inherited cause of intellectual disability (ID) and the most common syndrome linked with ASD [11, 12]. FXS patients often present a broad spectrum of physical, neurological, social, behavioral, and cognitive anomalies [13]. A salient feature of FXS involves deficits in communication, where delays in speech and language development are common [14]. Children diagnosed with FXS frequently exhibit speech patterns marked by compulsion, repetition, and perseverance [15]. Expressive language delays are a common observation in both male and female FXS patients, with the severity of effects typically being less in females owing to the X-linked nature of the disorder [16, 17].

Prior investigations into the communication abilities of mouse models with FXS produced varied outcomes, which seem to be influenced by factors such as the mouse strain, experimental protocol, and the age of the mice [18–26]. Here we exploited DeepSqueak [27] to evaluate USVs across all genotypes and sexes in the widely used mouse model for FXS (*Fmr1*-KO2 [28]).

In mouse pups, homing behavior is a sensitive indicator of early cognitive development and social discrimination. It provides insights into their ability to navigate and recognize their nest, reflecting their spatial learning and memory capabilities [29–31]. Thus, understanding alterations in homing behavior can contribute to our understanding of cognitive impairments and social communication disorders associated with conditions like FXS and ASD.

The results revealed that the considerable quantitative and qualitative impact of *Fmr1* deficits on early life ultrasonic vocalization, cognitive abilities and motor skills is highly dependent on both sex and gene dosage.

## Results

### *Fmr1* gene silencing results in sex-dependent changes in body weight

The absence of the *Fmr1* gene leads to distinct alterations in body weight based on sex. At PND 10 and PND 13, when FMRP was absent, male mice exhibited reduced weight, while female mice displayed increased weight compared to their respective control groups (Fig. 2; Suppl. Table 1). Additionally, when examining the control animals (those in which FMRP was not manipulated), female mice weighed less than their male counterparts (as illustrated in Fig. 2; Suppl. Table 1) at PND 10 but not PND 13. This result indicates role for FMRP in regulating body weight in a sex-dependent manner.

**Fig. 1:**
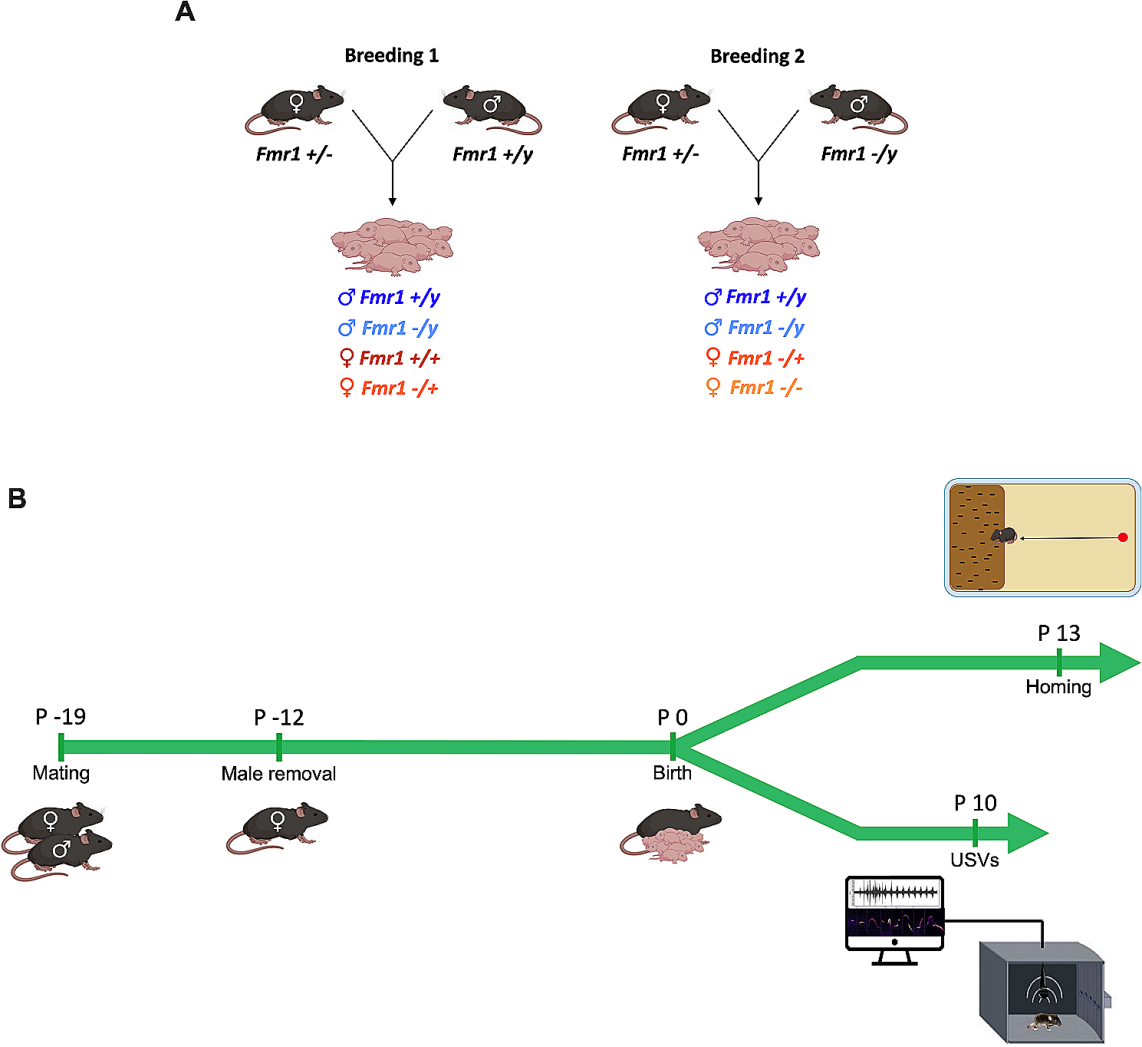
Schematic illustration of the experimental design. In order to generate litters encompassing all potential genotypes, breeding involved crossing either female *Fmr1*+/- with male *Fmr1* +/y or female *Fmr1+/-* with male *Fmr1* -/y. Mice of all genotypes were divided into two cohorts: one underwent USV recording at PND 10, while the other underwent a homing behavior test at PND 13

**Fig. 2:**
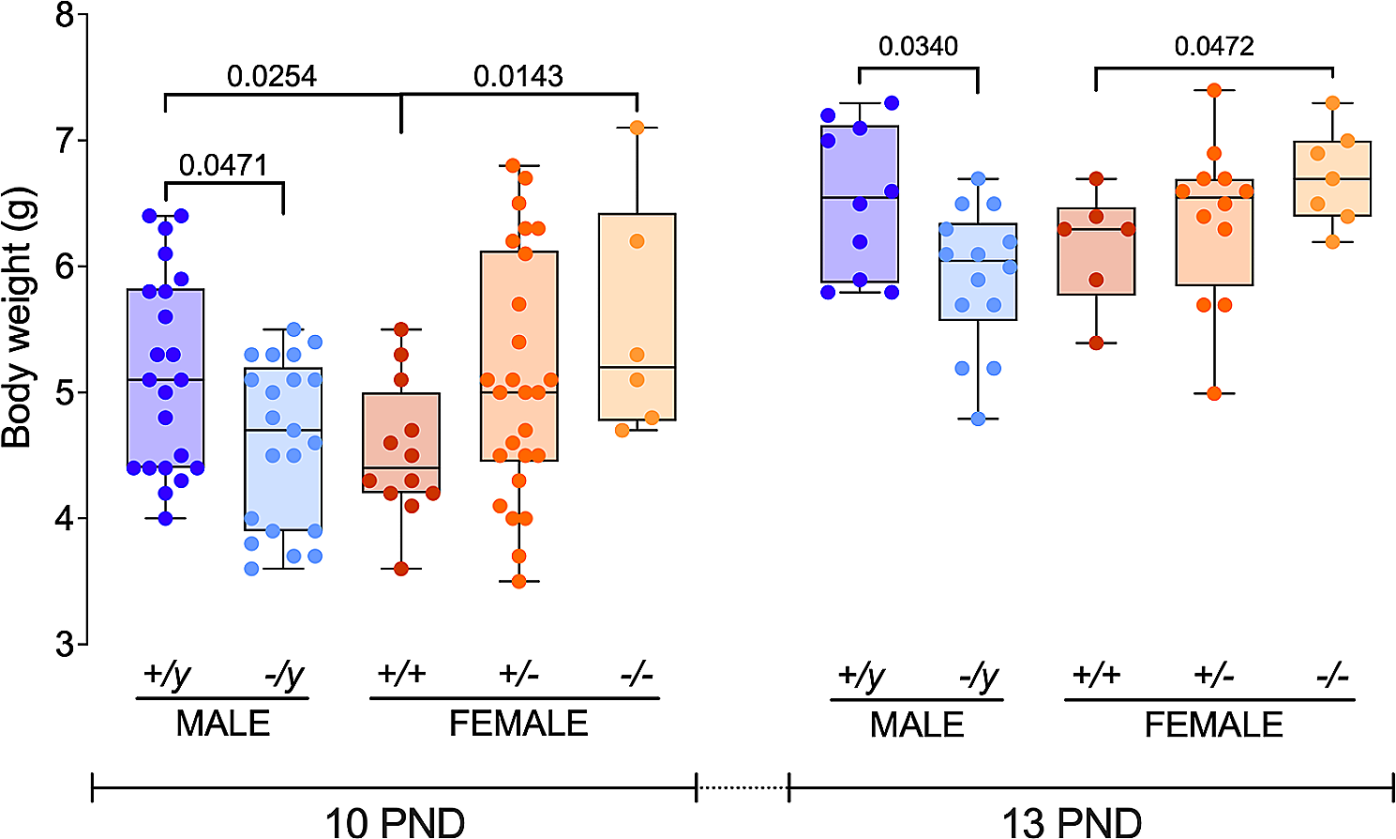
FMRP and sex influence body weight. On PND 10 and PND 13, the absence of FMRP resulted in decreased body weight for male mice (*-/y*) and increased weight for females (*-/-*) when compared to their respective control groups (*+/y* and *+/+*). Among the control groups, male mice (*+/y*) are typically heavier than female mice (*+/+*) at PND 10 but not PND 13. Single dots represent individual mice. The box plots present the data ranging from minimum to maximum values, with median and interquartile range (25–75 percentile) shown. The Mann-Whitney U test was applied for statistical analysis. *p*-values less than 0.05, indicating statistical significance, are marked on the graphs, while complete statistics can be found in Suppl. Table 1. Sample size 10 PND: *+/y* males *N* = 22, *-/y* males *N* = 21, *+/+* females *N* = 12, *+/-* females *N* = 26 and *-/-* females *N* = 6. Sample size 13 PND: *+/y* males *N* = 10, *-/y* males *N* = 14, *+/+* females *N* = 6, *+/-* females *N* = 12 and *-/-* females *N* = 7

### Elevated vocalization responses in FMRP-deficient pups during maternal separation

**Fig. 3:**
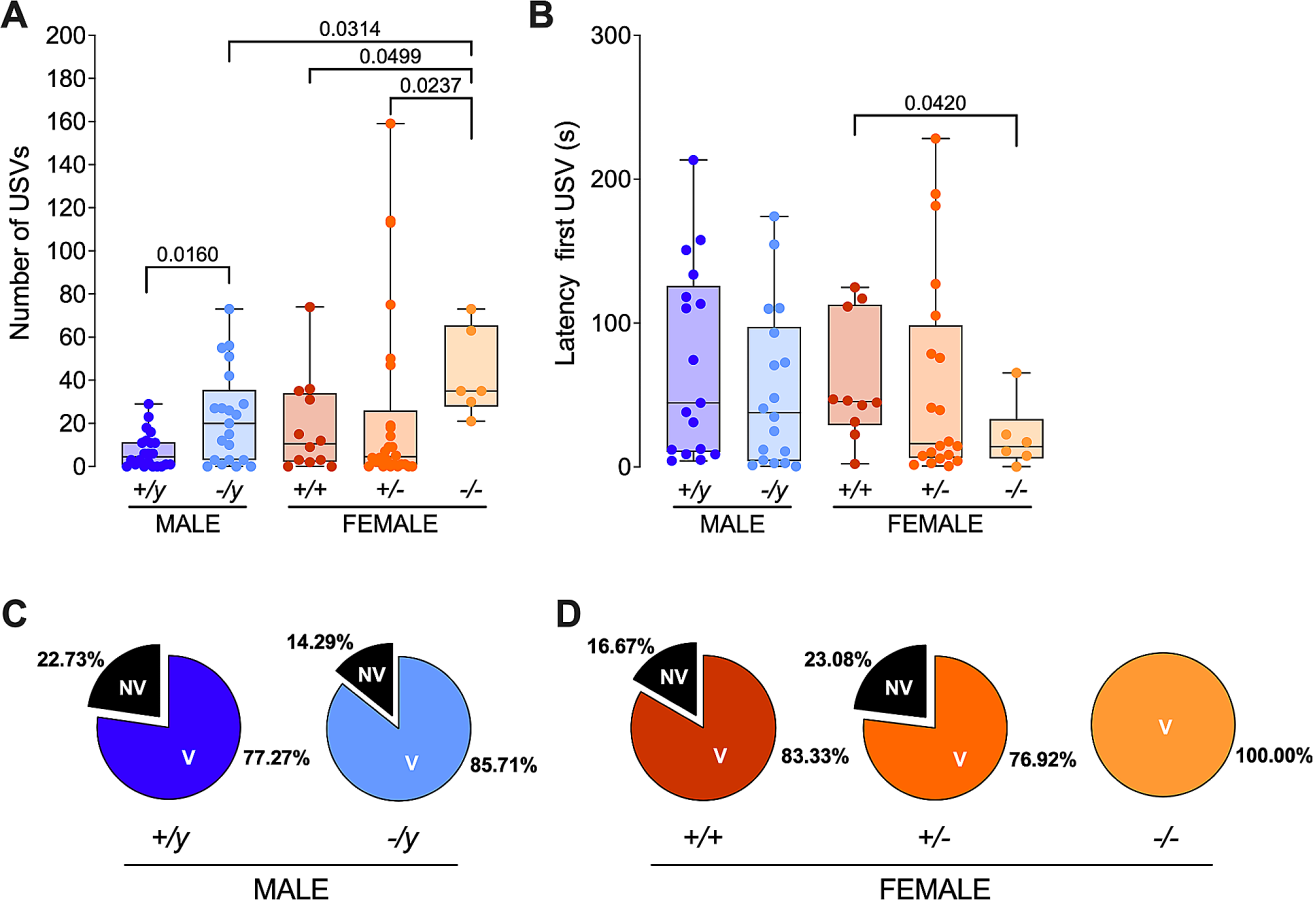
Sex-specific differences in vocalizations and vocalization latency in FXS Pups. (**A**) FXS pups of both sexes display a higher number of vocalizations compared to their control counterparts. (**B**) Only females show a shorter vocalization latency in the absence of FMRP. (**A, B**) Data are presented as min. to max. box plots with median and 25–75 percentile. Single dots represent individual mice. Mann-Whitney U tests were conducted, and *p*-values < 0.05 are indicated in the graphs. Full statistical details can be found in Suppl. Table 2. (**C, D**) Pie graphs illustrate the percentages of vocalizing (V) and non-vocalizing (NV) male (**C**) and female (**D**) pups. The percentages were calculated by dividing the number of vocalizers or non-vocalizers by the total number of animals tested in each group. Sample sizes: (**A, C, D**) *+/y* males *N* = 22, *-/y* males *N* = 21, *+/+* females *N* = 12, *+/-* females *N* = 26, *-/-* females *N* = 6. (**B**) *+/y* males *N* = 17, *-/y* males *N* = 18, *+/+* females *N* = 10, *+/-* females *N* = 20 and *-/-* females *N* = 6

When separated from their mothers, both male and female pups lacking FMRP exhibited a heightened frequency of vocalizations, contrasting with the control group. Notably, this change in vocalization frequency was not observed in partially deficient (*+/-*) females (see Fig. 3A; Suppl. Table 2). Furthermore, a complete absence of FMRP resulted in quicker vocal responses in females, whereas in males, the response time remained comparable to the control group (Fig. 3B; Suppl. Table 2). Examining the proportion of vocalizing (V) and non-vocalizing (NV) pups across the groups revealed that both male and female FMRP-deficient pups (*-/y* and *-/-*, respectively) had a higher percentage of vocalizers compared to their respective control groups (*+/y* and *+/+*, respectively) (see Fig. 3C; Suppl. Table 2). This straightforward analysis highlights that the absence of FMRP increases the likelihood of vocalization in both sexes during maternal separation.

### Sex-specific impact of FMRP deficiency on fundamental USV Characteristics

**Fig. 4:**
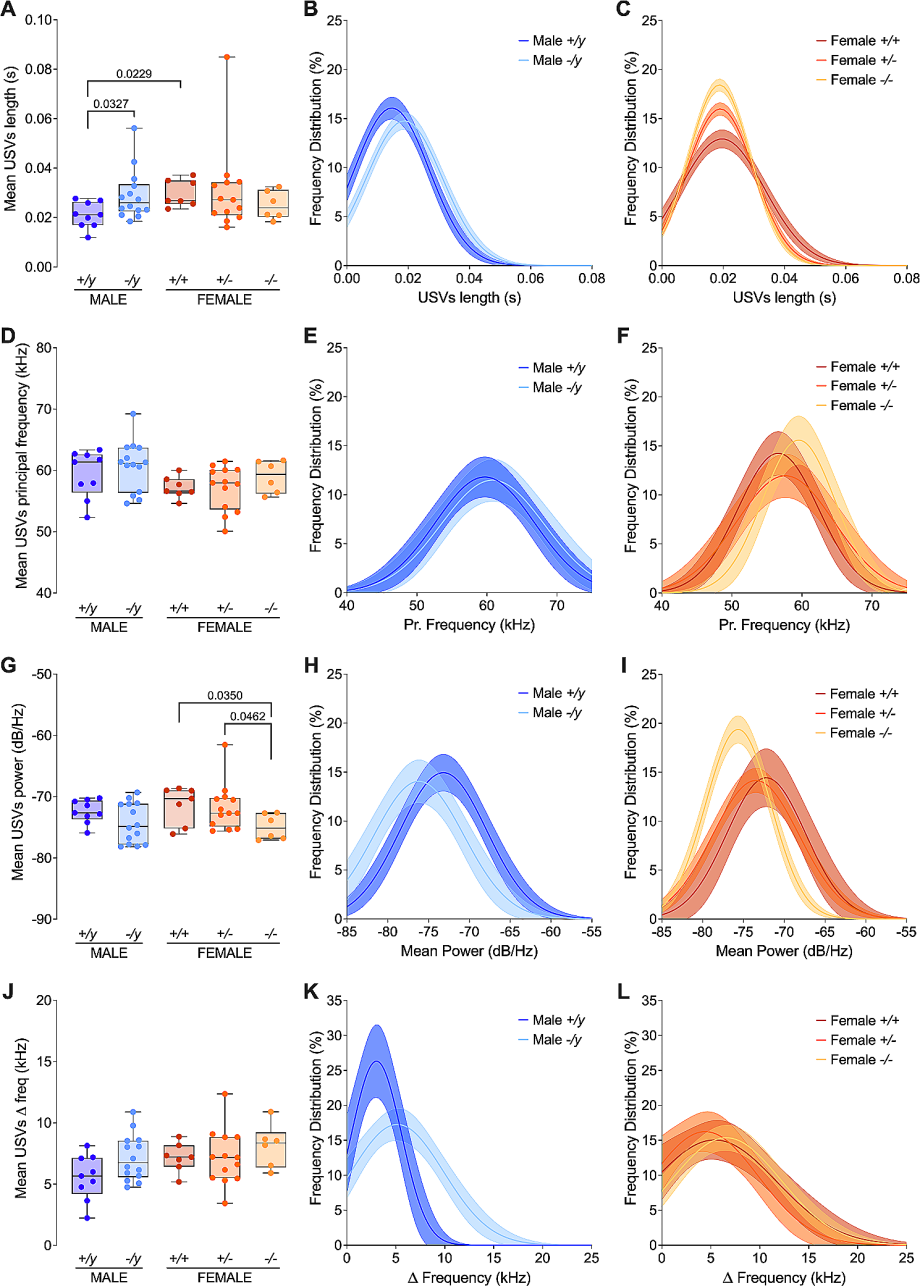
Sex-dependent alteration of core features in USVs of FMRP-deficient mice. (**A**) FMRP deficiency specifically leads to longer mean length of vocalizations in males. (**B, C**) Frequency distribution (%) of USV length shows opposite impacts in male (**B**) and female (**C**) pups. (**D-F**) The principal frequency of vocalizations remains similar across the groups. (**G**) In the absence of FMRP, only females exhibit a statistically more negative mean power in their USVs. (**H, I**) Frequency distribution analysis reveals that both sexes show a greater utilization of USVs with more negative power in FMRP-deficient pups. (**J-L**) The mean change in frequency of USVs does not appear to be affected by the FXS genotype in either sex (**J**), but frequency distribution analysis indicates wider delta use in the absence of FMRP in males (**K**) but not in females (**L**). (**A, D, G, J**) Single dots represent individual mice. Data are presented as min. to max. box plots with median and 25–75 percentile. Mann-Whitney U tests were performed, and *p*-values < 0.05 are indicated in the graphs. Full statistical details can be found in Suppl. Table 3. (**B, C, E, F, H, I, K, L**) Data are represented as a Gaussian curve fit (± CI) of the frequency distribution (%). Sample sizes: (**A–L**) *+/y* males *N* = 9, *-/y* males *N* = 14, *+/+* females *N* = 7, *+/-* females *N* = 13 and *-/-* females *N* = 6

Next, the four fundamental characteristics of USVs: length, primary frequency, power, and frequency range were investigated. Female control mice produced longer USVs compared to their male counterparts (Fig. 4A; Suppl. Figure 1A; Suppl. Table 3). Notably, when FMRP was absent, male mice (*-/y*) generated longer USVs than their normal counterparts (*+/y*). This effect, however, was male-specific, as FMRP deficiency did not yield longer USVs in female mice, regardless of being partially or totally deficient (Fig. 4A; Suppl. Table 3). Frequency distribution analysis supported these observations, indicating increased use of longer USVs in FMRP-deficient male mice compared to controls (Fig. 4B). Conversely, female mice displayed a contrasting trend, with control (*+/+*) females using longer USVs more frequently than partially or fully deficient females (Fig. 4C), despite similar average lengths across these female groups (Fig. 3A; Suppl. Table 3). Analysis of the primary frequency of USVs across all groups revealed no significant variations. Although average frequencies were comparable (Fig. 4D; Suppl. Table 3), control females tended to use lower frequencies more frequently than males (Suppl. Figure 1C). This gender difference was less pronounced in FMRP-deficient mice (Suppl. Figure 1D). Comparing the mean power of USVs, males showed little change regardless of FMRP status, whereas FMRP-deficient females shifted towards more negative powers (Fig. 4G; Suppl. Table 3). Further analysis indicated that both FMRP-deficient males and females used USVs with more negative power more often than their respective controls (Fig. 4H, I). Lastly, the average frequency range used in vocalizations did not show any notable variations based on sex or genotype (Fig. 4J; Suppl. Table 3). Nonetheless, frequency distribution analysis suggested a wider range in FMRP-deficient males than in controls (Fig. 4K), with no such difference in females (Fig. 4L). Typically, females use larger frequency ranges more often than males (Suppl. Figure 1G), but this difference disappeared in the absence of FMRP (Suppl. Figure 1H). In conclusion, FMRP deficiency impacted the frequency of vocalizations, and led to sex-specific alterations in the properties of ultrasonic communication.

### Sex differences in the vocal repertoire of FXS mouse

**Fig. 5:**
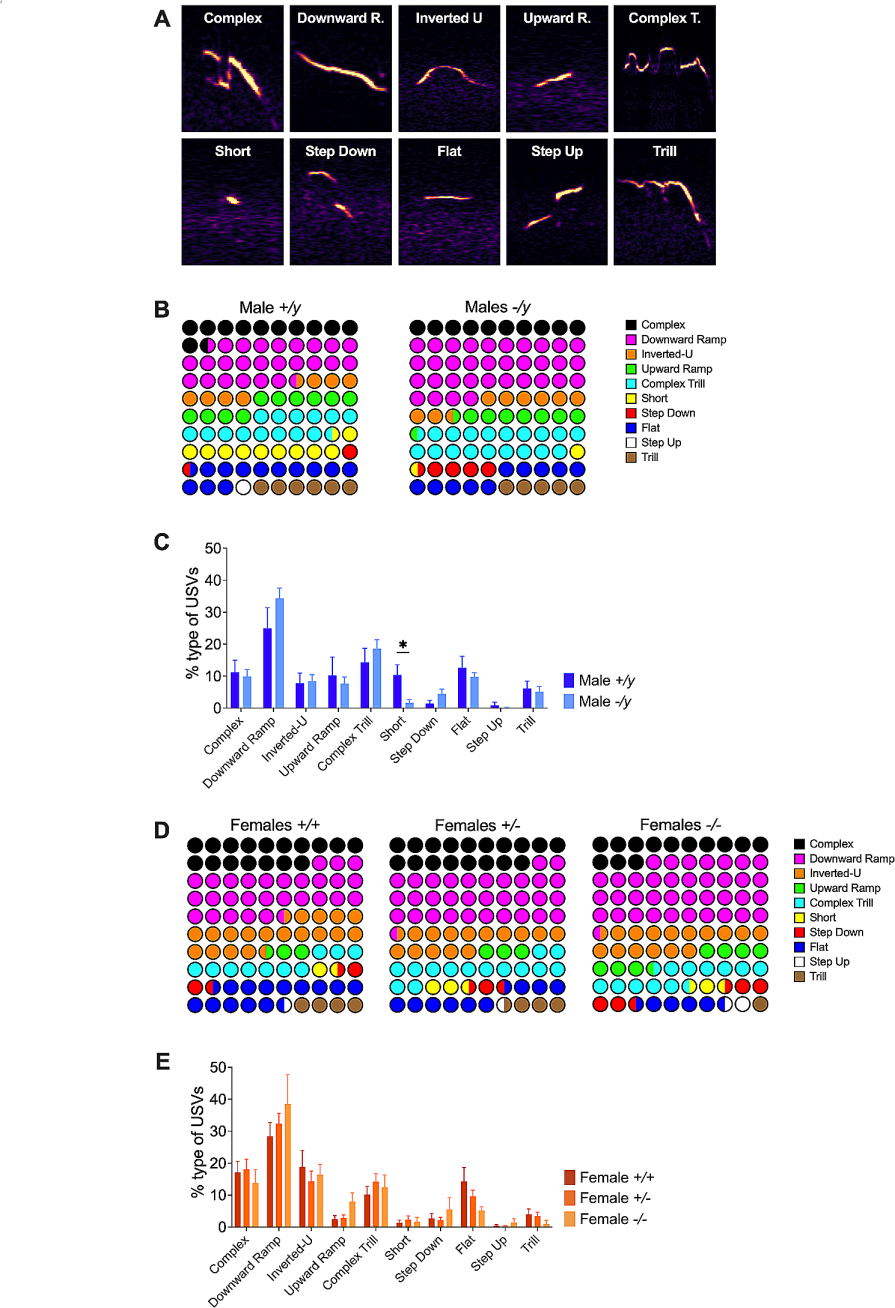
Vocal repertoire of FXS pups. (**A**) Representative USVs calls classified into ten distinct categories based on a supervised-call classification neural network. (**B, C**) In the absence of FMRP, male mice exhibit a limited use of short calls in their vocal repertoire. (**D, E**) The vocal repertoire of female pups remains unaffected by the absence of FMRP. (**B, D**) Data are represented as a percentage utilization of each category of USVs for each group. (**C, E**) Data are shown as a bar graph (mean ± SEM) indicating the percentage utilization of each type of USV category for each group. Significance: * *p*-values < 0.05, full statistical details can be found in Suppl. Table 6. Sample sizes: (**B-E**) *+/y* males *N* = 9, *-/y* males *N* = 14, *+/+* females *N* = 7, *+/-* females *N* = 13 and *-/-* females *N* = 6

Leveraging the call classification capabilities of DeepSqueak [27], 10 unique types of USVs were identified within our dataset (Fig. 5A). Examination of each group vocal repertoire showed differential use of these different USVs during maternal separation (Fig. 5B, D; Suppl. Tables 4, 5). Statistical comparison of these vocal profiles across various genotypes (Fig. 5C, E; Suppl. Table 6), showed that in male mice lacking FMRP, there was a substantial decrease in the use of ‘Short’ vocalizations (Fig. 5C; Suppl. Table 6). In contrast, female vocal repertoire remained unaltered in the absence of FMRP (Fig. 5E; Suppl. Table 6). When analyzing vocalizations based on sex, male and female control mice showed similar vocal profiles. In contrast, in FMRP-deficient mice, males used ‘Inverted-U’ vocalizations less often and ‘Flat’ vocalizations more frequently compared to their female counterparts (Suppl. Table 6). Despite the absence of FMRP having a significant effect, all groups still utilized the same ten vocalizations. Thus, the lack of FMRP predominantly affected the vocal repertoire of male mice.

### Differential modification of vocal transitions by FMRP deficiency in male and female mice

**Fig. 6:**
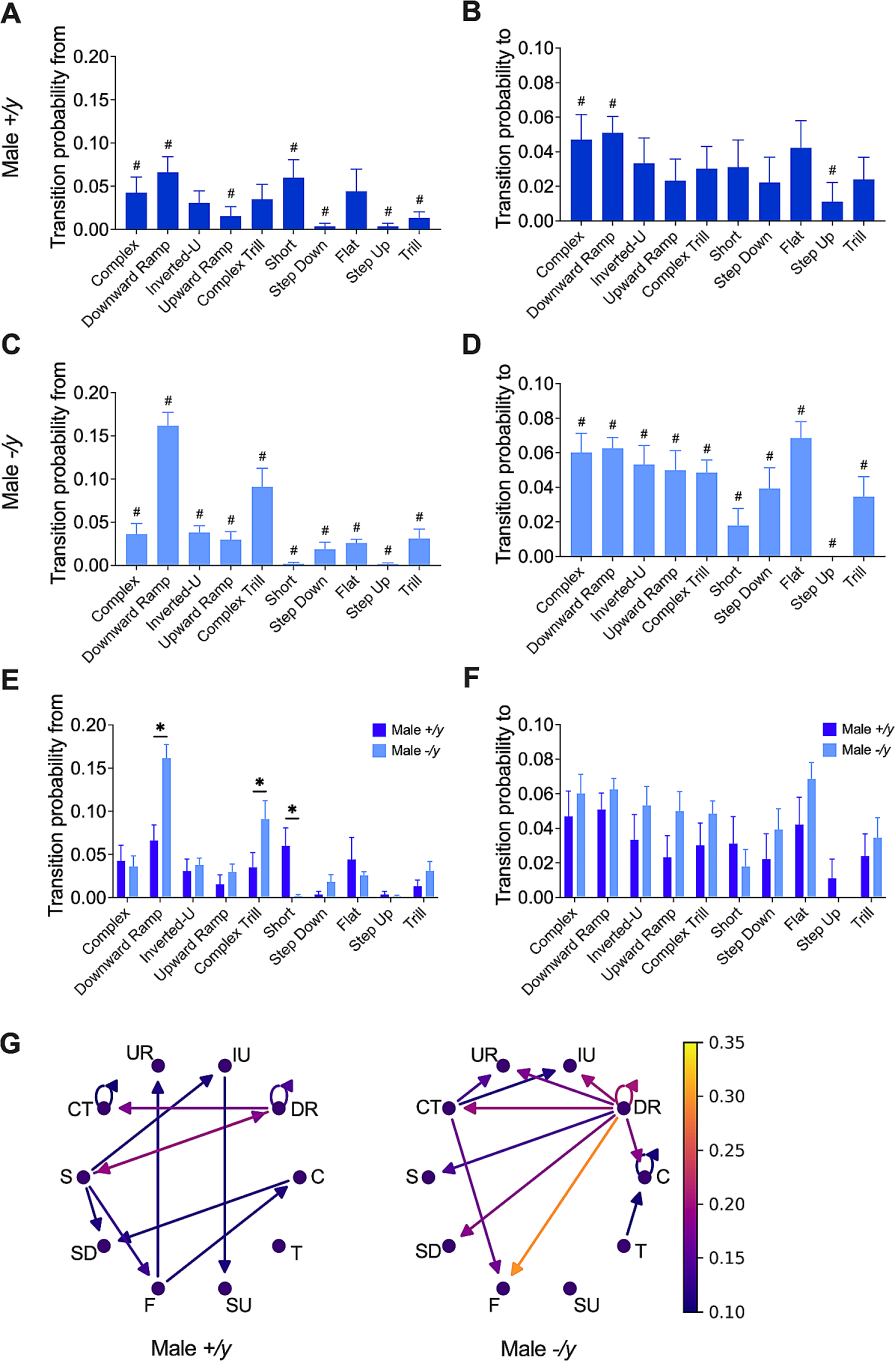
Syntactic transition probability in male FXS pups. **(A – D)** The probability of transitions ‘from’ and ‘to’ a specific USV class varies among different vocalization classes in *+/y* (**A, B**) and *-/y* (**C, D**) males. (**E, F**) Transition probabilities differ ‘from’ a specific USV class (**E**), but not ‘to’ a specific class (**F**). (**G**) Qualitative illustration of transition probability profiles for males of various genotypes. (**A-F**) Data are shown as a bar graph (mean ± SEM). The *p*-values < 0.05 are indicated with an asterisk (*) and the full statistics can be found in Suppl. Tables 9 and 12. The hashtag (#) refers to statistics presented in Suppl. Tables 7 and 10. Statistical analysis was done using Mann-Whitney U tests. (**G**) Arrow diagrams. Arrows indicate transition directions, with brighter colors signifying higher transition probabilities. C = Complex, DR = Downward ramp, IU = Inverted-U, UR = Upward ramp, CT = Complex trill, S = Short, SD = Step Down, F = Flat, SU = Step up, and T = Trill. Sample sizes: (**A-G**) *+/y* males *N* = 9 and *-/y* males *N* = 14

**Fig. 7:**
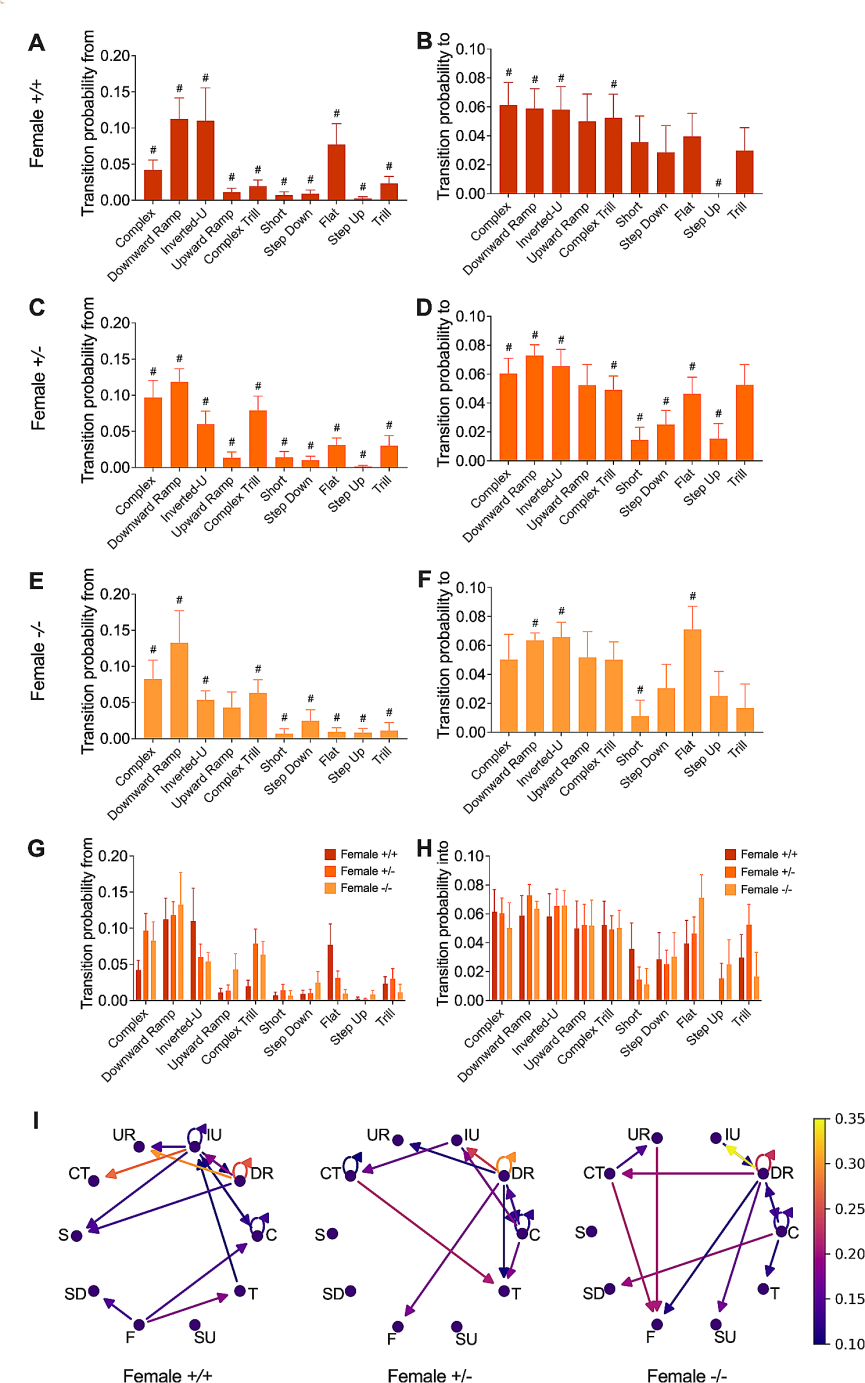
Syntactic transition probability in female FXS pups. (**A–F**) The probability of transitions ‘from’ and ‘to’ a specific USV class varies among different vocalization classes in *+/+* (**A, B**), *+/-* (**C, D**) and *-/-* females (**E, F**). (**G, H**) These profiles are similar among genotypes in the probability of transition “from” (**G**) and “to” (**H**) a specific class of USVs. (**I**) Qualitative illustration of transition probability profiles for females of various genotypes. (**A–H**) Data are shown as a bar graph (mean ± SEM). (**A-F**) The hashtag (#) refers to statistics presented in Suppl. Tables 8 and 11. (**G, H**) Full statistics can be found in Suppl. Tables 9 and 12. (**A-H**) Statistical analysis was done using Mann-Whitney U tests. (**I**) Arrow diagrams. Arrows indicate transition directions, with brighter colors signifying higher transition probabilities. C = Complex, DR = Downward ramp, IU = Inverted-U, UR = Upward ramp, CT = Complex trill, S = Short, SD = Step Down, F = Flat, SU = Step up, and T = Trill. Sample size: (**A-I**) *+/+* females *N* = 7, *+/-* females *N* = 13 and *-/-* females *N* = 6

We next examined the likelihood of transitions between different types of vocalizations within the syntax patterns of the various test groups (Figs. 6 and 7; Suppl. Figure 2; Suppl. Tables 7–12). Detailed analysis of the transition probabilities between each type of USV within each group, uncovered distinct patterns of vocalization use within their syntax (Figs. 6A-D and 7A-F; Suppl. Tables 7, 8, 10, 11). Comparing genotypes statistically revealed that FMRP deficiency altered the likelihood of transitioning from ‘Downward Ramp,’ ‘Complex Trill,’ and ‘Short’ USVs in male pups (Fig. 6E; Suppl. Table 9). Interestingly, the probability of transitioning to different types of USVs remained unchanged between FMRP-normal and deficient males (Fig. 6F; Suppl. Table 9). On the other hand, in female pups, FMRP deficiency did not appear to significantly affect the transition probabilities from and to various USVs (Fig. 6G, H; Suppl. Table 9). In summary, qualitative arrow diagram and heatmap analysis unveiled the intricate web of communication, emphasizing a more profound influence of FMRP on the syntax of male mice (Fig. 6G; Suppl. Figure 2A) compared to females (Fig. 7I; Suppl. Figure 2B).

### Sex differences in the homing behavior of FXS mouse

**Fig. 8:**
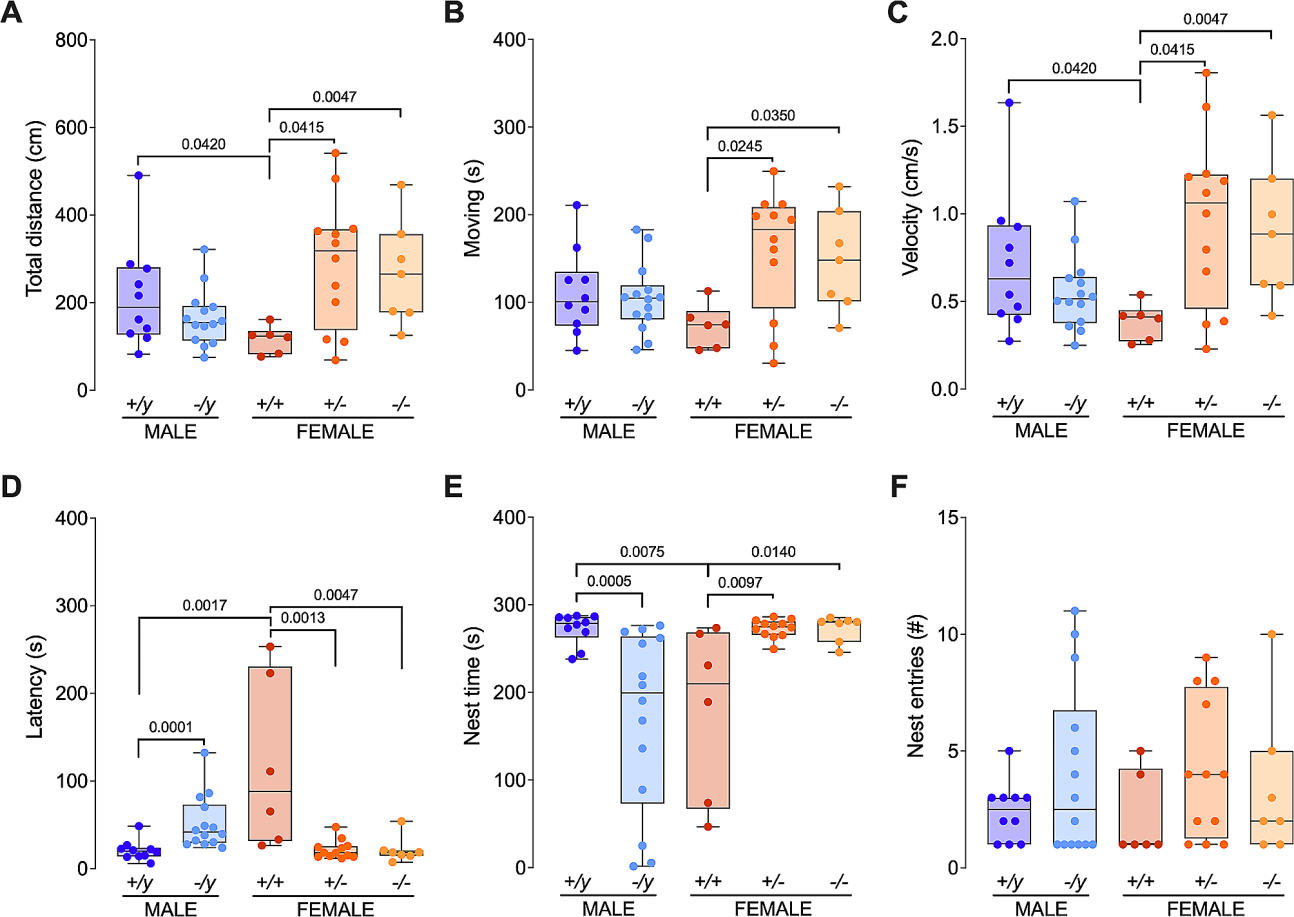
Sex-specific modification of homing behavior in FXS mice. (**A**-**C**) FMRP deficiency has no effect on locomotor activity in male mice while in females it leads to an increase in covered distance (**A**), time moving (**B**) and average velocity (**C**). Among the control groups, males (+/y) move more (**A**) and with greater average velocity (**C**) than females (+/+). (**D-F**) homing behavior in FXS mice displayed distinct sex-related effects. Fmr1 silencing in male mice resulted in a delay in entering the nest (**D**) and less time spent inside (**E**). On the contrary, FMRP-deficient females (+/- and -/-) reached the nest more quickly (**D**) and spent more time inside (**E**) compared to their controls (+/+). In normal FMRP conditions, male mice (+/y) showed a shorter entry delay (**D**) and more time spent in the nest (**E**) than females (+/+). The number of entries into the nest did not differ among the experimental groups (**F**). (**A–F**) Data are shown as min. to max. box plots with median and 25–75 percentile. Single dots represent individual mice. Statistical analysis was done using Mann-Whitney U tests. *p*-values < 0.05 are indicated in the graphs. Full statistics can be found in Suppl. Table 13. Sample sizes: *+/y* males *N* = 10, *-/y* males *N* = 14, *+/+* females *N* = 6, *+/-* females *N* = 12 and *-/-* females *N* = 7

We next examined homing behavior, which represents the mice’s capacity to navigate back to a familiar location. It encompasses the integration of sensory input, spatial memory, and motor coordination [29–31]. Regarding locomotor activity, *Fmr1* silencing had a significant impact on female mice (Fig. 8A-C; Suppl. Table 13). FMRP-deficient females (*+/-* and *-/-*) exhibited increased locomotor activity compared to wild-type counterparts (*+/+*), as indicated by covering a greater distance (Fig. 8A; Suppl. Table 13), spending more time in motion (Fig. 8B; Suppl. Table 13), and maintaining a higher average speed (Fig. 8C; Suppl. Table 13). In contrast, there were no significant differences observed in locomotor activity between FMRP-deficient males and their wild-type counterparts (Fig. 8A-C; Suppl. Table 13). Additionally, under normal FMRP levels, male mice (*+/y*) traveled a greater distance and had a higher average speed compared to female mice (*+/+*) (Fig. 8A-C; Suppl. Table 13). In terms of homing behavior, sex-specific impairments were observed in FXS mice (Fig. 8D-F; Suppl. Table 13). Silencing of *Fmr1* gene in male mice led to an extended latency to enter the nest (Fig. 8D; Suppl. Table 13) and reduced time spent within the nest (Fig. 8E; Suppl. Table 13). Conversely, FMRP-deficient females (*+/-* and *-/-*) showed faster nest entry (Fig. 8D; Suppl. Table 13) and spent more time in the nest compared to their respective wild-type controls (Fig. 8E; Suppl. Table 13). Furthermore, male mice (*+/y*) with normal FMRP levels displayed a shorter latency (Fig. 8D; Suppl. Table 13) and spent more time in the nest than their female counterparts (*+/+*) (Fig. 8E; Suppl. Table 13). No significant differences were found in the number of entries into the nest among the different experimental groups (Fig. 8F; Suppl. Table 13). Overall, the evaluation of homing behavior revealed additional sex-specific effects of FXS, dependent on gene dosage.

## Discussion

The data illustrate the complex relationship between *Fmr1* gene dosage, sex, and communication development during infancy. FMRP-deficient pups of both sexes exhibited an increased tendency to vocalize when separated from their mothers. These vocalizations were accompanied by significant sex-specific changes in the main features of their USVs and the qualitative composition of ultrasonic communication in males.

To investigate the influence of FMRP on communication and homing behavior in both sexes, we generated and analyzed pups with various genotypes, including *+/y* and *-/y* males, as well as *+/+*, *+/-*, and *-/-* females. The *-/-* condition is rare in human females [32] and rodent data have been lacking.

During the early stages of NDDs, variations in metabolism and body weight are commonly observed [33]. In individuals with FXS, these alterations lead often to obesity as they age [34]. Previous studies in FXS mouse models have reported higher body weight in adult males (*-/y*) and females (*-/-*) compared to their respective controls [33]. Interestingly, our data show that male *-/y* mice had lower weight than *+/y* mice, while in females, *-/-* mice had higher weight than *+/+*. This suggests a sex-specific role of FMRP in metabolism in early life.

During early life, USVs serve as the primary mode of communication in rodents, providing a valuable window to gain insights into the initial stages of NDDs and ASDs [1–6]. Maternal separation is a widely utilized technique to evoke USVs in rodents. Here, we observed that maternal separation resulted in higher USV emission in both male and female FMRP-deficient mice. Interestingly, the latency for the first vocalization was decreased in females only. The increased tendency for vocalization in FXS mice suggests a unique emotional response to separation when compared to control mice [1, 5], as supported by studies indicating an elevated anxious phenotype in FXS male mice during this developmental period [24]. Children with FXS frequently display compulsive, repetitive, and perseverative speech patterns [15]. The heightened vocalization propensity observed in FMRP-deficient mice could be indicative of a similar characteristic within the pathology.

While the absence of FMRP led to an elevated vocalization rate in both males and females, the distinct features of these vocalizations displayed sex-specific variations: only males exhibited a longer average length of USVs when FMRP was absent, whereas females did not demonstrate this alteration. A separate study conducted in a different strain also reported sex-specific changes in vocalization length, suggesting a broader manifestation of this phenotypic trait [25]. A similar pattern of changes in both the quantity and quality of pup communication has been observed in the NF-κB p50-KO mice model of NDD. These mice emit a higher number of USVs and longer USVs when separated from their mothers, compared to control mice [35]. This shared characteristic across NDD models suggests that the amount and duration of USVs may reflect important aspects related to developmental status or cognitive abilities in mice. Further investigations are warranted to delve into the underlying implications of these alterations.

The lack of FMRP results in sex-specific differences in the likelihood of transitioning between various types of USVs in FXS pups. This finding is consistent with observations made in the development of oral communication in FXS children, who often exhibit limited expressive syntax [36–40]. Thus, we observed significant differences in the usage of ‘Downward Ramp’, ‘Complex Trill’, and ‘Short’ calls within the syntax of male *-/y* compared to *+/y* controls. In contrast, females exhibited a high degree of similarity in the syntactic usage of the ten different types of USVs across various genotypes. Our data together with the aforementioned alterations illustrate the sex-specific impact of the absence of FMRP on communication quality during early development: males are considerably more impacted than females. In human patients too, alterations are more pronounced in males than females [16] that do not display complexity deficits [40]. When assessing the expressive language capabilities of male and female patients with FXS, females typically perform better, although with considerable variation among individuals, partly attributed to differences in the activation ratios of the X chromosome [39, 41].

The effects of FXS on communication are generally less severe in females, primarily because they typically possess one unaltered gene copy [16, 39–41]. In our study, we also examined homozygous (*-/-*) females and noted that specific communication traits, such as vocalization propensity and the strength of USVs, were irregular in homozygous females but remained normal in heterozygous females. This suggests that having a single unmutated gene copy is sufficient to maintain normal communicative functions. Furthermore, the vocal repertoire and syntax of homozygous females were found to be preserved when compared to those of FXS males.

Homing behavior, serving as an early indicator of cognitive development and social discrimination, relies on motor skills and sensory cues for navigation and locating the maternal nest [29–31]. However, prior to our study, there was a lack of investigation into this behavior in FXS mouse. Our findings indicate that the *Fmr1* gene exerts a distinct, sex-specific influence on both locomotion and homing. In males, deficiency of FMRP leads to extended time to reach the nest while reducing the duration of stay, without affecting overall locomotion. These effects may be attributed to cognitive deficits, such as impaired integration of sensory stimuli and altered discrimination of maternal odor. On the other hand, the cognitive abilities of FXS females appear to be less impacted, and their increased locomotor activity may be related to altered anxiety or emotional states.

### Perspectives and significance

Given the swift and intricate developmental changes occurring in early postnatal life, confining the analysis to a restricted time period may overlook subtleties that a more expansive temporal perspective could unveil. To capture subtle nuances and understand the developmental trajectory of vocal repertoire, homing behavior, and other relevant behavioral, cellular, or molecular parameters in FXS mice, future research should extend the analysis across various postnatal ages. This comprehensive examination would shed light on progressive changes, uncover sensitive developmental stages, and enhance our understanding of FXS symptomatology. A full exploration of the interplay between the *Fmr1* gene, FMRP, and sex hormones during both pre- and postnatal stages could offer essential insights for developing therapeutic strategies based on patient sex.

In conclusion, this research underscores a significant interplay between the *Fmr1* gene and sex in shaping communicative and cognitive abilities in early life. It emphasizes the necessity of considering sex differences when comprehending the effects of FMRP deficiency and underscores the importance of adopting sex-specific approaches in the study of NDDs.

## Methods

### Animal

Animals were treated in compliance with the European Communities Council Directive (86/609/EEC) and the United States National Institutes of Health Guide for the care and use of laboratory animals. *Fmr1*-KO2 mice from FRAXA foundation were used in this study. Females *Fmr1+/-* were paired with males *Fmr1+/y* or *-/y* to obtain all genotypes included in this study (males *+/y*, males *-/y*, females *+/+*, females *+/-* and females *-/-*) (Fig. 1A). The male was removed from the cage after 1 week from the beginning of the mating (Fig. 1B). The behavioral tests were performed in male and female offspring during PND 10 and PND 13 (Fig. 1B). All mice used in this study were housed in standard wire-topped Plexiglas cages (42 × 27 × 14 cm) in a temperature and humidity-controlled condition (i.e., temperature 21 ± 1 °C, 60 ± 10% relative humidity and 12 h light/dark cycles). The nesting material was standardized providing 15 g of aspen pad and 1 compressed cotton stick. Food and water were available ad *libitum*. The French Ethical committee authorized this project (APAFIS#3279-2015121715284829-v6).

### Ultrasonic vocalizations

USVs were elicited through a rapid maternal separation procedure conducted on male and female pups at PND 10 [29]. Each mouse was individually placed in an empty plastic container measuring 11 × 7 × 3.5 cm, which was located inside a sound-attenuating isolation box. USVs were recorded using an ultrasonic microphone (Ultravox Noldus), connected via the Ultravox device (Noldus, Netherlands) and placed 20 cm above the pup in its plastic container. Following the 4-minute recording session, each pup was weighed, and a sample of tail tissue was collected for genotype determination. Changes in body temperature in the current experimental setting had been checked, reporting no significant change over the 4-minute test period.

The acoustic traces in the individual audio files were identified and studied using DeepSqueak [27] (version 2.6.2). This software converted the files into corresponding sonograms and utilized a Faster-RCNN object detector for analysis. To focus on the pertinent frequency range and reduce the interference of unrelated noise, a frequency band spanning from 20 to 100 kHz was set as the minimum and maximum cutoff frequencies, respectively. Each sonogram identified as noise was manually excluded. Automated USVs classification, pattern analysis and transition probabilities computation were performed in DeepSqueak through a neural network specifically designed for mouse call classification. This enabled identifying ten distinct vocalization types: Complex, Downward ramp, Inverted-U, Upward ramp, Complex trill, Short, Step Down, Flat, Step up, and Trill.

At first, the analysis of USVs assessed the propensity of each animal to vocalize. This involved tallying the number of USVs, while also studying the latency (s) and percentage of vocalizers (%). Following this, a more in-depth analysis was performed on animals demonstrating a baseline level of vocalizations, thus ensuring the software had enough data for detecting multiple transitions between USVs (> 1). This investigation included studying a range of characteristics of the USVs, such as their length (s), principal frequency (kHz), power (dB/Hz), and change in frequency (kHz). Additionally, the vocal repertoire and syntax were analyzed to understand overarching patterns and structure in the animal vocalizations.

### Homing test

The homing test was performed as published [29, 30]. At PND 13 both male and female pups were separated from the dam, and kept for 30 min in a different cage on a heating pad set at the temperature of 35 °C. Each tested mouse was placed in the Plexiglas cage (21 × 15 cm) which had one-third (7 × 15 cm) of the litter from the pup’s original cage and two-thirds (14 × 15 cm) of clean litter. The latter was considered as the unfamiliar area, while the one with the old litter was the nest area. The pup was located at the edge of the clean bedding and its behavior was videorecorded for the following 5 min. Homing performance was analyzed using Ethovision XT (Noldus) and considering the distance (cm), the moving time (s), the velocity (cm/s), the latency to reach the nest (s), the time spent (s) and the entries in the nest area.

### Statistical analysis

The datasets were assessed for normality using the D’Agostino-Pearson and Shapiro-Wilk tests. Given that none of the datasets met the prerequisites for parametric analyses, including normality and uniform sample sizes, the Mann–Whitney U test was employed for conducting statistical comparisons. GraphPad Prism 9 and DeepSqueak 2.6.2 were utilized for performing the statistics. The N values correspond to the number of animals tested in each group. Statistical significance was established at *p* < 0.05, with exact *p*-values indicated in the figures and tables.

**Supplementary Fig. 1. USV frequency distribution between sexes in the presence and absence of FMRP**. (**A**) Female controls use longer USVs more frequently than male controls. (**B**) In absence of FMRP, females use medium-duration (~ 0.02s) USVs more frequently than males. (**C**) In control groups, males exhibit a relatively higher proportion of higher frequencies compared to females, (**D**) but when FMRP is absent, this distribution becomes similar between sexes. (**E**) The USVs power is similar between male and female controls. (**F**) When FMRP is lacking females use more vocalization with an average power of ~-75 dB/Hz compared with males. (**G, H**) While among control pups males use more frequently smaller Δ frequency compared to females (**G**), this disparity diminishes in FMRP deficient mice (**H**). (**A–H**) Data are represented as Gaussian curve fit (± CI) of the frequency distribution (%). Sample size: *+/y* males *N* = 9, *-/y* males *N* = 14, *+/+* females *N* = 7, *+/-* females *N* = 13 and *-/-* females *N* = 6.

**Supplementary Fig. 2. Patterns of USV transition probabilities vary with sexes and genotypes**. Transition probabilities heat maps of (**A**) males (*+/y* and *-/y*) and (**B**) females (*+/+*, *+/-*, *-/-*) syntax. (**A, B**) Values in individual boxes indicate the probability of one call to follow the previous. The transition probability is expressed as the mean probability of each transition for each group. Sample size: *+/y* males *N* = 9, *-/y* males *N* = 14, *+/+*. *+/+* females *N* = 7, *+/-* females *N* = 13 and *-/-* females *N* = 6.

### Electronic supplementary material

Below is the link to the electronic supplementary material.

**Supplementary Material 1**: **Supplementary table 1. Body weight in FXS pups at PND 10 and 13**. Comparison of body weight (g) among groups. All *p*-values are shown in the table, bold when *p* < 0.05. Mann-Whitney U tests

**Supplementary Material 2**: **Supplementary table 2. Vocalization propensity during early postnatal life of FXS mice**. Comparison of number of emitted USVs and latency (s) among groups. All *p*-values are shown in the table, bold when *p* < 0.05. Mann-Whitney U tests

**Supplementary Material 3**: **Supplementary table 3. Core features of USVs in FXS mice**. Comparison of length (s), principal frequency (kHz), power (dB/Hz) and Δ frequency (kHz) among groups. All *p*-values are shown in the table, bold when *p* < 0.05. Mann-Whitney U tests

**Supplementary Material 4**: **Supplementary table 4. Vocal repertoire of Fmr1 +/y and -/y males at PND 10**. Comparison of percentage use among different types of USVs within the *+/y* (**A**) and *-/y* (**B**) male groups. All *p*-values are shown in the table, bold when *p* < 0.05. Mann-Whitney U tests. 1 = Complex, 2 = Downward Ramp, 3 = Inverted-U, 4 = Upward Ramp, 5 = Complex Trill, 6 = Short, 7 = Step Down, 8 = Flat, 9 = Step Up, 10 = Trill

**Supplementary Material 5**: **Supplementary table 5. Vocal repertoire of Fmr1 +/+, +/- and -/- females at PND 10**. Comparison of percentage use among different types of USVs within the *+/+* (**A**), *+/-* (**B**) and *-/-* (**C**) female groups. All *p*-values are shown in the table, bold when *p* < 0.05. Mann-Whitney U tests. 1 = Complex, 2 = Downward Ramp, 3 = Inverted-U, 4 = Upward Ramp, 5 = Complex Trill, 6 = Short, 7 = Step Down, 8 = Flat, 9 = Step Up, 10 = Trill

**Supplementary Material 6**: **Supplementary table 6. Comparison of vocal repertoire between sexes and genotypes**. Comparison of percentage use of different types of USVs by sex and genotype. All *p*-values are shown in the table, bold when *p* < 0.05. Mann-Whitney U tests

**Supplementary Material 7**: **Supplementary table 7. Transition probability from different USVs in males**. Comparison among transition probabilities from different types of USVs within the *+/y* (**A**) and *-/y* (**B**) male groups. All *p*-values are shown in the table, bold when *p* < 0.05. Mann-Whitney U tests. 1 = Complex, 2 = Downward Ramp, 3 = Inverted-U, 4 = Upward Ramp, 5 = Complex Trill, 6 = Short, 7 = Step Down, 8 = Flat, 9 = Step Up, 10 = Trill

**Supplementary Material 8**: **Supplementary table 8. Transition probability from different USVs in females**. Comparison among the transition probabilities from different types of USVs within the *+/+* (**A**), *+/-* (**B)** and *-/-* (**C**) female groups. All *p*-values are shown in the table, bold when *p* < 0.05. Mann-Whitney U tests. 1 = Complex, 2 = Downward Ramp, 3 = Inverted-U, 4 = Upward Ramp, 5 = Complex Trill, 6 = Short, 7 = Step Down, 8 = Flat, 9 = Step Up, 10 = Trill

**Supplementary Material 9**: **Supplementary Table 9. Comparison of transition probability from different USVs.** Comparison transition probabilities from different types of USVs by sex and genotype. All *p*-values are shown in the table, bold when *p* < 0.05. Mann-Whitney U tests

**Supplementary Material 10**: **Supplementary table 10. Transition probability to different USVs in males**. Comparison among transition probabilities to different types of USVs within the *+/y* (**A**) and *-/y* (**B**) male groups. All *p*-values are shown in the table, bold when *p* < 0.05. Mann-Whitney U tests. 1 = Complex, 2 = Downward Ramp, 3 = Inverted-U, 4 = Upward Ramp, 5 = Complex Trill, 6 = Short, 7 = Step Down, 8 = Flat, 9 = Step Up, 10 = Trill

**Supplementary Material 11**: **Supplementary table 11. Transition probability to different USVs in females**. Comparison among the transition probabilities to different types of USVs within the *+/+* (**A**), *+/-* (**B**) and *-/-* (**C**) female groups. All *p*-values are shown in the table, bold when *p* < 0.05. Mann-Whitney U tests. 1 = Complex, 2 = Downward Ramp, 3 = Inverted-U, 4 = Upward Ramp, 5 = Complex Trill, 6 = Short, 7 = Step Down, 8 = Flat, 9 = Step Up, 10 = Trill

**Supplementary Material 12**: **Supplementary Table 12. Comparison of transition probability to different USVs.** Comparison transition probabilities to different types of USVs by sex and genotype. All *p*-values are shown in the table. Mann-Whitney U tests

**Supplementary Material 13**: **Supplementary table 13. Homing behavior in FXS mice**. Comparison of total distance (cm), moving time (s), velocity (cm/s), latency to nest (s), time spent in the nest (s) and nest entries (#) among groups. All *p*-values are shown in the table, bold when *p* < 0.05. Mann-Whitney U tests

**Supplementary Material 14**

**Supplementary Material 15**

**Supplementary Material 16**

## Data Availability

All data reported in this paper will be shared by the lead contact upon request. This paper does not report original code. Any additional information required to reanalyze the data reported in this paper is available from the lead contact upon request.

## References

[1] Granon S, Faure A, Chauveau F, Cressant A, Ey E (2018). Why should my mouse call me? Acoustic Communication in Mouse models of Social disorders: Ultrasonic vocalizations as an index of emotional and motivational States. Handb Behav Neurosci.

[2] Moy SS, Nadler JJ (2008). Advances in behavioral genetics: mouse models of autism. Mol Psychiatry.

[3] Scattoni ML, Crawley J, Ricceri L (2009). Ultrasonic vocalizations: a tool for behavioural phenotyping of mouse models of neurodevelopmental disorders. Neurosci Biobehav Rev.

[4] Fischer J, Hammerschmidt K (2011). Ultrasonic vocalizations in mouse models for speech and socio-cognitive disorders: insights into the evolution of vocal communication. Genes Brain Behav.

[5] Simola N, Granon S. Ultrasonic vocalizations as a tool in studying emotional states in rodent models of social behavior and brain disease. Neuropharmacology 2019;159. 10.1016/j.neuropharm.2018.11.008 30445100

[6] Premoli M, Memo M, Bonini S (2021). Ultrasonic vocalizations in mice: relevance for ethologic and neurodevelopmental disorders studies. Neural Regen Res.

[7] Holy TE, Guo Z (2005). Ultrasonic songs of male mice. PLoS Biol.

[8] Bell RW, Nitschke W, Zachman TA (1972). Ultra-sounds in three inbred strains of young mice. Behav Biol.

[9] Caruso A, Marconi MA, Scattoni ML, Ricceri L (2022). Ultrasonic vocalizations in laboratory mice: strain, age, and sex differences. Genes Brain Behav.

[10] Zippelius HM, Schleidt WM (1956). Ultraschall-Laute Bei Jungen Mäusen. Naturwissenschaften.

[11] Hunter J, Rivero-Arias O, Angelov A, Kim E, Fotheringham I, Leal J (2014). Epidemiology of fragile X syndrome: a systematic review and meta-analysis. Am J Med Genet A.

[12] Wiśniowiecka-Kowalnik B, Nowakowska BA (2019). Genetics and epigenetics of autism spectrum disorder-current evidence in the field. J Appl Genet.

[13] Hagerman RJ, Berry-Kravis E, Hazlett HC, Bailey DB, Moine H, Kooy RF (2017). Fragile X syndrome. Nat Rev Dis Prim.

[14] Hoffmann A. Communication in fragile X syndrome: patterns and implications for assessment and intervention. Front Psychol 2022;13. 10.3389/fpsyg.2022.929379 PMC9817301 36619013

[15] Finestack LH, Richmond EK, Abbeduto L (2009). Language Development in individuals with fragile X syndrome. Top Lang Disord.

[16] Brady N, Skinner D, Roberts J, Hennon E (2006). Communication in young children with fragile X syndrome: a qualitative study of mothers’ perspectives. Am J speech-language Pathol.

[17] Brady NC, Fleming K, Bredin-Oja SL, Fielding-Gebhardt H, Warren SF (2020). Language Development from Early Childhood to Adolescence in youths with Fragile X Syndrome. J Speech Lang Hear Res.

[18] Roy S, Watkins N, Heck D. Comprehensive analysis of ultrasonic vocalizations in a mouse model of fragile X syndrome reveals limited, call type specific deficits. PLoS ONE 2012;7. 10.1371/journal.pone.0044816 PMC3439444 22984567

[19] Belagodu AP, Johnson AM, Galvez R (2016). Characterization of ultrasonic vocalizations of Fragile X mice. Behav Brain Res.

[20] Toledo MA, Wen TH, Binder DK, Ethell IM, Razak KA. Reversal of ultrasonic vocalization deficits in a mouse model of Fragile X syndrome with minocycline treatment or genetic reduction of MMP-9. Behav Brain Res 2019;372. 10.1016/j.bbr.2019.112068 PMC6662633 31271818

[21] Prieto M, Folci A, Poupon G, Schiavi S, Buzzelli V, Pronot M, et al. Missense mutation of Fmr1 results in impaired AMPAR-mediated plasticity and socio-cognitive deficits in mice. Nat Commun. 2021;12. 10.1038/S41467-021-21820-1. 10.1038/s41467-021-21820-1 PMC7946954 33692361

[22] Lai JKY, Sobala-Drozdowski M, Zhou L, Doering LC, Faure PA, Foster JA (2014). Temporal and spectral differences in the ultrasonic vocalizations of fragile X knock out mice during postnatal development. Behav Brain Res.

[23] Reynolds CD, Nolan SO, Jefferson T, Lugo JN (2016). Sex-specific and genotype-specific differences in vocalization development in FMR1 knockout mice. NeuroReport.

[24] Gaudissard J, Ginger M, Premoli M, Memo M, Frick A, Pietropaolo S (2017). Behavioral abnormalities in the Fmr1-KO2 mouse model of fragile X syndrome: the relevance of early life phases. Autism Res.

[25] Nolan SO, Hodges SL, Lugo JN. High-throughput analysis of vocalizations reveals sex-specific changes in Fmr1 mutant pups. Genes Brain Behav 2020;19. 10.1111/gbb.12611 PMC12939262 31587487

[26] Hodges SL, Nolan SO, Reynolds CD, Lugo JN (2017). Spectral and temporal properties of calls reveal deficits in ultrasonic vocalizations of adult Fmr1 knockout mice. Behav Brain Res.

[27] Coffey KR, Marx RG, Neumaier JF (2019). DeepSqueak: a deep learning-based system for detection and analysis of ultrasonic vocalizations. Neuropsychopharmacology.

[28] Mientjes EJ, Nieuwenhuizen I, Kirkpatrick L, Zu T, Hoogeveen-Westerveld M, Severijnen L (2006). The generation of a conditional Fmr1 knock out mouse model to study Fmrp function in vivo. Neurobiol Dis.

[29] Iezzi D, Caceres-Rodriguez A, Chavis P, Manzoni OJJ. In utero exposure to cannabidiol disrupts select early-life behaviors in a sex-specific manner. Transl Psychiatry 2022;12. 10.1038/s41398-022-02271-8 PMC9722662 36470874

[30] Manduca A, Servadio M, Melancia F, Schiavi S, Manzoni OJ, Trezza V (2020). Sex-specific behavioural deficits induced at early life by prenatal exposure to the cannabinoid receptor agonist WIN55, 212-2 depend on mGlu5 receptor signalling. Br J Pharmacol.

[31] Bignami G (1996). Economical test methods for developmental neurobehavioral toxicity. Environ Health Perspect.

[32] Vafaeie F, Alerasool M, Kaseb Mojaver N, Mojarrad M. Fragile X syndrome in a female with homozygous full-mutation alleles of the FMR1 gene. Cureus 2021;13. 10.7759/cureus.16340 PMC8357243 34395123

[33] Menzies C, Naz S, Patten D, Lacoste B, Alquier T, Bennett BM. Distinct Basal Metabolism in Three Mouse Models of Neurodevelopmental Disorders. *eNeuro* 2021;8. 10.1523/ENEURO.0292-20.2021 PMC8174051 33820803

[34] Raspa M, Bailey DB, Bishop E, Holiday D, Olmsted M (2010). Obesity, food selectivity, and physical activity in individuals with fragile X syndrome. Am J Intellect Dev Disabil.

[35] Premoli M, Bonini SA, Mastinu A, Maccarinelli G, Aria F, Paiardi G et al. Specific profile of ultrasonic communication in a mouse model of neurodevelopmental disorders. Sci Rep 2019;9. 10.1038/s41598-019-52378-0 PMC6828716 31685905

[36] Martin GE, Losh M, Estigarribia B, Sideris J, Roberts J (2013). Longitudinal profiles of expressive vocabulary, syntax and pragmatic language in boys with fragile X syndrome or down syndrome. Int J Lang Commun Disord.

[37] Price JR, Roberts JE, Hennon EA, Berni MC, Anderson KL, Sideris J (2008). Syntactic complexity during conversation of boys with fragile X syndrome and Down syndrome. J Speech Lang Hear Res.

[38] Roberts J, Martin GE, Moskowitz L, Harris AA, Foreman J, Nelson L (2007). Discourse skills of boys with fragile X syndrome in comparison to boys with Down syndrome. J Speech Lang Hear Res.

[39] Komesidou R, Brady NC, Fleming K, Esplund A, Warren SF (2017). Growth of expressive syntax in Children with Fragile X Syndrome. J Speech Lang Hear Res.

[40] Kover ST, Abbeduto L (2019). Syntactic ability of girls with fragile X syndrome: phonological memory and discourse demands on Complex Sentence Use. Am J Intellect Dev Disabil.

[41] Finestack LH, Abbeduto L (2010). Expressive language profiles of verbally expressive adolescents and young adults with Down syndrome or fragile X syndrome. J Speech Lang Hear Res.

