# Supplementary material for: Sex-specific modulation of early life vocalization and cognition by Fmr1 gene dosage in a mouse model of Fragile X Syndrome
Source: Biol Sex Differ. 2024 Feb 21;15:18. doi: 10.1186/s13293-024-00594-3 (PMC10880250; doi:10.1186/s13293-024-00594-3)
Supplement: Supplementary file 1 — Supplementary Material 1: Supplementary table 1. Body weight in FXS pups at PND 10 and 13. Comparison of body weight (g) among groups. All p-values are shown in the table, bold when p < 0.05. Mann-Whitney U tests [file 13293_2024_594_MOESM1_ESM.docx]

|  | **Sex** | ***Fmr1*** | **Median** | **Max** | **Min** | **N** | ***+/y*  VS  *-/y*** | ***+/y*  VS  *+/+*** | ***-/y*  VS  *-/-*** | ***+/+*  VS  *+/-*** | ***+/+*  VS  *-/-*** | ***+/-*  VS  *-/-*** |
| --- | --- | --- | --- | --- | --- | --- | --- | --- | --- | --- | --- | --- |
|  |  |  |  |  |  |  | **p-value** | | | | | |
| PND 10 | M | *+/y* | 5.1 | 6.4 | 4.0 | 22 | **0.0471** | **0.0254** | 0.0535 | 0.1076 | **0.0143** | 0.2892 |
|  | M | *-/y* | 4.7 | 5.5 | 3.6 | 21 |  |  |  |  |  |  |
|  | F | *+/+* | 4.4 | 5.5 | 3.6 | 12 |  |  |  |  |  |  |
|  | F | *+/-* | 5.0 | 6.8 | 3.5 | 26 |  |  |  |  |  |  |
|  | F | *-/-* | 5.2 | 7.1 | 4.7 | 6 |  |  |  |  |  |  |
|  |  |  |  |  |  |  |  |  |  |  |  |  |
| PND 13 | M | *+/y* | 6.6 | 7.3 | 5.8 | 10 | **0.0340** | 0.3241 | **0.0021** | 0.2986 | **0.0472** | 0.3089 |
|  | M | *-/y* | 6.1 | 6.7 | 4.8 | 14 |  |  |  |  |  |  |
|  | F | *+/+* | 6.3 | 6.7 | 5.4 | 6 |  |  |  |  |  |  |
|  | F | *+/-* | 6.6 | 7.4 | 5.0 | 12 |  |  |  |  |  |  |
|  | F | *-/-* | 6.7 | 7.3 | 6.2 | 7 |  |  |  |  |  |  |

**Supplementary Table 1. Body weight in FXS pups at PND 10 and PND 13.**

Comparison of body weight (g) among groups. All p-values are shown in the table, bold when p < 0.05. Mann-Whitney *U* tests.
