# Supplementary material for: Sex-specific modulation of early life vocalization and cognition by Fmr1 gene dosage in a mouse model of Fragile X Syndrome
Source: Biol Sex Differ. 2024 Feb 21;15:18. doi: 10.1186/s13293-024-00594-3 (PMC10880250; doi:10.1186/s13293-024-00594-3)
Supplement: Supplementary file 2 — Supplementary Material 2: Supplementary table 2. Vocalization propensity during early postnatal life of FXS mice. Comparison of number of emitted USVs and latency (s) among groups. All p-values are shown in the table, bold when p < 0.05. Mann-Whitney U tests [file 13293_2024_594_MOESM2_ESM.docx]

|  | **Sex** | ***Fmr1*** | **Median** | **Max** | **Min** | **N** | ***+/y*  VS  *-/y*** | ***+/y*  VS  *+/+*** | ***-/y*  VS  *-/-*** | ***+/+*  VS  *+/-*** | ***+/+*  VS  *-/-*** | ***+/-*  VS  *-/-*** |
| --- | --- | --- | --- | --- | --- | --- | --- | --- | --- | --- | --- | --- |
|  |  |  |  |  |  |  | **p-value** | | | | | |
| N. of USVs | M | *+/y* | 4.50 | 29.00 | 0.00 | 22 | **0.0160** | 0.1622 | **0.0314** | 0.6798 | **0.0499** | **0.0237** |
|  | M | *-/y* | 20.00 | 73.00 | 0.00 | 21 |  |  |  |  |  |  |
|  | F | *+/+* | 10.50 | 74.00 | 0.00 | 12 |  |  |  |  |  |  |
|  | F | *+/-* | 4.50 | 159.00 | 0.00 | 26 |  |  |  |  |  |  |
|  | F | *-/-* | 35.00 | 73.00 | 21.00 | 6 |  |  |  |  |  |  |
| Latency (s) | M | *+/y* | 44.57 | 213.30 | 4.24 | 17 | 0.2874 | 0.9803 | 0.2509 | 0.3069 | **0.0420** | 0.4942 |
|  | M | *-/y* | 37.80 | 174.10 | 0.44 | 18 |  |  |  |  |  |  |
|  | F | *+/+* | 45.45 | 124.90 | 2.20 | 10 |  |  |  |  |  |  |
|  | F | *+/-* | 16.20 | 228.20 | 0.68 | 20 |  |  |  |  |  |  |
|  | F | *-/-* | 14.18 | 65.43 | 0.20 | 6 |  |  |  |  |  |  |

**Supplementary Table 2. Vocalization propensity during early postnatal life of FXS mice.**

Comparison of number of emitted USVs and latency (s) among groups. All p-values are shown in the table, bold when p < 0.05. Mann-Whitney *U* tests.
