# Supplementary material for: Sex-specific modulation of early life vocalization and cognition by Fmr1 gene dosage in a mouse model of Fragile X Syndrome
Source: Biol Sex Differ. 2024 Feb 21;15:18. doi: 10.1186/s13293-024-00594-3 (PMC10880250; doi:10.1186/s13293-024-00594-3)
Supplement: Supplementary file 3 — Supplementary Material 3: Supplementary table 3. Core features of USVs in FXS mice. Comparison of length (s), principal frequency (kHz), power (dB/Hz) and Δ frequency (kHz) among groups. All p-values are shown in the table, bold when p < 0.05. Mann-Whitney U tests [file 13293_2024_594_MOESM3_ESM.docx]

|  | **Sex** | ***Fmr1*** | **Median** | **Max** | **Min** | **N** | ***+/y*  VS  *-/y*** | ***+/y*  VS  *+/+*** | ***-/y*  VS  *-/-*** | ***+/+*  VS  *+/-*** | ***+/+*  VS  *-/-*** | ***+/-*  VS  *-/-*** |
| --- | --- | --- | --- | --- | --- | --- | --- | --- | --- | --- | --- | --- |
|  |  |  |  |  |  |  | **p-value** | | | | | |
| Length (s) | M | *+/y* | 0.0211 | 0.0277 | 0.0119 | 9 | **0.0327** | **0.0229** | 0.4442 | 0.4854 | 0.1375 | 0.4155 |
|  | M | *-/y* | 0.0260 | 0.0562 | 0.0185 | 14 |  |  |  |  |  |  |
|  | F | *+/+* | 0.0268 | 0.0372 | 0.0235 | 7 |  |  |  |  |  |  |
|  | F | *+/-* | 0.0272 | 0.0849 | 0.0161 | 13 |  |  |  |  |  |  |
|  | F | *-/-* | 0.0240 | 0.0324 | 0.0183 | 6 |  |  |  |  |  |  |
| Pr. Freq. (kHz) | M | *+/y* | 61.37 | 63.35 | 52.33 | 9 | 0.6883 | 0.1416 | 0.4442 | 0.5880 | 0.2949 | 0.3229 |
|  | M | *-/y* | 61.13 | 69.23 | 54.62 | 14 |  |  |  |  |  |  |
|  | F | *+/+* | 56.62 | 60.03 | 54.62 | 7 |  |  |  |  |  |  |
|  | F | *+/-* | 57.97 | 61.47 | 50.06 | 13 |  |  |  |  |  |  |
|  | F | *-/-* | 59.35 | 61.63 | 55.66 | 6 |  |  |  |  |  |  |
| Power (dB/(Hz) | M | *+/y* | -72.63 | -70.24 | -75.91 | 9 | 0.2244 | 0.2991 | 0.7181 | 0.3929 | **0.0350** | **0.0462** |
|  | M | *-/y* | -74.86 | -69.31 | -78.17 | 14 |  |  |  |  |  |  |
|  | F | *+/+* | -70.32 | -68.65 | -76.10 | 7 |  |  |  |  |  |  |
|  | F | *+/-* | -72.69 | -61.52 | -75.60 | 13 |  |  |  |  |  |  |
|  | F | *-/-* | -75.13 | -72.63 | -77.09 | 6 |  |  |  |  |  |  |
| Δ freq. (kHz) | M | *+/y* | 5.667 | 8.143 | 2.241 | 9 | 0.1093 | 0.0712 | 0.2074 | 0.8773 | 0.3660 | 0.4670 |
|  | M | *-/y* | 6.749 | 10.890 | 4.753 | 14 |  |  |  |  |  |  |
|  | F | *+/+* | 7.210 | 8.876 | 5.190 | 7 |  |  |  |  |  |  |
|  | F | *+/-* | 7.181 | 12.370 | 3.434 | 13 |  |  |  |  |  |  |
|  | F | *-/-* | 8.362 | 10.900 | 5.914 | 6 |  |  |  |  |  |  |

**Supplementary Table 3. Core features of USVs in FXS mice.**

Comparison of length (s), principal frequency (kHz), power (dB/Hz) and Δ frequency (kHz) among groups. All p-values are shown in the table, bold when p < 0.05. Mann-Whitney *U* tests.
