# Supplementary material for: Sex-specific modulation of early life vocalization and cognition by Fmr1 gene dosage in a mouse model of Fragile X Syndrome
Source: Biol Sex Differ. 2024 Feb 21;15:18. doi: 10.1186/s13293-024-00594-3 (PMC10880250; doi:10.1186/s13293-024-00594-3)
Supplement: Supplementary file 4 — Supplementary Material 4: Supplementary table 4. Vocal repertoire of Fmr1 +/y and -/y males at PND 10. Comparison of percentage use among different types of USVs within the +/y (A) and -/y (B) male groups. All p-values are shown in the table, bold when p < 0.05. Mann-Whitney U tests. 1 = Complex, 2 = Downward Ramp, 3 = Inverted-U, 4 = Upward Ramp, 5 = Complex Trill, 6 = Short, 7 = Step Down, 8 = Flat, 9 = Step Up, 10 = Trill [file 13293_2024_594_MOESM4_ESM.docx]

| **A** | **1** | **2** | **3** | **4** | **5** | **6** | **7** | **8** | **9** | **10** |
| --- | --- | --- | --- | --- | --- | --- | --- | --- | --- | --- |
| **1** |  | 0.0805 | 0.4900 | 0.6084 | 0.5283 | 0.9527 | **0.0350** | 0.8112 | **0.0158** | 0.4215 |
| **2** | 0.0805 |  | **0.0187** | **0.0248** | 0.2485 | 0.0649 | **0.0009** | 0.1127 | **0.0004** | **0.0095** |
| **3** | 0.4900 | **0.0187** |  | 0.9931 | 0.2077 | 0.4892 | 0.1041 | 0.3460 | 0.0701 | 0.9395 |
| **4** | 0.6084 | **0.0248** | 0.9931 |  | 0.2261 | 0.3582 | 0.1312 | 0.2999 | **0.0498** | 0.9712 |
| **5** | 0.5283 | 0.2485 | 0.2077 | 0.2261 |  | 0.6784 | **0.0044** | 0.8142 | **0.0012** | 0.1922 |
| **6** | 0.9527 | 0.0649 | 0.4892 | 0.3582 | 0.6784 |  | **0.0300** | 0.7768 | **0.0090** | 0.3789 |
| **7** | **0.0350** | **0.0009** | 0.1041 | 0.1312 | **0.0044** | **0.0300** |  | **0.0108** | 0.7353 | 0.1041 |
| **8** | 0.8112 | 0.1127 | 0.3460 | 0.2999 | 0.8142 | 0.7768 | **0.0108** |  | **0.0041** | 0.2610 |
| **9** | **0.0158** | **0.0004** | 0.0701 | **0.0498** | **0.0012** | **0.0090** | 0.7353 | **0.0041** |  | **0.0498** |
| **10** | 0.4215 | **0.0095** | 0.9395 | 0.9712 | 0.1922 | 0.3789 | 0.1041 | 0.2610 | **0.0498** |  |
|  |  |  |  |  |  |  |  |  |  |  |
| **B** | **1** | **2** | **3** | **4** | **5** | **6** | **7** | **8** | **9** | **10** |
| **1** |  | **<0.0001** | 0.6896 | 0.3797 | **0.0428** | **0.0042** | 0.0951 | 0.9368 | **0.0002** | 0.1714 |
| **2** | **<0.0001** |  | **<0.0001** | **<0.0001** | **0.0018** | **<0.0001** | **<0.0001** | **<0.0001** | **<0.0001** | **<0.0001** |
| **3** | 0.6896 | **<0.0001** |  | 0.8121 | **0.0067** | **0.0036** | 0.1177 | 0.4069 | **<0.0001** | 0.1817 |
| **4** | 0.3797 | **<0.0001** | 0.8121 |  | **0.0010** | **0.0011** | 0.1248 | 0.1007 | **<0.0001** | 0.2943 |
| **5** | **0.0428** | **0.0018** | **0.0067** | **0.0010** |  | **<0.0001** | **<0.0001** | **0.0383** | **<0.0001** | **0.0006** |
| **6** | **0.0042** | **<0.0001** | **0.0036** | **0.0011** | **<0.0001** |  | 0.0702 | **0.0001** | 0.0978 | 0.1214 |
| **7** | 0.0951 | **<0.0001** | 0.1177 | 0.1248 | **<0.0001** | 0.0702 |  | **0.0063** | **0.0014** | 0.9906 |
| **8** | 0.9368 | **<0.0001** | 0.4069 | 0.1007 | **0.0383** | **0.0001** | **0.0063** |  | **<0.0001** | **0.0347** |
| **9** | **0.0002** | **<0.0001** | **<0.0001** | **<0.0001** | **<0.0001** | 0.0978 | **0.0014** | **<0.0001** |  | **0.0046** |
| **10** | 0.1714 | **<0.0001** | 0.1817 | 0.2943 | **0.0006** | 0.1214 | 0.9906 | **0.0347** | **0.0046** |  |

**Supplementary Table 4. Vocal repertoire of *Fmr1 +/y and -/y* males at PND 10**

Comparison of percentage use among different types of USVs within the *+/y* **(A)** and *-/y* **(B)** male groups. All p-values are shown in the table, bold when p < 0.05. Mann-Whitney *U* tests. 1= Complex, 2=Downward Ramp, 3= Inverted-U, 4= Upward Ramp, 5= Complex Trill, 6= Short, 7= Step Down, 8= Flat, 9= Step Up, 10=Trill.
