# Supplementary material for: Sex-specific modulation of early life vocalization and cognition by Fmr1 gene dosage in a mouse model of Fragile X Syndrome
Source: Biol Sex Differ. 2024 Feb 21;15:18. doi: 10.1186/s13293-024-00594-3 (PMC10880250; doi:10.1186/s13293-024-00594-3)
Supplement: Supplementary file 5 — Supplementary Material 5: Supplementary table 5. Vocal repertoire of Fmr1 +/+, +/- and -/- females at PND 10. Comparison of percentage use among different types of USVs within the +/+ (A), +/- (B) and -/- (C) female groups. All p-values are shown in the table, bold when p < 0.05. Mann-Whitney U tests. 1 = Complex, 2 = Downward Ramp, 3 = Inverted-U, 4 = Upward Ramp, 5 = Complex Trill, 6 = Short, 7 = Step Down, 8 = Flat, 9 = Step Up, 10 = Trill [file 13293_2024_594_MOESM5_ESM.docx]

| **A** | **1** | **2** | **3** | **4** | **5** | **6** | **7** | **8** | **9** | **10** |
| --- | --- | --- | --- | --- | --- | --- | --- | --- | --- | --- |
| **1** |  | 0.0973 | 0.5530 | **0.0012** | 0.1037 | **0.0006** | **0.0029** | 0.8048 | **0.0006** | **0.0070** |
| **2** | 0.0973 |  | 0.2197 | **0.0006** | **0.0029** | **0.0006** | **0.0006** | **0.0379** | **0.0006** | **0.0012** |
| **3** | 0.5530 | 0.2197 |  | 0.0915 | 0.3013 | 0.0554 | 0.0554 | 0.6177 | **0.0210** | 0.0915 |
| **4** | **0.0012** | **0.0006** | 0.0915 |  | **0.0047** | 0.5105 | >0.9999 | **0.0117** | 0.1678 | 0.6853 |
| **5** | 0.1037 | **0.0029** | 0.3013 | **0.0047** |  | **0.0006** | **0.0291** | 0.3339 | **0.0006** | 0.0763 |
| **6** | **0.0006** | **0.0006** | 0.0554 | 0.5105 | **0.0006** |  | 0.7552 | **0.0099** | 0.4371 | 0.4167 |
| **7** | **0.0029** | **0.0006** | 0.0554 | >0.9999 | **0.0291** | 0.7552 |  | **0.0262** | 0.3147 | 0.6329 |
| **8** | 0.8048 | **0.0379** | 0.6177 | **0.0117** | 0.3339 | **0.0099** | **0.0262** |  | **0.0047** | 0.0664 |
| **9** | **0.0006** | **0.0006** | **0.0210** | 0.1678 | **0.0006** | 0.4371 | 0.3147 | **0.0047** |  | 0.1189 |
| **10** | **0.0070** | **0.0012** | 0.0915 | 0.6853 | 0.0763 | 0.4167 | 0.6329 | 0.0664 | 0.1189 |  |
|  |  |  |  |  |  |  |  |  |  |  |
| **B** | **1** | **2** | **3** | **4** | **5** | **6** | **7** | **8** | **9** | **10** |
| **1** |  | **0.0033** | 0.2363 | **0.0007** | 0.3159 | **0.0009** | **0.0005** | **0.0425** | **<0.0001** | **0.0013** |
| **2** | **0.0033** |  | **0.0004** | **<0.0001** | **0.0002** | **<0.0001** | **<0.0001** | **<0.0001** | **<0.0001** | **<0.0001** |
| **3** | 0.2363 | **0.0004** |  | **0.0023** | 0.9895 | **0.0016** | **0.0010** | 0.2805 | **<0.0001** | **0.0052** |
| **4** | **0.0007** | **<0.0001** | **0.0023** |  | **0.0001** | 0.3312 | 0.5193 | **0.0056** | **0.0124** | 0.9272 |
| **5** | 0.3159 | **0.0002** | 0.9895 | **0.0001** |  | **0.0002** | **<0.0001** | 0.1808 | **<0.0001** | **0.0004** |
| **6** | **0.0009** | **<0.0001** | **0.0016** | 0.3312 | **0.0002** |  | 0.7204 | **0.0016** | 0.1268 | 0.5071 |
| **7** | **0.0005** | **<0.0001** | **0.0010** | 0.5193 | **<0.0001** | 0.7204 |  | **0.0019** | 0.0520 | 0.6035 |
| **8** | **0.0425** | **<0.0001** | 0.2805 | **0.0056** | 0.1808 | **0.0016** | **0.0019** |  | **<0.0001** | **0.0161** |
| **9** | **<0.0001** | **<0.0001** | **<0.0001** | **0.0124** | **<0.0001** | 0.1268 | 0.0520 | **<0.0001** |  | **0.0369** |
| **10** | **0.0013** | **<0.0001** | **0.0052** | 0.9272 | **0.0004** | 0.5071 | 0.6035 | **0.0161** | **0.0369** |  |
|  |  |  |  |  |  |  |  |  |  |  |
| **C** | **1** | **2** | **3** | **4** | **5** | **6** | **7** | **8** | **9** | **10** |
| **1** |  | **0.0303** | 0.4589 | 0.4134 | 0.9091 | **0.0130** | 0.0714 | 0.0866 | **0.0087** | **0.0065** |
| **2** | **0.0303** |  | 0.0649 | **0.0087** | **0.0152** | **0.0022** | **0.0065** | **0.0022** | **0.0022** | **0.0022** |
| **3** | 0.4589 | 0.0649 |  | 0.1948 | 0.4632 | **0.0022** | **0.0260** | **0.0065** | **0.0022** | **0.0022** |
| **4** | 0.4134 | **0.0087** | 0.1948 |  | 0.5130 | 0.1450 | 0.5628 | 0.4545 | 0.0996 | 0.0606 |
| **5** | 0.9091 | **0.0152** | 0.4632 | 0.5130 |  | **0.0087** | 0.1061 | 0.1515 | **0.0087** | **0.0065** |
| **6** | **0.0130** | **0.0022** | **0.0022** | 0.1450 | **0.0087** |  | 0.2749 | **0.0346** | >0.9999 | 0.7273 |
| **7** | 0.0714 | **0.0065** | **0.0260** | 0.5628 | 0.1061 | 0.2749 |  | 0.2576 | 0.3074 | 0.1970 |
| **8** | 0.0866 | **0.0022** | **0.0065** | 0.4545 | 0.1515 | **0.0346** | 0.2576 |  | **0.0346** | **0.0216** |
| **9** | **0.0087** | **0.0022** | **0.0022** | 0.0996 | **0.0087** | >0.9999 | 0.3074 | **0.0346** |  | 0.7273 |
| **10** | **0.0065** | **0.0022** | **0.0022** | 0.0606 | **0.0065** | 0.7273 | 0.1970 | **0.0216** | 0.7273 |  |

**Supplementary Table 5. Vocal repertoire of *Fmr1* *+/+, +/- and -/-* females at PND 10**

Comparison of percentage use among different types of USVs within the *+/+* **(A)**, *+/-* **(B)** and *-/-* **(C)** female groups. All p-values are shown in the table, bold when p < 0.05. Mann-Whitney *U* tests. 1= Complex, 2=Downward Ramp, 3= Inverted-U, 4= Upward Ramp, 5= Complex Trill, 6= Short, 7= Step Down, 8= Flat, 9= Step Up, 10=Trill.
