# Supplementary material for: Sex-specific modulation of early life vocalization and cognition by Fmr1 gene dosage in a mouse model of Fragile X Syndrome
Source: Biol Sex Differ. 2024 Feb 21;15:18. doi: 10.1186/s13293-024-00594-3 (PMC10880250; doi:10.1186/s13293-024-00594-3)
Supplement: Supplementary file 6 — Supplementary Material 6: Supplementary table 6. Comparison of vocal repertoire between sexes and genotypes. Comparison of percentage use of different types of USVs by sex and genotype. All p-values are shown in the table, bold when p < 0.05. Mann-Whitney U tests [file 13293_2024_594_MOESM6_ESM.docx]

|  | **Sex** | ***Fmr1*** | **Mean** | **SEM** | **N** | **p-value** | | | | | |
| --- | --- | --- | --- | --- | --- | --- | --- | --- | --- | --- | --- |
|  |  |  |  |  |  | ***+/y*  VS  *-/y*** | ***+/y*  VS  *+/+*** | ***-/y*  VS  *-/-*** | ***+/+*  VS  *+/-*** | ***+/+*  VS  *-/-*** | ***+/-*  VS  *-/-*** |
| Complex | M | *+/y* | 11.255 | 3.752 | 9 | 0.8263 | 0.2972 | 0.4786 | 0.7416 | 0.5338 | 0.5355 |
|  | M | *-/y* | 9.883 | 2.172 | 14 |  |  |  |  |  |  |
|  | F | *+/+* | 17.161 | 3.440 | 7 |  |  |  |  |  |  |
|  | F | *+/-* | 18.114 | 3.131 | 13 |  |  |  |  |  |  |
|  | F | *-/-* | 13.790 | 4.190 | 6 |  |  |  |  |  |  |
| Downw. R. | M | *+/y* | 25.007 | 6.392 | 9 | 0.0645 | 0.4698 | 0.9044 | 0.7137 | 0.4452 | 0.6248 |
|  | M | *-/y* | 34.297 | 3.235 | 14 |  |  |  |  |  |  |
|  | F | *+/+* | 28.452 | 4.235 | 7 |  |  |  |  |  |  |
|  | F | *+/-* | 32.399 | 3.186 | 13 |  |  |  |  |  |  |
|  | F | *-/-* | 38.588 | 9.088 | 6 |  |  |  |  |  |  |
| Inverted-U | M | *+/y* | 7.743 | 3.210 | 9 | 0.5938 | 0.1247 | **0.0424** | 0.3001 | 0.5058 | 0.6853 |
|  | M | *-/y* | 8.426 | 2.009 | 14 |  |  |  |  |  |  |
|  | F | *+/+* | 18.836 | 5.121 | 7 |  |  |  |  |  |  |
|  | F | *+/-* | 14.331 | 3.179 | 13 |  |  |  |  |  |  |
|  | F | *-/-* | 16.420 | 3.227 | 6 |  |  |  |  |  |  |
| Upward R. | M | *+/y* | 10.192 | 5.776 | 9 | 0.7679 | 0.3288 | 0.6994 | >0.9999 | 0.1690 | 0.1677 |
|  | M | *-/y* | 7.676 | 2.045 | 14 |  |  |  |  |  |  |
|  | F | *+/+* | 2.552 | 1.053 | 7 |  |  |  |  |  |  |
|  | F | *+/-* | 2.927 | 0.903 | 13 |  |  |  |  |  |  |
|  | F | *-/-* | 8.001 | 2.738 | 6 |  |  |  |  |  |  |
| Complex Tr. | M | *+/y* | 14.320 | 4.380 | 9 | 0.2431 | 0.6240 | 0.1786 | 0.1765 | 0.8357 | 0.6246 |
|  | M | *-/y* | 18.634 | 2.734 | 14 |  |  |  |  |  |  |
|  | F | *+/+* | 10.198 | 2.556 | 7 |  |  |  |  |  |  |
|  | F | *+/-* | 14.245 | 2.443 | 13 |  |  |  |  |  |  |
|  | F | *-/-* | 12.452 | 3.868 | 6 |  |  |  |  |  |  |
| Short | M | *+/y* | 10.368 | 3.193 | 9 | **0.0220** | 0.0767 | 0.9483 | 0.9298 | 0.9592 | 0.6838 |
|  | M | *-/y* | 1.672 | 0.975 | 14 |  |  |  |  |  |  |
|  | F | *+/+* | 1.395 | 0.805 | 7 |  |  |  |  |  |  |
|  | F | *+/-* | 2.313 | 1.173 | 13 |  |  |  |  |  |  |
|  | F | *-/-* | 1.657 | 1.401 | 6 |  |  |  |  |  |  |
| Step Down | M | *+/y* | 1.393 | 1.035 | 9 | 0.0885 | 0.4808 | >0.9999 | 0.9845 | 0.5804 | 0.4889 |
|  | M | *-/y* | 4.395 | 1.558 | 14 |  |  |  |  |  |  |
|  | F | *+/+* | 2.732 | 1.493 | 7 |  |  |  |  |  |  |
|  | F | *+/-* | 2.203 | 0.847 | 13 |  |  |  |  |  |  |
|  | F | *-/-* | 5.543 | 3.655 | 6 |  |  |  |  |  |  |
| Flat | M | *+/y* | 12.611 | 3.602 | 9 | 0.8289 | 0.8596 | **0.0241** | 0.4495 | 0.1375 | 0.1461 |
|  | M | *-/y* | 9.785 | 1.265 | 14 |  |  |  |  |  |  |
|  | F | *+/+* | 14.287 | 4.359 | 7 |  |  |  |  |  |  |
|  | F | *+/-* | 9.647 | 1.894 | 13 |  |  |  |  |  |  |
|  | F | *-/-* | 5.202 | 1.128 | 6 |  |  |  |  |  |  |
| Step Up | M | *+/y* | 0.926 | 0.926 | 9 | 0.7510 | >0.9999 | 0.1404 | >0.9999 | 0.4371 | 0.6311 |
|  | M | *-/y* | 0.130 | 0.130 | 14 |  |  |  |  |  |  |
|  | F | *+/+* | 0.408 | 0.408 | 7 |  |  |  |  |  |  |
|  | F | *+/-* | 0.347 | 0.203 | 13 |  |  |  |  |  |  |
|  | F | *-/-* | 1.455 | 1.167 | 6 |  |  |  |  |  |  |
| Trill | M | *+/y* | 6.186 | 2.245 | 9 | 0.8124 | 0.5882 | 0.0975 | 0.7453 | 0.2005 | 0.2050 |
|  | M | *-/y* | 5.103 | 1.689 | 14 |  |  |  |  |  |  |
|  | F | *+/+* | 3.981 | 1.711 | 7 |  |  |  |  |  |  |
|  | F | *+/-* | 3.474 | 1.221 | 13 |  |  |  |  |  |  |
|  | F | *-/-* | 1.058 | 1.058 | 6 |  |  |  |  |  |  |

**Supplementary Table 6. Vocal repertoire comparison**

Comparison of percentage use of different types of USVs by sex and genotype. All p-values are shown in the table, bold when p < 0.05. Mann-Whitney *U* tests.
