# Supplementary material for: Sex-specific modulation of early life vocalization and cognition by Fmr1 gene dosage in a mouse model of Fragile X Syndrome
Source: Biol Sex Differ. 2024 Feb 21;15:18. doi: 10.1186/s13293-024-00594-3 (PMC10880250; doi:10.1186/s13293-024-00594-3)
Supplement: Supplementary file 7 — Supplementary Material 7: Supplementary table 7. Transition probability from different USVs in males. Comparison among transition probabilities from different types of USVs within the +/y (A) and -/y (B) male groups. All p-values are shown in the table, bold when p < 0.05. Mann-Whitney U tests. 1 = Complex, 2 = Downward Ramp, 3 = Inverted-U, 4 = Upward Ramp, 5 = Complex Trill, 6 = Short, 7 = Step Down, 8 = Flat, 9 = Step Up, 10 = Trill [file 13293_2024_594_MOESM7_ESM.docx]

| **A** | **1** | **2** | **3** | **4** | **5** | **6** | **7** | **8** | **9** | **10** |
| --- | --- | --- | --- | --- | --- | --- | --- | --- | --- | --- |
| **1** |  | 0.2809 | 0.5776 | 0.1374 | 0.6663 | 0.6571 | **0.0226** | 0.5784 | **0.0226** | 0.1811 |
| **2** | 0.2809 |  | 0.1499 | **0.0226** | 0.2078 | 0.7367 | **0.0041** | 0.2202 | **0.0041** | **0.0228** |
| **3** | 0.5776 | 0.1499 |  | 0.2670 | 0.9084 | 0.5335 | 0.0905 | 0.7149 | 0.0905 | 0.3953 |
| **4** | 0.1374 | **0.0226** | 0.2670 |  | 0.2805 | 0.0769 | 0.4706 | 0.4353 | 0.4706 | >0.999 |
| **5** | 0.6663 | 0.2078 | 0.9084 | 0.2805 |  | 0.4615 | 0.0701 | 0.7547 | 0.0701 | 0.3348 |
| **6** | 0.6571 | 0.7367 | 0.5335 | 0.0769 | 0.4615 |  | **0.0294** | 0.4817 | **0.0294** | 0.1275 |
| **7** | **0.0226** | **0.0041** | 0.0905 | 0.4706 | 0.0701 | **0.0294** |  | 0.2059 | >0.9999 | 0.3294 |
| **8** | 0.5784 | 0.2202 | 0.7149 | 0.4353 | 0.7547 | 0.4817 | 0.2059 |  | 0.2059 | 0.6579 |
| **9** | **0.0226** | **0.0041** | 0.0905 | 0.4706 | 0.0701 | **0.0294** | >0.9999 | 0.2059 |  | 0.3294 |
| **10** | 0.1811 | **0.0228** | 0.3953 | >0.9999 | 0.3348 | 0.1275 | 0.3294 | 0.6579 | 0.3294 |  |
|  |  |  |  |  |  |  |  |  |  |  |
| **B** | **1** | **2** | **3** | **4** | **5** | **6** | **7** | **8** | **9** | **10** |
| **1** |  | **<0.0001** | 0.513 | 0.8449 | **0.0307** | **0.0014** | 0.308 | 0.972 | **0.0014** | 0.6623 |
| **2** | **<0.0001** |  | **<0.0001** | **<0.0001** | **0.0055** | **<0.0001** | **<0.0001** | **<0.0001** | **<0.0001** | **<0.0001** |
| **3** | 0.513 | **<0.0001** |  | 0.3514 | 0.0594 | **0.0001** | 0.0834 | 0.3544 | **<0.0001** | 0.3952 |
| **4** | 0.8449 | **<0.0001** | 0.3514 |  | **0.0223** | **0.0019** | 0.2963 | 0.8457 | **0.0019** | 0.6968 |
| **5** | **0.0307** | **0.0055** | 0.0594 | **0.0223** |  | **<0.0001** | **0.0023** | **0.0046** | **<0.0001** | **0.0292** |
| **6** | **0.0014** | **<0.0001** | **0.0001** | **0.0019** | **<0.0001** |  | **0.0464** | **0.0001** | >0.9999 | **0.0135** |
| **7** | 0.308 | **<0.0001** | 0.0834 | 0.2963 | **0.0023** | **0.0464** |  | 0.1244 | **0.0464** | 0.5049 |
| **8** | 0.972 | **<0.0001** | 0.3544 | 0.8457 | **0.0046** | **0.0001** | 0.1244 |  | **<0.0001** | 0.7192 |
| **9** | **0.0014** | **<0.0001** | **<0.0001** | **0.0019** | **<0.0001** | >0.9999 | **0.0464** | **<0.0001** |  | **0.0097** |
| **10** | 0.6623 | **<0.0001** | 0.3952 | 0.6968 | **0.0292** | **0.0135** | 0.5049 | 0.7192 | **0.0097** |  |

**Supplementary Table 7. Transition probability from different USVs in males at PND 10**

Comparison among transition probabilities from different types of USVs within the *+/y* **(A)** and *-/y* **(B)** male groups. All p-values are shown in the table, bold when p < 0.05. Mann-Whitney *U* tests. 1= Complex, 2=Downward Ramp, 3= Inverted-U, 4= Upward Ramp, 5= Complex Trill, 6= Short, 7= Step Down, 8= Flat, 9= Step Up, 10=Trill.
