# Supplementary material for: Sex-specific modulation of early life vocalization and cognition by Fmr1 gene dosage in a mouse model of Fragile X Syndrome
Source: Biol Sex Differ. 2024 Feb 21;15:18. doi: 10.1186/s13293-024-00594-3 (PMC10880250; doi:10.1186/s13293-024-00594-3)
Supplement: Supplementary file 8 — Supplementary Material 8: Supplementary table 8. Transition probability from different USVs in females. Comparison among the transition probabilities from different types of USVs within the +/+ (A), +/- (B) and -/- (C) female groups. All p-values are shown in the table, bold when p < 0.05. Mann-Whitney U tests. 1 = Complex, 2 = Downward Ramp, 3 = Inverted-U, 4 = Upward Ramp, 5 = Complex Trill, 6 = Short, 7 = Step Down, 8 = Flat, 9 = Step Up, 10 = Trill [file 13293_2024_594_MOESM8_ESM.docx]

| **A** | **1** | **2** | **3** | **4** | **5** | **6** | **7** | **8** | **9** | **10** |
| --- | --- | --- | --- | --- | --- | --- | --- | --- | --- | --- |
| **1** |  | 0.0810 | 0.2920 | 0.1253 | 0.1958 | 0.0781 | 0.0938 | 0.2931 | **0.0210** | 0.3036 |
| **2** | 0.0810 |  | 0.5921 | **0.0029** | **0.0070** | **0.0012** | **0.0017** | 0.5157 | **0.0006** | **0.0082** |
| **3** | 0.2920 | 0.5921 |  | 0.0746 | 0.1661 | 0.0554 | 0.0746 | 0.5070 | **0.0210** | 0.1661 |
| **4** | 0.1253 | **0.0029** | 0.0746 |  | 0.4371 | 0.7552 | 0.9184 | 0.0973 | 0.1923 | 0.4371 |
| **5** | 0.1958 | **0.0070** | 0.1661 | 0.4371 |  | 0.3432 | 0.4371 | 0.1841 | 0.1189 | 0.8776 |
| **6** | 0.0781 | **0.0012** | 0.0554 | 0.7552 | 0.3432 |  | 0.8776 | 0.0816 | 0.4371 | 0.2780 |
| **7** | 0.0938 | **0.0017** | 0.0746 | 0.9184 | 0.4371 | 0.8776 |  | 0.0816 | 0.3147 | 0.3759 |
| **8** | 0.2931 | 0.5157 | 0.5070 | 0.0973 | 0.1841 | 0.0816 | 0.0816 |  | **0.0210** | 0.1841 |
| **9** | **0.0210** | **0.0006** | **0.0210** | 0.1923 | 0.1189 | 0.4371 | 0.3147 | **0.0210** |  | 0.0699 |
| **10** | 0.3036 | **0.0082** | 0.1661 | 0.4371 | 0.8776 | 0.2780 | 0.3759 | 0.1841 | 0.0699 |  |
|  |  |  |  |  |  |  |  |  |  |  |
| **B** | **1** | **2** | **3** | **4** | **5** | **6** | **7** | **8** | **9** | **10** |
| **1** |  | 0.3814 | 0.2405 | **0.0071** | 0.6571 | **0.0071** | **0.0024** | **0.0480** | **0.0001** | **0.0252** |
| **2** | 0.3814 |  | **0.0442** | **<0.0001** | 0.1282 | **<0.0001** | **<0.0001** | **0.0008** | **<0.0001** | **0.0013** |
| **3** | 0.2405 | **0.0442** |  | 0.0853 | 0.5127 | 0.0853 | **0.0463** | 0.3999 | **0.0016** | 0.2401 |
| **4** | **0.0071** | **<0.0001** | 0.0853 |  | **0.0071** | 0.9929 | 0.7822 | 0.1518 | **0.0440** | 0.4399 |
| **5** | 0.6571 | 0.1282 | 0.5127 | **0.0071** |  | **0.0085** | **0.0044** | 0.1008 | **0.0001** | 0.0661 |
| **6** | **0.0071** | **<0.0001** | 0.0853 | 0.9929 | **0.0085** |  | 0.8367 | 0.1231 | **0.0440** | 0.3781 |
| **7** | **0.0024** | **<0.0001** | **0.0463** | 0.7822 | **0.0044** | 0.8367 |  | **0.0499** | 0.1118 | 0.3124 |
| **8** | **0.0480** | **0.0008** | 0.3999 | 0.1518 | 0.1008 | 0.1231 | **0.0499** |  | **0.0015** | 0.4910 |
| **9** | **0.0001** | **<0.0001** | **0.0016** | **0.0440** | **0.0001** | **0.0440** | 0.1118 | **0.0015** |  | **0.0124** |
| **10** | **0.0252** | **0.0013** | 0.2401 | 0.4399 | 0.0661 | 0.3781 | 0.3124 | 0.4910 | **0.0124** |  |
|  |  |  |  |  |  |  |  |  |  |  |
| **C** | **1** | **2** | **3** | **4** | **5** | **6** | **7** | **8** | **9** | **10** |
| **1** |  | 0.3095 | 0.6991 | 0.2251 | 0.7381 | **0.0065** | **0.0390** | **0.0065** | **0.0043** | **0.0087** |
| **2** | 0.3095 |  | 0.1320 | 0.0887 | 0.1797 | **0.0043** | **0.0260** | **0.0043** | **0.0043** | **0.0043** |
| **3** | 0.6991 | 0.1320 |  | 0.3268 | 0.9372 | **0.0065** | 0.1688 | **0.0087** | **0.0108** | **0.0087** |
| **4** | 0.2251 | 0.0887 | 0.3268 |  | 0.4545 | 0.1515 | 0.6710 | 0.2208 | 0.1775 | 0.1970 |
| **5** | 0.7381 | 0.1797 | 0.9372 | 0.4545 |  | **0.0065** | 0.0628 | **0.0108** | **0.0087** | **0.0216** |
| **6** | **0.0065** | **0.0043** | **0.0065** | 0.1515 | **0.0065** |  | 0.1970 | 0.5455 | >0.9999 | >0.9999 |
| **7** | **0.0390** | **0.0260** | 0.1688 | 0.6710 | 0.0628 | 0.1970 |  | 0.513 | 0.3723 | 0.1970 |
| **8** | **0.0065** | **0.0043** | **0.0087** | 0.2208 | **0.0108** | 0.5455 | 0.5130 |  | 0.8485 | 0.5455 |
| **9** | **0.0043** | **0.0043** | **0.0108** | 0.1775 | **0.0087** | >0.9999 | 0.3723 | 0.8485 |  | >0.9999 |
| **10** | **0.0087** | **0.0043** | **0.0087** | 0.1970 | **0.0216** | >0.9999 | 0.1970 | 0.5455 | >0.9999 |  |

**Supplementary Table 8. Transition probability from different USVs in females at PND 10**

Comparison among the transition probabilities from different types of USVs within the *+/+* **(A)**, *+/-* **(B)** and *-/-* **(C)** female groups. All p-values are shown in the table, bold when p < 0.05. Mann-Whitney *U* tests. 1= Complex, 2=Downward Ramp, 3= Inverted-U, 4= Upward Ramp, 5= Complex Trill, 6= Short, 7= Step Down, 8= Flat, 9= Step Up, 10=Trill.
