# Supplementary material for: Sex-specific modulation of early life vocalization and cognition by Fmr1 gene dosage in a mouse model of Fragile X Syndrome
Source: Biol Sex Differ. 2024 Feb 21;15:18. doi: 10.1186/s13293-024-00594-3 (PMC10880250; doi:10.1186/s13293-024-00594-3)
Supplement: Supplementary file 9 — Supplementary Material 9: Supplementary Table 9. Comparison of transition probability from different USVs. Comparison transition probabilities from different types of USVs by sex and genotype. All p-values are shown in the table, bold when p < 0.05. Mann-Whitney U tests [file 13293_2024_594_MOESM9_ESM.docx]

|  | **Sex** | ***Fmr1*** | **Mean** | **SEM** | **N** | **p-value** | | | | | |
| --- | --- | --- | --- | --- | --- | --- | --- | --- | --- | --- | --- |
|  |  |  |  |  |  | ***+/y*  VS  *-/y*** | ***+/y*  VS  *+/+*** | ***-/y*  VS  *-/-*** | ***+/+*  VS  *+/-*** | ***+/+*  VS  *-/-*** | ***+/-*  VS  *-/-*** |
| Complex | M | *+/y* | 0.0427 | 0.0181 | 9 | 0.8371 | 0.6196 | 0.0571 | 0.1117 | 0.3141 | 0.8309 |
|  | M | *-/y* | 0.0361 | 0.0123 | 14 |  |  |  |  |  |  |
|  | F | *+/+* | 0.0421 | 0.0136 | 7 |  |  |  |  |  |  |
|  | F | *+/-* | 0.0968 | 0.0237 | 13 |  |  |  |  |  |  |
|  | F | *-/-* | 0.0827 | 0.0262 | 6 |  |  |  |  |  |  |
| Downw. R. | M | *+/y* | 0.0661 | 0.0182 | 9 | **0.0014** | 0.2177 | 0.3010 | 0.8165 | 0.9452 | 0.9494 |
|  | M | *-/y* | 0.1615 | 0.0158 | 14 |  |  |  |  |  |  |
|  | F | *+/+* | 0.1125 | 0.0290 | 7 |  |  |  |  |  |  |
|  | F | *+/-* | 0.1182 | 0.0183 | 13 |  |  |  |  |  |  |
|  | F | *-/-* | 0.1327 | 0.0442 | 6 |  |  |  |  |  |  |
| Inverted-U | M | *+/y* | 0.0310 | 0.0138 | 9 | 0.5388 | 0.1231 | 0.1328 | 0.4153 | 0.5536 | 0.7475 |
|  | M | *-/y* | 0.0379 | 0.0080 | 14 |  |  |  |  |  |  |
|  | F | *+/+* | 0.1099 | 0.0456 | 7 |  |  |  |  |  |  |
|  | F | *+/-* | 0.0601 | 0.0180 | 13 |  |  |  |  |  |  |
|  | F | *-/-* | 0.0538 | 0.0124 | 6 |  |  |  |  |  |  |
| Upward R. | M | *+/y* | 0.0156 | 0.0108 | 9 | 0.1311 | 0.6769 | 0.6379 | 0.9447 | 0.2232 | 0.1837 |
|  | M | *-/y* | 0.0297 | 0.0092 | 14 |  |  |  |  |  |  |
|  | F | *+/+* | 0.0111 | 0.0054 | 7 |  |  |  |  |  |  |
|  | F | *+/-* | 0.0136 | 0.0079 | 13 |  |  |  |  |  |  |
|  | F | *-/-* | 0.0430 | 0.0218 | 6 |  |  |  |  |  |  |
| Complex Tr. | M | *+/y* | 0.0352 | 0.0169 | 9 | **0.0418** | 0.8568 | 0.5064 | 0.0550 | 0.0501 | 0.7804 |
|  | M | *-/y* | 0.0909 | 0.0215 | 14 |  |  |  |  |  |  |
|  | F | *+/+* | 0.0197 | 0.0084 | 7 |  |  |  |  |  |  |
|  | F | *+/-* | 0.0788 | 0.0200 | 13 |  |  |  |  |  |  |
|  | F | *-/-* | 0.0632 | 0.0183 | 6 |  |  |  |  |  |  |
| Short | M | *+/y* | 0.0600 | 0.0209 | 9 | **0.0037** | 0.2107 | 0.7789 | 0.9298 | 0.5594 | 0.3315 |
|  | M | *-/y* | 0.0018 | 0.0018 | 14 |  |  |  |  |  |  |
|  | F | *+/+* | 0.0071 | 0.0045 | 7 |  |  |  |  |  |  |
|  | F | *+/-* | 0.0141 | 0.0081 | 13 |  |  |  |  |  |  |
|  | F | *-/-* | 0.0069 | 0.0069 | 6 |  |  |  |  |  |  |
| Step Down | M | *+/y* | 0.0037 | 0.0037 | 9 | 0.1341 | 0.2615 | 0.5137 | 0.7880 | 0.4901 | 0.2811 |
|  | M | *-/y* | 0.0185 | 0.0082 | 14 |  |  |  |  |  |  |
|  | F | *+/+* | 0.0092 | 0.0050 | 7 |  |  |  |  |  |  |
|  | F | *+/-* | 0.0100 | 0.0057 | 13 |  |  |  |  |  |  |
|  | F | *-/-* | 0.0245 | 0.0156 | 6 |  |  |  |  |  |  |
| Flat | M | *+/y* | 0.0444 | 0.0255 | 9 | 0.4616 | 0.1997 | 0.0616 | 0.1696 | 0.1183 | 0.2312 |
|  | M | *-/y* | 0.0257 | 0.0044 | 14 |  |  |  |  |  |  |
|  | F | *+/+* | 0.0771 | 0.0288 | 7 |  |  |  |  |  |  |
|  | F | *+/-* | 0.0312 | 0.0097 | 13 |  |  |  |  |  |  |
|  | F | *-/-* | 0.0094 | 0.0057 | 6 |  |  |  |  |  |  |
| Step Up | M | *+/y* | 0.0037 | 0.0037 | 9 | 0.7510 | >0.9999 | 0.1404 | >0.9999 | 0.4371 | 0.1548 |
|  | M | *-/y* | 0.0014 | 0.0014 | 14 |  |  |  |  |  |  |
|  | F | *+/+* | 0.0024 | 0.0024 | 7 |  |  |  |  |  |  |
|  | F | *+/-* | 0.0015 | 0.0015 | 13 |  |  |  |  |  |  |
|  | F | *-/-* | 0.0083 | 0.0057 | 6 |  |  |  |  |  |  |
| Trill | M | *+/y* | 0.0133 | 0.0073 | 9 | 0.3462 | 0.4199 | 0.2400 | >0.9999 | 0.3310 | 0.2674 |
|  | M | *-/y* | 0.0310 | 0.0110 | 14 |  |  |  |  |  |  |
|  | F | *+/+* | 0.0232 | 0.0096 | 7 |  |  |  |  |  |  |
|  | F | *+/-* | 0.0300 | 0.0142 | 13 |  |  |  |  |  |  |
|  | F | *-/-* | 0.0113 | 0.0113 | 6 |  |  |  |  |  |  |

**Supplementary Table 9.** **Comparison of transition probability from different USVs**

Comparison transition probabilities from different types of USVs by sex and genotype. All p-values are shown in the table, bold when p < 0.05. Mann-Whitney *U* tests.
