# Supplementary material for: Sex-specific modulation of early life vocalization and cognition by Fmr1 gene dosage in a mouse model of Fragile X Syndrome
Source: Biol Sex Differ. 2024 Feb 21;15:18. doi: 10.1186/s13293-024-00594-3 (PMC10880250; doi:10.1186/s13293-024-00594-3)
Supplement: Supplementary file 10 — Supplementary Material 10: Supplementary table 10. Transition probability to different USVs in males. Comparison among transition probabilities to different types of USVs within the +/y (A) and -/y (B) male groups. All p-values are shown in the table, bold when p < 0.05. Mann-Whitney U tests. 1 = Complex, 2 = Downward Ramp, 3 = Inverted-U, 4 = Upward Ramp, 5 = Complex Trill, 6 = Short, 7 = Step Down, 8 = Flat, 9 = Step Up, 10 = Trill [file 13293_2024_594_MOESM10_ESM.docx]

| **A** | **1** | **2** | **3** | **4** | **5** | **6** | **7** | **8** | **9** | **10** |
| --- | --- | --- | --- | --- | --- | --- | --- | --- | --- | --- |
| **1** |  | 0.7708 | 0.4731 | 0.2657 | 0.4811 | 0.3694 | 0.1621 | 0.8200 | **0.0498** | 0.2657 |
| **2** | 0.7708 |  | 0.2798 | 0.0933 | 0.2869 | 0.2626 | 0.0797 | 0.6133 | **0.0049** | 0.1131 |
| **3** | 0.4731 | 0.2798 |  | 0.6389 | 0.9067 | 0.9240 | 0.6199 | 0.7149 | 0.2941 | 0.6959 |
| **4** | 0.2657 | 0.0933 | 0.6389 |  | 0.7149 | 0.7719 | >0.9999 | 0.3608 | 0.5765 | >0.9999 |
| **5** | 0.4811 | 0.2869 | 0.9067 | 0.7149 |  | >0.9999 | 0.6199 | 0.5249 | 0.2941 | 0.7910 |
| **6** | 0.3694 | 0.2626 | 0.9240 | 0.7719 | >0.9999 |  | >0.9999 | 0.5508 | 0.5765 | 0.7719 |
| **7** | 0.1621 | 0.0797 | 0.6199 | >0.9999 | 0.6199 | >0.9999 |  | 0.3348 | >0.9999 | >0.9999 |
| **8** | 0.8200 | 0.6133 | 0.7149 | 0.3608 | 0.5249 | 0.5508 | 0.3348 |  | 0.1312 | 0.3608 |
| **9** | **0.0498** | **0.0049** | 0.2941 | 0.5765 | 0.2941 | 0.5765 | >0.9999 | 0.1312 |  | 0.5765 |
| **10** | 0.2657 | 0.1131 | 0.6959 | >0.9999 | 0.7910 | 0.7719 | >0.9999 | 0.3608 | 0.5765 |  |
|  |  |  |  |  |  |  |  |  |  |  |
| **B** | **1** | **2** | **3** | **4** | **5** | **6** | **7** | **8** | **9** | **10** |
| **1** |  | 0.6576 | 0.6684 | 0.5359 | 0.2187 | **0.0155** | 0.3202 | 0.5398 | **0.0002** | 0.1743 |
| **2** | 0.6576 |  | 0.7940 | 0.5150 | 0.2221 | **0.0009** | 0.1481 | 0.4566 | **<0.0001** | 0.0634 |
| **3** | 0.6684 | 0.7940 |  | 0.8435 | 0.4860 | **0.0238** | 0.4697 | 0.2779 | **0.0002** | 0.2660 |
| **4** | 0.5359 | 0.5150 | 0.8435 |  | 0.9186 | **0.0425** | 0.5419 | 0.2508 | **0.0006** | 0.3836 |
| **5** | 0.2187 | 0.2221 | 0.4860 | 0.9186 |  | **0.0092** | 0.4803 | 0.0801 | **<0.0001** | 0.2873 |
| **6** | **0.0155** | **0.0009** | **0.0238** | **0.0425** | **0.0092** |  | 0.1809 | **0.0018** | **0.2222** | 0.3067 |
| **7** | 0.3202 | 0.1481 | 0.4697 | 0.5419 | 0.4803 | 0.1809 |  | 0.0869 | **0.0058** | 0.7747 |
| **8** | 0.5398 | 0.4566 | 0.2779 | 0.2508 | 0.0801 | **0.0018** | 0.0869 |  | **<0.0001** | **0.0422** |
| **9** | **0.0002** | **<0.0001** | **0.0002** | **0.0006** | **<0.0001** | **0.2222** | **0.0058** | **<0.0001** |  | **0.0159** |
| **10** | 0.1743 | 0.0634 | 0.2660 | 0.3836 | 0.2873 | 0.3067 | 0.7747 | **0.0422** | **0.0159** |  |

**Supplementary Table 10. Transition probability to different USVs in males at PND 10**

Comparison among transition probabilities to different types of USVs within the *+/y* **(A)** and *-/y* **(B)** male groups. All p-values are shown in the table, bold when p < 0.05. Mann-Whitney *U* tests. 1= Complex, 2=Downward Ramp, 3= Inverted-U, 4= Upward Ramp, 5= Complex Trill, 6= Short, 7= Step Down, 8= Flat, 9= Step Up, 10=Trill.
