# Supplementary material for: Sex-specific modulation of early life vocalization and cognition by Fmr1 gene dosage in a mouse model of Fragile X Syndrome
Source: Biol Sex Differ. 2024 Feb 21;15:18. doi: 10.1186/s13293-024-00594-3 (PMC10880250; doi:10.1186/s13293-024-00594-3)
Supplement: Supplementary file 11 — Supplementary Material 11: Supplementary table 11. Transition probability to different USVs in females. Comparison among the transition probabilities to different types of USVs within the +/+ (A), +/- (B) and -/- (C) female groups. All p-values are shown in the table, bold when p < 0.05. Mann-Whitney U tests. 1 = Complex, 2 = Downward Ramp, 3 = Inverted-U, 4 = Upward Ramp, 5 = Complex Trill, 6 = Short, 7 = Step Down, 8 = Flat, 9 = Step Up, 10 = Trill [file 13293_2024_594_MOESM11_ESM.docx]

| **A** | **1** | **2** | **3** | **4** | **5** | **6** | **7** | **8** | **9** | **10** |
| --- | --- | --- | --- | --- | --- | --- | --- | --- | --- | --- |
| **1** |  | 0.7337 | 0.6853 | 0.8485 | 0.7354 | 0.3648 | 0.1725 | 0.3147 | **0.0047** | 0.1638 |
| **2** | 0.7337 |  | >0.9999 | 0.8904 | 0.9027 | 0.3648 | 0.2337 | 0.3916 | **0.0047** | 0.2045 |
| **3** | 0.6853 | >0.9999 |  | >0.9999 | 0.9417 | 0.4872 | 0.4289 | 0.5047 | **0.0210** | 0.2477 |
| **4** | 0.8485 | 0.8904 | >0.9999 |  | >0.9999 | 0.7960 | 0.5921 | 0.7086 | 0.0699 | 0.4697 |
| **5** | 0.7354 | 0.9027 | 0.9417 | >0.9999 |  | 0.5629 | 0.3065 | 0.6125 | **0.0210** | 0.3328 |
| **6** | 0.3648 | 0.3648 | 0.4872 | 0.7960 | 0.5629 |  | >0.9999 | 0.8776 | 0.1923 | 0.8776 |
| **7** | 0.1725 | 0.2337 | 0.4289 | 0.5921 | 0.3065 | >0.9999 |  | 0.6329 | 0.4615 | >0.9999 |
| **8** | 0.3147 | 0.3916 | 0.5047 | 0.7086 | 0.6125 | 0.8776 | 0.6329 |  | 0.0699 | 0.6941 |
| **9** | **0.0047** | **0.0047** | **0.0210** | 0.0699 | **0.0210** | 0.1923 | 0.4615 | 0.0699 |  | 0.1923 |
| **10** | 0.1638 | 0.2045 | 0.2477 | 0.4697 | 0.3328 | 0.8776 | >0.9999 | 0.6941 | 0.1923 |  |
|  |  |  |  |  |  |  |  |  |  |  |
| **B** | **1** | **2** | **3** | **4** | **5** | **6** | **7** | **8** | **9** | **10** |
| **1** |  | 0.5503 | 0.4791 | 0.8241 | 0.3382 | **0.0056** | **0.0249** | 0.4027 | **0.0125** | 0.7842 |
| **2** | 0.5503 |  | 0.9086 | 0.8052 | 0.0651 | **0.0003** | **0.0012** | 0.1061 | **0.0018** | 0.8447 |
| **3** | 0.4791 | 0.9086 |  | 0.7925 | 0.1756 | **0.0063** | **0.0139** | 0.1963 | **0.0066** | 0.6741 |
| **4** | 0.8241 | 0.8052 | 0.7925 |  | 0.6127 | 0.0676 | 0.1390 | 0.5140 | 0.0802 | 0.8497 |
| **5** | 0.3382 | 0.0651 | 0.1756 | 0.6127 |  | **0.0100** | 0.0634 | 0.9081 | **0.0148** | 0.5756 |
| **6** | **0.0056** | **0.0003** | **0.0063** | 0.0676 | **0.0100** |  | 0.3951 | **0.0431** | 0.8643 | 0.0876 |
| **7** | **0.0249** | **0.0012** | **0.0139** | 0.1390 | 0.0634 | 0.3951 |  | 0.1786 | 0.3783 | 0.1523 |
| **8** | 0.4027 | 0.1061 | 0.1963 | 0.5140 | 0.9081 | **0.0431** | 0.1786 |  | 0.0537 | 0.5447 |
| **9** | **0.0125** | **0.0018** | **0.0066** | 0.0802 | **0.0148** | 0.8643 | 0.3783 | 0.0537 |  | 0.0968 |
| **10** | 0.7842 | 0.8447 | 0.6741 | 0.8497 | 0.5756 | 0.0876 | 0.1523 | 0.5447 | 0.0968 |  |
|  |  |  |  |  |  |  |  |  |  |  |
| **C** | **1** | **2** | **3** | **4** | **5** | **6** | **7** | **8** | **9** | **10** |
| **1** |  | 0.8550 | 0.8485 | 0.7532 | 0.9069 | 0.1515 | 0.5671 | 0.2576 | 0.3074 | 0.2424 |
| **2** | 0.8550 |  | 0.7835 | >0.9999 | 0.4632 | **0.0087** | 0.1234 | 0.3636 | 0.0779 | **0.0476** |
| **3** | 0.8485 | 0.7835 |  | 0.8463 | 0.5108 | **0.0087** | 0.1407 | 0.4935 | 0.1082 | **0.0368** |
| **4** | 0.7532 | >0.9999 | 0.8463 |  | 0.9004 | 0.1515 | 0.4264 | 0.3680 | 0.3398 | 0.2424 |
| **5** | 0.9069 | 0.4632 | 0.5108 | 0.9004 |  | 0.0671 | 0.3312 | 0.2619 | 0.2100 | 0.1126 |
| **6** | 0.1515 | **0.0087** | **0.0087** | 0.1515 | 0.0671 |  | 0.4242 | **0.0411** | 0.7273 | >0.9999 |
| **7** | 0.5671 | 0.1234 | 0.1407 | 0.4264 | 0.3312 | 0.4242 |  | 0.1169 | 0.7727 | 0.5455 |
| **8** | 0.2576 | 0.3636 | 0.4935 | 0.3680 | 0.2619 | **0.0411** | 0.1169 |  | 0.0909 | 0.0801 |
| **9** | 0.3074 | 0.0779 | 0.1082 | 0.3398 | 0.2100 | 0.7273 | 0.7727 | 0.0909 |  | >0.9999 |
| **10** | 0.2424 | **0.0476** | **0.0368** | 0.2424 | 0.1126 | >0.9999 | 0.5455 | 0.0801 | >0.9999 |  |

**Supplementary Table 11. Transition probability to different USVs in females at PND 10**

Comparison among the transition probabilities to different types of USVs within the *+/+* **(A)**, *+/-* **(B)** and *-/-* **(C)** female groups. All p-values are shown in the table, bold when p < 0.05. Mann-Whitney *U* tests. 1= Complex, 2=Downward Ramp, 3= Inverted-U, 4= Upward Ramp, 5= Complex Trill, 6= Short, 7= Step Down, 8= Flat, 9= Step Up, 10=Trill.
