# Supplementary material for: Sex-specific modulation of early life vocalization and cognition by Fmr1 gene dosage in a mouse model of Fragile X Syndrome
Source: Biol Sex Differ. 2024 Feb 21;15:18. doi: 10.1186/s13293-024-00594-3 (PMC10880250; doi:10.1186/s13293-024-00594-3)
Supplement: Supplementary file 12 — Supplementary Material 12: Supplementary Table 12. Comparison of transition probability to different USVs. Comparison transition probabilities to different types of USVs by sex and genotype. All p-values are shown in the table. Mann-Whitney U tests [file 13293_2024_594_MOESM12_ESM.docx]

|  | **Sex** | ***Fmr1*** | **Mean** | **SEM** | **N** | **p-value** | | | | | |
| --- | --- | --- | --- | --- | --- | --- | --- | --- | --- | --- | --- |
|  |  |  |  |  |  | ***+/y*  VS  *-/y*** | ***+/y*  VS  *+/+*** | ***-/y*  VS  *-/-*** | ***+/+*  VS  *+/-*** | ***+/+*  VS  *-/-*** | ***+/-*  VS  *-/-*** |
| Complex | M | *+/y* | 0.0470 | 0.0146 | 9 | 0.5844 | 0.7229 | 0.5234 | 0.8900 | 0.7063 | 0.7465 |
|  | M | *-/y* | 0.0602 | 0.0111 | 14 |  |  |  |  |  |  |
|  | F | *+/+* | 0.0614 | 0.0155 | 7 |  |  |  |  |  |  |
|  | F | *+/-* | 0.0604 | 0.0105 | 13 |  |  |  |  |  |  |
|  | F | *-/-* | 0.0503 | 0.0174 | 6 |  |  |  |  |  |  |
| Downw. R. | M | *+/y* | 0.0509 | 0.0095 | 9 | 0.2384 | 0.5184 | 0.8251 | 0.4249 | 0.7010 | 0.1499 |
|  | M | *-/y* | 0.0625 | 0.0064 | 14 |  |  |  |  |  |  |
|  | F | *+/+* | 0.0588 | 0.0138 | 7 |  |  |  |  |  |  |
|  | F | *+/-* | 0.0729 | 0.0075 | 13 |  |  |  |  |  |  |
|  | F | *-/-* | 0.0635 | 0.0051 | 6 |  |  |  |  |  |  |
| Inverted-U | M | *+/y* | 0.0333 | 0.0147 | 9 | 0.3261 | 0.3808 | 0.6974 | 0.4891 | 0.9732 | 0.6852 |
|  | M | *-/y* | 0.0533 | 0.0109 | 14 |  |  |  |  |  |  |
|  | F | *+/+* | 0.0582 | 0.0158 | 7 |  |  |  |  |  |  |
|  | F | *+/-* | 0.0655 | 0.0116 | 13 |  |  |  |  |  |  |
|  | F | *-/-* | 0.0657 | 0.0103 | 6 |  |  |  |  |  |  |
| Upward R. | M | *+/y* | 0.0233 | 0.0125 | 9 | 0.1502 | 0.3428 | 0.9523 | 0.9393 | >0.9999 | 0.7175 |
|  | M | *-/y* | 0.0499 | 0.0114 | 14 |  |  |  |  |  |  |
|  | F | *+/+* | 0.0500 | 0.0189 | 7 |  |  |  |  |  |  |
|  | F | *+/-* | 0.0524 | 0.0141 | 13 |  |  |  |  |  |  |
|  | F | *-/-* | 0.0518 | 0.0177 | 6 |  |  |  |  |  |  |
| Complex Tr. | M | *+/y* | 0.0302 | 0.0128 | 9 | 0.1575 | 0.3453 | 0.9033 | 0.7402 | 0.9044 | 0.9853 |
|  | M | *-/y* | 0.0485 | 0.0074 | 14 |  |  |  |  |  |  |
|  | F | *+/+* | 0.0524 | 0.0164 | 7 |  |  |  |  |  |  |
|  | F | *+/-* | 0.0492 | 0.0095 | 13 |  |  |  |  |  |  |
|  | F | *-/-* | 0.0502 | 0.0123 | 6 |  |  |  |  |  |  |
| Short | M | *+/y* | 0.0311 | 0.0157 | 9 | 0.5154 | 0.9266 | 0.7746 | 0.4318 | 0.4126 | 0.8893 |
|  | M | *-/y* | 0.0179 | 0.0100 | 14 |  |  |  |  |  |  |
|  | F | *+/+* | 0.0357 | 0.0180 | 7 |  |  |  |  |  |  |
|  | F | *+/-* | 0.0145 | 0.0087 | 13 |  |  |  |  |  |  |
|  | F | *-/-* | 0.0111 | 0.0111 | 6 |  |  |  |  |  |  |
| Step Down | M | *+/y* | 0.0222 | 0.0147 | 9 | 0.3679 | >0.9999 | 0.7968 | >0.9999 | 0.7552 | 0.7872 |
|  | M | *-/y* | 0.0393 | 0.0121 | 14 |  |  |  |  |  |  |
|  | F | *+/+* | 0.0286 | 0.0184 | 7 |  |  |  |  |  |  |
|  | F | *+/-* | 0.0251 | 0.0098 | 13 |  |  |  |  |  |  |
|  | F | *-/-* | 0.0304 | 0.0167 | 6 |  |  |  |  |  |  |
| Flat | M | *+/y* | 0.0422 | 0.0158 | 9 | 0.2005 | 0.8948 | 0.9059 | 0.7315 | 0.1830 | 0.2002 |
|  | M | *-/y* | 0.0685 | 0.0095 | 14 |  |  |  |  |  |  |
|  | F | *+/+* | 0.0395 | 0.0160 | 7 |  |  |  |  |  |  |
|  | F | *+/-* | 0.0464 | 0.0115 | 13 |  |  |  |  |  |  |
|  | F | *-/-* | 0.0711 | 0.0160 | 6 |  |  |  |  |  |  |
| Step Up | M | *+/y* | 0.0111 | 0.0111 | 9 | 0.3913 | >0.9999 | 0.0789 | 0.5211 | 0.1923 | 0.5573 |
|  | M | *-/y* | 0.0000 | 0.0000 | 14 |  |  |  |  |  |  |
|  | F | *+/+* | 0.0000 | 0.0000 | 7 |  |  |  |  |  |  |
|  | F | *+/-* | 0.0154 | 0.0104 | 13 |  |  |  |  |  |  |
|  | F | *-/-* | 0.0250 | 0.0171 | 6 |  |  |  |  |  |  |
| Trill | M | *+/y* | 0.0241 | 0.0128 | 9 | 0.6166 | 0.7871 | 0.4724 | 0.3706 | 0.5594 | 0.2885 |
|  | M | *-/y* | 0.0345 | 0.0117 | 14 |  |  |  |  |  |  |
|  | F | *+/+* | 0.0298 | 0.0158 | 7 |  |  |  |  |  |  |
|  | F | *+/-* | 0.0525 | 0.0141 | 13 |  |  |  |  |  |  |
|  | F | *-/-* | 0.0167 | 0.0167 | 6 |  |  |  |  |  |  |

**Supplementary Table 12.** **Comparison of transition probability to different USVs**

Comparison transition probabilities to different types of USVs by sex and genotype. All p-values are shown in the table. Mann-Whitney *U* tests.
