# Supplementary material for: Sex-specific modulation of early life vocalization and cognition by Fmr1 gene dosage in a mouse model of Fragile X Syndrome
Source: Biol Sex Differ. 2024 Feb 21;15:18. doi: 10.1186/s13293-024-00594-3 (PMC10880250; doi:10.1186/s13293-024-00594-3)
Supplement: Supplementary file 13 — Supplementary Material 13: Supplementary table 13. Homing behavior in FXS mice. Comparison of total distance (cm), moving time (s), velocity (cm/s), latency to nest (s), time spent in the nest (s) and nest entries (#) among groups. All p-values are shown in the table, bold when p < 0.05. Mann-Whitney U tests [file 13293_2024_594_MOESM13_ESM.docx]

|  | **Sex** | ***Fmr1*** | **Median** | **Max** | **Min** | **N** | **p-value** | | | | | |
| --- | --- | --- | --- | --- | --- | --- | --- | --- | --- | --- | --- | --- |
|  |  |  |  |  |  |  | ***+/y*  VS  *-/y*** | ***+/y*  VS  *+/+*** | ***-/y*  VS  *-/-*** | ***+/+*  VS  *+/-*** | ***+/+*  VS  *-/-*** | ***+/-*  VS  *-/-*** |
| Tot distance (cm) | M | *+/y* | 189.1 | 490.2 | 82.43 | 10 | 0.4031 | **0.0420** | **0.0461** | **0.0415** | **0.0047** | 0.7108 |
|  | M | *-/y* | 154.7 | 321.5 | 74.93 | 14 |  |  |  |  |  |  |
|  | F | *+/+* | 123.4 | 161.4 | 76.77 | 6 |  |  |  |  |  |  |
|  | F | *+/-* | 318.4 | 541.3 | 68.93 | 12 |  |  |  |  |  |  |
|  | F | *-/-* | 265.4 | 468.9 | 125.70 | 7 |  |  |  |  |  |  |
| Moving (s) | M | *+/y* | 100.7 | 211.0 | 45.01 | 10 | 0.9771 | 0.1179 | 0.1285 | **0.0245** | **0.0350** | 0.7108 |
|  | M | *-/y* | 104.6 | 182.9 | 45.95 | 14 |  |  |  |  |  |  |
|  | F | *+/+* | 74.3 | 112.9 | 45.61 | 6 |  |  |  |  |  |  |
|  | F | *+/-* | 183.1 | 249.6 | 30.43 | 12 |  |  |  |  |  |  |
|  | F | *-/-* | 148.2 | 232.2 | 70.94 | 7 |  |  |  |  |  |  |
| Velocity (cm/s) | M | *+/y* | 0.63 | 1.63 | 0.27 | 10 | 0.4031 | **0.0420** | **0.0461** | **0.0415** | **0.0047** | 0.7108 |
|  | M | *-/y* | 0.52 | 1.07 | 0.25 | 14 |  |  |  |  |  |  |
|  | F | *+/+* | 0.41 | 0.54 | 0.26 | 6 |  |  |  |  |  |  |
|  | F | *+/-* | 1.06 | 1.80 | 0.23 | 12 |  |  |  |  |  |  |
|  | F | *-/-* | 0.88 | 1.56 | 0.42 | 7 |  |  |  |  |  |  |
| Latency (s) | M | *+/y* | 19.94 | 48.51 | 5.872 | 10 | **0.0001** | **0.0017** | **0.0023** | **0.0013** | **0.0047** | >0.9999 |
|  | M | *-/y* | 41.61 | 132.10 | 23.79 | 14 |  |  |  |  |  |  |
|  | F | *+/+* | 88.09 | 253.30 | 26.46 | 6 |  |  |  |  |  |  |
|  | F | *+/-* | 18.05 | 47.41 | 11.71 | 12 |  |  |  |  |  |  |
|  | F | *-/-* | 18.32 | 53.85 | 7.24 | 7 |  |  |  |  |  |  |
| Nest time (s) | M | *+/y* | 278.8 | 287.5 | 238.1 | 10 | **0.0005** | **0.0075** | **0.0016** | **0.0097** | **0.0140** | 0.6504 |
|  | M | *-/y* | 199.5 | 276.3 | 1.6 | 14 |  |  |  |  |  |  |
|  | F | *+/+* | 210.0 | 273.6 | 46.8 | 6 |  |  |  |  |  |  |
|  | F | *+/-* | 275.0 | 286.5 | 249.6 | 12 |  |  |  |  |  |  |
|  | F | *-/-* | 280.5 | 285.4 | 245.8 | 7 |  |  |  |  |  |  |
| Nest entries (#) | M | *+/y* | 2.5 | 5 | 1 | 10 | 0.6241 | 0.5393 | 0.9961 | 0.1469 | 0.3619 | 0.6327 |
|  | M | *-/y* | 2.5 | 11 | 1 | 14 |  |  |  |  |  |  |
|  | F | *+/+* | 1.0 | 5 | 1 | 6 |  |  |  |  |  |  |
|  | F | *+/-* | 4.0 | 9 | 1 | 12 |  |  |  |  |  |  |
|  | F | *-/-* | 2.0 | 10 | 1 | 7 |  |  |  |  |  |  |

**Supplementary Table 13. Homing behavior in FXS mice.** Comparison of total distance (cm), moving time (s), velocity (cm/s), latency to nest (s), time spent in the nest (s) and nest entries (#) among groups. All p-values are shown in the table, bold when p < 0.05. Mann-Whitney U tests.
