# Supplementary material for: Sex-specific modulation of early life vocalization and cognition by Fmr1 gene dosage in a mouse model of Fragile X Syndrome
Source: Biol Sex Differ. 2024 Feb 21;15:18. doi: 10.1186/s13293-024-00594-3 (PMC10880250; doi:10.1186/s13293-024-00594-3)
Supplement: Supplementary file 14 — Supplementary Material 14 [file 13293_2024_594_MOESM14_ESM.docx]

|  |  |  |  |  |  | **Two-way ANOVA** | | | **Holm-Šídák's  test** | | | |
| --- | --- | --- | --- | --- | --- | --- | --- | --- | --- | --- | --- | --- |
|  | **Sex** | ***Fmr1*** | **Mean** | **SEM** | **N** | **Sex** | ***Fmr1*** | **Interaction** | ***+/y*  VS  *-/y*** | ***+/y*  VS  *+/+*** | ***-/y*  VS  *-/-*** | ***+/+*  VS  *-/-*** |
|  |  |  |  |  |  | **p-value** | | | | | | |
| PND 10 | M | *+/y* | 5.159 | 0.166 | 22 | 0.4778 | 0.2848 | **0.0005** | **0.0142** | **0.0175** | **0.0138** | **0.0136** |
|  | M | *-/y* | 4.610 | 0.142 | 21 |  |  |  |  |  |  |  |
|  | F | *+/+* | 4.533 | 0.157 | 12 |  |  |  |  |  |  |  |
|  | F | *-/-* | 5.533 | 0.382 | 6 |  |  |  |  |  |  |  |
|  |  |  |  |  |  |  |  |  |  |  |  |  |
| PND 13 | M | *+/y* | 6.54 | 0.1875 | 10 | 0.2558 | 0.8461 | **0.0029** | **0.0147** | 0.1762 | **0.0050** | 0.0687 |
|  | M | *-/y* | 5.921 | 0.1476 | 14 |  |  |  |  |  |  |  |
|  | F | *+/+* | 6.167 | 0.1856 | 6 |  |  |  |  |  |  |  |
|  | F | *-/-* | 6.714 | 0.1438 | 7 |  |  |  |  |  |  |  |

**Table 1. Body weight in FXS pups at PND 10 and PND 13**

Comparison of body weight (g) across sex and genotype. All p-values are shown in the table, bold when p < 0.05. Two-way ANOVA followed by Holm-Šídák's post hoc test.

|  |  |  |  |  |  | **Two-way ANOVA** | | | **Holm-Šídák's  test** | | | |
| --- | --- | --- | --- | --- | --- | --- | --- | --- | --- | --- | --- | --- |
|  | **Sex** | ***Fmr1*** | **Mean** | **SEM** | **N** | **Sex** | ***Fmr1*** | **Interaction** | ***+/y*  VS  *-/y*** | ***+/y*  VS  *+/+*** | ***-/y*  VS  *-/-*** | ***+/+*  VS  *-/-*** |
|  |  |  |  |  |  | **p-value** | | | | | | |
| N. of USVs | M | *+/y* | 7.27 | 1.76 | 22 | **0.0044** | **0.0004** | 0.3926 | **0.0132** | 0.0915 | **0.0370** | **0.0132** |
|  | M | *-/y* | 22.71 | 4.75 | 21 |  |  |  |  |  |  |  |
|  | F | *+/+* | 18.33 | 6.39 | 12 |  |  |  |  |  |  |  |
|  | F | *-/-* | 42.83 | 8.33 | 6 |  |  |  |  |  |  |  |
| Latency (s) | M | *+/y* | 72.74 | 16.09 | 17 | 0.173 | 0.0972 | 0.5690 |  |  |  |  |
|  | M | *-/y* | 53.83 | 12.96 | 18 |  |  |  |  |  |  |  |
|  | F | *+/+* | 59.05 | 13.56 | 10 |  |  |  |  |  |  |  |
|  | F | *-/-* | 20.73 | 9.48 | 6 |  |  |  |  |  |  |  |

**Table 2. Vocalization propensity during early postnatal life of FXS mice.**

Comparison of number of emitted USVs and latency (s) across sex and genotype. All p-values are shown in the table, bold when p < 0.05. Two-way ANOVA followed by Holm-Šídák's post hoc test when necessary.

|  |  |  |  |  |  | **Two-way ANOVA** | | | **Holm-Šídák's  test** | | | |
| --- | --- | --- | --- | --- | --- | --- | --- | --- | --- | --- | --- | --- |
|  | **Sex** | ***Fmr1*** | **Mean** | **SEM** | **N** | **Sex** | ***Fmr1*** | **Interaction** | ***+/y*  VS  *-/y*** | ***+/y*  VS  *+/+*** | ***-/y*  VS  *-/-*** | ***+/+*  VS  *-/-*** |
|  |  |  |  |  |  | **p-value** | | | | | | |
| Length (s) | M | *+/y* | 0.021 | 0.001744 | 9 | 0.3763 | 0.5445 | **0.0239** | **0.0395** | 0.0586 | 0.2971 | 0.2757 |
|  | M | *-/y* | 0.0291 | 0.00272 | 14 |  |  |  |  |  |  |  |
|  | F | *+/+* | 0.0298 | 0.002034 | 7 |  |  |  |  |  |  |  |
|  | F | *-/-* | 0.0251 | 0.002355 | 6 |  |  |  |  |  |  |  |
| Pr. Freq.(kHz) | M | *+/y* | 59.41 | 1.288 | 9 | 0.1146 | 0.2141 | 0.8414 |  |  |  |  |
|  | M | *-/y* | 60.7 | 1.081 | 14 |  |  |  |  |  |  |  |
|  | F | *+/+* | 57.19 | 0.6609 | 7 |  |  |  |  |  |  |  |
|  | F | *-/-* | 58.98 | 1.073 | 6 |  |  |  |  |  |  |  |
| Power(dB/(Hz) | M | *+/y* | -72.41 | 0.6268 | 9 | 0.8252 | **0.0085** | 0.3910 | 0.1223 | 0.7005 | 0.7005 | 0.0536 |
|  | M | *-/y* | -74.29 | 0.8701 | 14 |  |  |  |  |  |  |  |
|  | F | *+/+* | -71.35 | 1.161 | 7 |  |  |  |  |  |  |  |
|  | F | *-/-* | -74.91 | 0.8499 | 6 |  |  |  |  |  |  |  |
| Δ freq. (kHz) | M | *+/y* | 5.553 | 0.6159 | 9 | **0.0422** | **0.0437** | 0.5932 | 0.0742 | 0.1422 | 0.2655 | 0.3327 |
|  | M | *-/y* | 7.161 | 0.4986 | 14 |  |  |  |  |  |  |  |
|  | F | *+/+* | 7.171 | 0.4477 | 7 |  |  |  |  |  |  |  |
|  | F | *-/-* | 8.122 | 0.7225 | 6 |  |  |  |  |  |  |  |

**Table 3. Core features of USVs in FXS mice.**

Comparison of length (s), principal frequency (kHz), power (dB/Hz) and Δ frequency (kHz) across sex and genotype. All p-values are shown in the table, bold when p < 0.05. Two-way ANOVA followed by Holm-Šídák's post hoc test when necessary.

| **A** |  |  |  |  |  |  |  |  |  |  |  |
| --- | --- | --- | --- | --- | --- | --- | --- | --- | --- | --- | --- |
| **One-way ANOVA** |  | **Holm-Šídák's test** | | | | | | | | | |
| **0.0022** |  | **1** | **2** | **3** | **4** | **5** | **6** | **7** | **8** | **9** | **10** |
|  | **1** |  | 0.4098 | >0.9999 | >0.9999 | >0.9999 | >0.9999 | 0.9065 | >0.9999 | 0.8674 | 0.9998 |
|  | **2** | 0.4098 |  | 0.0859 | 0.2784 | 0.8337 | 0.2939 | **0.0018** | 0.6031 | **0.0013** | **0.0367** |
|  | **3** | >0.9999 | 0.0859 |  | >0.9999 | 0.9981 | >0.9999 | 0.9982 | 0.9999 | 0.9976 | >0.9999 |
|  | **4** | >0.9999 | 0.2784 | >0.9999 |  | >0.9999 | >0.9999 | 0.9566 | >0.9999 | 0.9398 | >0.9999 |
|  | **5** | >0.9999 | 0.8337 | 0.9981 | >0.9999 |  | >0.9999 | 0.5241 | >0.9999 | 0.4571 | 0.9794 |
|  | **6** | >0.9999 | 0.2939 | >0.9999 | >0.9999 | >0.9999 |  | 0.9521 | >0.9999 | 0.9336 | >0.9999 |
|  | **7** | 0.9065 | **0.0018** | 0.9982 | 0.9566 | 0.5241 | 0.9521 |  | 0.7709 | >0.9999 | 0.9999 |
|  | **8** | >0.9999 | 0.6031 | 0.9999 | >0.9999 | >0.9999 | >0.9999 | 0.7709 |  | 0.7096 | 0.9982 |
|  | **9** | 0.8674 | **0.0013** | 0.9976 | 0.9398 | 0.4571 | 0.9336 | >0.9999 | 0.7096 |  | 0.9998 |
|  | **10** | 0.9998 | **0.0367** | >0.9999 | >0.9999 | 0.9794 | >0.9999 | 0.9999 | 0.9982 | 0.9998 |  |
|  |  |  |  |  |  |  |  |  |  |  |  |
| **B** |  |  |  |  |  |  |  |  |  |  |  |
| **One-way ANOVA** |  | **Holm-Šídák's test** | | | | | | | | | |
| **<0.0001** |  | **1** | **2** | **3** | **4** | **5** | **6** | **7** | **8** | **9** | **10** |
|  | **1** |  | **<0.0001** | 0.9945 | 0.9887 | 0.0542 | 0.0900 | 0.6469 | 0.9945 | **0.0187** | 0.7920 |
|  | **2** | **<0.0001** |  | **<0.0001** | **<0.0001** | **<0.0001** | **<0.0001** | **<0.0001** | **<0.0001** | **<0.0001** | **<0.0001** |
|  | **3** | 0.9945 | **<0.0001** |  | 0.9945 | **0.0109** | 0.3082 | 0.8971 | 0.9945 | 0.0855 | 0.9604 |
|  | **4** | 0.9887 | **<0.0001** | 0.9945 |  | **0.0043** | 0.5039 | 0.9604 | 0.9887 | 0.1610 | 0.9817 |
|  | **5** | 0.0542 | **<0.0001** | **0.0109** | **0.0043** |  | **<0.0001** | **<0.0001** | 0.0503 | **<0.0001** | **0.0001** |
|  | **6** | 0.0900 | **<0.0001** | 0.3082 | 0.5039 | **<0.0001** |  | 0.9817 | 0.0958 | 0.9945 | 0.9604 |
|  | **7** | 0.6469 | **<0.0001** | 0.8971 | 0.9604 | **<0.0001** | 0.9817 |  | 0.6582 | 0.8714 | 0.9945 |
|  | **8** | 0.9945 | **<0.0001** | 0.9945 | 0.9887 | 0.0503 | 0.0958 | 0.6582 |  | **0.0203** | 0.7971 |
|  | **9** | **0.0187** | **<0.0001** | 0.0855 | 0.1610 | **<0.0001** | 0.9945 | 0.8714 | **0.0203** |  | 0.7602 |
|  | **10** | 0.7920 | **<0.0001** | 0.9604 | 0.9817 | **0.0001** | 0.9604 | 0.9945 | 0.7971 | 0.7602 |  |

**Table 4. Vocal repertoire of *Fmr1 +/y and -/y* males at PND 10**

Comparison of percentage use among different types of USVs within the *+/y* **(A)** and *-/y* **(B)** male groups. All p-values are shown in the table, bold when p < 0.05. One-way ANOVA followed by Holm-Šídák's post hoc test when necessary. 1= Complex, 2=Downward Ramp, 3= Inverted-U, 4= Upward Ramp, 5= Complex Trill, 6= Short, 7= Step Down, 8= Flat, 9= Step Up, 10=Trill.

| **A** |  |  |  |  |  |  |  |  |  |  |  |
| --- | --- | --- | --- | --- | --- | --- | --- | --- | --- | --- | --- |
| **One-way ANOVA** |  | **Holm-Šídák's test** | | | | | | | | | |
| **<0.0001** |  | **1** | **2** | **3** | **4** | **5** | **6** | **7** | **8** | **9** | **10** |
|  | **1** |  | 0.2035 | 0.9997 | **0.0305** | 0.8253 | **0.0137** | **0.0337** | 0.9995 | **0.0070** | 0.0727 |
|  | **2** | 0.2035 |  | 0.4237 | **<0.0001** | **0.0022** | **<0.0001** | **<0.0001** | **0.0394** | **<0.0001** | **<0.0001** |
|  | **3** | 0.9997 | 0.4237 |  | **0.0098** | 0.5793 | **0.0041** | **0.0109** | 0.9907 | **0.0019** | **0.0263** |
|  | **4** | **0.0305** | **<0.0001** | **0.0098** |  | 0.7509 | 0.9997 | 0.9997 | 0.1691 | 0.9996 | 0.9997 |
|  | **5** | 0.8253 | **0.0022** | 0.5793 | 0.7509 |  | 0.5649 | 0.7630 | 0.9951 | 0.4065 | 0.9045 |
|  | **6** | **0.0137** | **<0.0001** | **0.0041** | 0.9997 | 0.5649 |  | 0.9997 | 0.085 | 0.9997 | 0.9996 |
|  | **7** | **0.0337** | **<0.0001** | **0.0109** | 0.9997 | 0.7630 | 0.9997 |  | 0.1814 | 0.9996 | 0.9997 |
|  | **8** | 0.9995 | **0.0394** | 0.9907 | 0.1691 | 0.9951 | 0.0850 | 0.1814 |  | **0.0466** | 0.3306 |
|  | **9** | **0.0070** | **<0.0001** | **0.0019** | 0.9996 | 0.4065 | 0.9997 | 0.9996 | **0.0466** |  | 0.9978 |
|  | **10** | 0.0727 | **<0.0001** | **0.0263** | 0.9997 | 0.9045 | 0.9996 | 0.9997 | 0.3306 | 0.9978 |  |
|  |  |  |  |  |  |  |  |  |  |  |  |
| **B** |  |  |  |  |  |  |  |  |  |  |  |
| **One-way ANOVA** |  | **Holm-Šídák's test** | | | | | | | | | |
| **<0.0001** |  | **1** | **2** | **3** | **4** | **5** | **6** | **7** | **8** | **9** | **10** |
|  | **1** |  | **0.0001** | 0.9402 | **<0.0001** | 0.9402 | **<0.0001** | **<0.0001** | 0.0974 | **<0.0001** | **<0.0001** |
|  | **2** | **0.0001** |  | **<0.0001** | **<0.0001** | **<0.0001** | **<0.0001** | **<0.0001** | **<0.0001** | **<0.0001** | **<0.0001** |
|  | **3** | 0.9402 | **<0.0001** |  | **0.0049** | 0.9997 | **0.0025** | **0.0023** | 0.8458 | **0.0002** | **0.0087** |
|  | **4** | **<0.0001** | **<0.0001** | **0.0049** |  | **0.0052** | 0.9997 | 0.9997 | 0.3541 | 0.9924 | 0.9997 |
|  | **5** | 0.9402 | **<0.0001** | 0.9997 | **0.0052** |  | **0.0027** | **0.0025** | 0.8458 | **0.0002** | **0.0092** |
|  | **6** | **<0.0001** | **<0.0001** | **0.0025** | 0.9997 | **0.0027** |  | 0.9997 | 0.2367 | 0.9983 | 0.9996 |
|  | **7** | **<0.0001** | **<0.0001** | **0.0023** | 0.9997 | **0.0025** | 0.9997 |  | 0.2275 | 0.9983 | 0.9996 |
|  | **8** | 0.0974 | **<0.0001** | 0.8458 | 0.3541 | 0.8458 | 0.2367 | 0.2275 |  | **0.0447** | 0.4771 |
|  | **9** | **<0.0001** | **<0.0001** | **0.0002** | 0.9924 | **0.0002** | 0.9983 | 0.9983 | **0.0447** |  | 0.9783 |
|  | **10** | **<0.0001** | **<0.0001** | **0.0087** | 0.9997 | **0.0092** | 0.9996 | 0.9996 | 0.4771 | 0.9783 |  |
|  |  |  |  |  |  |  |  |  |  |  |  |
| **C** |  |  |  |  |  |  |  |  |  |  |  |
| **One-way ANOVA** |  | **Holm-Šídák's test** | | | | | | | | | |
| **<0.0001** |  | **1** | **2** | **3** | **4** | **5** | **6** | **7** | **8** | **9** | **10** |
|  | **1** |  | **0.0016** | 0.9998 | 0.9976 | 0.9998 | 0.6405 | 0.9698 | 0.9653 | 0.6203 | 0.5685 |
|  | **2** | **0.0016** |  | **0.0073** | **<0.0001** | **0.0007** | **<0.0001** | **<0.0001** | **<0.0001** | **<0.0001** | **<0.0001** |
|  | **3** | 0.9998 | **0.0073** |  | 0.9676 | 0.9998 | 0.2902 | 0.7775 | 0.7546 | 0.2742 | 0.2384 |
|  | **4** | 0.9976 | **<0.0001** | 0.9676 |  | 0.9998 | 0.9951 | 0.9998 | 0.9998 | 0.9947 | 0.9936 |
|  | **5** | 0.9998 | **0.0007** | 0.9998 | 0.9998 |  | 0.7775 | 0.9936 | 0.9915 | 0.7734 | 0.7411 |
|  | **6** | 0.6405 | **<0.0001** | 0.2902 | 0.9951 | 0.7775 |  | 0.9998 | 0.9998 | >0.9999 | >0.9999 |
|  | **7** | 0.9698 | **<0.0001** | 0.7775 | 0.9998 | 0.9936 | 0.9998 |  | >0.9999 | 0.9998 | 0.9998 |
|  | **8** | 0.9653 | **<0.0001** | 0.7546 | 0.9998 | 0.9915 | 0.9998 | >0.9999 |  | 0.9998 | 0.9998 |
|  | **9** | 0.6203 | **<0.0001** | 0.2742 | 0.9947 | 0.7734 | >0.9999 | 0.9998 | 0.9998 |  | >0.9999 |
|  | **10** | 0.5685 | **<0.0001** | 0.2742 | 0.9936 | 0.7411 | >0.9999 | 0.9998 | 0.9998 | >0.9999 |  |

**Table 5. Vocal repertoire of *Fmr1* *+/+, +/- and -/-* females at PND 10**

Comparison of percentage use among different types of USVs within the *+/+* **(A)**, *+/-* **(B)** and *-/-* **(C)** female groups. All p-values are shown in the table, bold when p < 0.05. One-way ANOVA followed by Holm-Šídák's post hoc test when necessary. 1= Complex, 2=Downward Ramp, 3= Inverted-U, 4= Upward Ramp, 5= Complex Trill, 6= Short, 7= Step Down, 8= Flat, 9= Step Up, 10=Trill.

|  | **Sex** | ***Fmr1*** | **Mean** | **SEM** | **N** | **Two-way ANOVA** | | | **Holm-Šídák's  test** | | | |
| --- | --- | --- | --- | --- | --- | --- | --- | --- | --- | --- | --- | --- |
|  |  |  |  |  |  | **Sex** | ***Fmr1*** | **Interaction** | ***+/y*  VS  *-/y*** | ***+/y*  VS  *+/+*** | ***-/y*  VS  *-/-*** | ***+/+*  VS  *-/-*** |
|  |  |  |  |  |  | **p-value** | | | | | | |
| Complex | M | *+/y* | 11.255 | 3.752 | 9 | 0.1512 | 0.4825 | 0.7665 |  |  |  |  |
|  | M | *-/y* | 9.883 | 2.172 | 14 |  |  |  |  |  |  |  |
|  | F | *+/+* | 17.161 | 3.440 | 7 |  |  |  |  |  |  |  |
|  | F | *-/-* | 13.790 | 4.190 | 6 |  |  |  |  |  |  |  |
| Downw. R. | M | *+/y* | 25.007 | 6.392 | 9 | 0.4926 | 0.0909 | 0.9400 |  |  |  |  |
|  | M | *-/y* | 34.297 | 3.235 | 14 |  |  |  |  |  |  |  |
|  | F | *+/+* | 28.452 | 4.235 | 7 |  |  |  |  |  |  |  |
|  | F | *-/-* | 38.588 | 9.088 | 6 |  |  |  |  |  |  |  |
| Inverted-U | M | *+/y* | 7.743 | 3.210 | 9 | **0.0073** | 0.7965 | 0.6453 | 0.8781 | 0.0535 | 0.0944 | 0.8781 |
|  | M | *-/y* | 8.426 | 2.009 | 14 |  |  |  |  |  |  |  |
|  | F | *+/+* | 18.836 | 5.121 | 7 |  |  |  |  |  |  |  |
|  | F | *-/-* | 16.420 | 3.227 | 6 |  |  |  |  |  |  |  |
| Upward R. | M | *+/y* | 10.192 | 5.776 | 9 | 0.3217 | 0.6892 | 0.2812 |  |  |  |  |
|  | M | *-/y* | 7.676 | 2.045 | 14 |  |  |  |  |  |  |  |
|  | F | *+/+* | 2.552 | 1.053 | 7 |  |  |  |  |  |  |  |
|  | F | *-/-* | 8.001 | 2.738 | 6 |  |  |  |  |  |  |  |
| Complex Tr. | M | *+/y* | 14.320 | 4.380 | 9 | 0.1677 | 0.3749 | 0.7796 |  |  |  |  |
|  | M | *-/y* | 18.634 | 2.734 | 14 |  |  |  |  |  |  |  |
|  | F | *+/+* | 10.198 | 2.556 | 7 |  |  |  |  |  |  |  |
|  | F | *-/-* | 12.452 | 3.868 | 6 |  |  |  |  |  |  |  |
| Short | M | *+/y* | 10.368 | 3.193 | 9 | **0.0281** | **0.0386** | **0.0286** | **0.0018** | **0.0063** | 0.9955 | 0.9332 |
|  | M | *-/y* | 1.672 | 0.975 | 14 |  |  |  |  |  |  |  |
|  | F | *+/+* | 1.395 | 0.805 | 7 |  |  |  |  |  |  |  |
|  | F | *-/-* | 1.657 | 1.401 | 6 |  |  |  |  |  |  |  |
| Step Down | M | *+/y* | 1.393 | 1.035 | 9 | 0.5331 | 0.1506 | 0.9618 |  |  |  |  |
|  | M | *-/y* | 4.395 | 1.558 | 14 |  |  |  |  |  |  |  |
|  | F | *+/+* | 2.732 | 1.493 | 7 |  |  |  |  |  |  |  |
|  | F | *-/-* | 5.543 | 3.655 | 6 |  |  |  |  |  |  |  |
| Flat | M | *+/y* | 12.611 | 3.602 | 9 | 0.6092 | **0.0423** | 0.2747 | 0.4160 | 0.6815 | 0.4385 | 0.0981 |
|  | M | *-/y* | 9.785 | 1.265 | 14 |  |  |  |  |  |  |  |
|  | F | *+/+* | 14.287 | 4.359 | 7 |  |  |  |  |  |  |  |
|  | F | *-/-* | 5.202 | 1.128 | 6 |  |  |  |  |  |  |  |
| Step Up | M | *+/y* | 0.926 | 0.926 | 9 | 0.5439 | 0.8501 | 0.1710 |  |  |  |  |
|  | M | *-/y* | 0.130 | 0.130 | 14 |  |  |  |  |  |  |  |
|  | F | *+/+* | 0.408 | 0.408 | 7 |  |  |  |  |  |  |  |
|  | F | *-/-* | 1.455 | 1.167 | 6 |  |  |  |  |  |  |  |
| Trill | M | *+/y* | 6.186 | 2.245 | 9 | 0.1277 | 0.3237 | 0.6484 |  |  |  |  |
|  | M | *-/y* | 5.103 | 1.689 | 14 |  |  |  |  |  |  |  |
|  | F | *+/+* | 3.981 | 1.711 | 7 |  |  |  |  |  |  |  |
|  | F | *-/-* | 1.058 | 1.058 | 6 |  |  |  |  |  |  |  |

**Table 6. Vocal repertoire comparison**

Comparison of percentage use of different types of USVs across sex and genotype. All p-values are shown in the table, bold when p < 0.05. Two-way ANOVA followed by Holm-Šídák's post hoc test when necessary.

| **A** |  |  |  |  |  |  |  |  |  |  |  |
| --- | --- | --- | --- | --- | --- | --- | --- | --- | --- | --- | --- |
| **One-way ANOVA** |  | **Holm-Šídák's test** | | | | | | | | | |
| **0.0454** |  | **1** | **2** | **3** | **4** | **5** | **6** | **7** | **8** | **9** | **10** |
|  | **1** |  | 0.9993 | >0.9999 | 0.9979 | >0.9999 | >0.9999 | 0.9466 | >0.9999 | 0.9466 | 0.9969 |
|  | **2** | 0.9993 |  | 0.9823 | 0.6260 | 0.9957 | >0.9999 | 0.2305 | 0.9996 | 0.2305 | 0.5419 |
|  | **3** | >0.9999 | 0.9823 |  | >0.9999 | >0.9999 | 0.9969 | 0.9979 | >0.9999 | 0.9979 | >0.9999 |
|  | **4** | 0.9979 | 0.6260 | >0.9999 |  | 0.9998 | 0.8379 | >0.9999 | 0.9969 | >0.9999 | >0.9999 |
|  | **5** | >0.9999 | 0.9957 | >0.9999 | 0.9998 |  | 0.9988 | 0.9957 | >0.9999 | 0.9957 | 0.9996 |
|  | **6** | >0.9999 | >0.9999 | 0.9969 | 0.8379 | 0.9988 |  | 0.4153 | >0.9999 | 0.4153 | 0.7705 |
|  | **7** | 0.9466 | 0.2305 | 0.9979 | >0.9999 | 0.9957 | 0.4153 |  | 0.9273 | >0.9999 | >0.9999 |
|  | **8** | >0.9999 | 0.9996 | >0.9999 | 0.9969 | >0.9999 | >0.9999 | 0.9273 |  | 0.9273 | 0.9957 |
|  | **9** | 0.9466 | 0.2305 | 0.9979 | >0.9999 | 0.9957 | 0.4153 | >0.9999 | 0.9273 |  | >0.9999 |
|  | **10** | 0.9969 | 0.5419 | >0.9999 | >0.9999 | 0.9996 | 0.7705 | >0.9999 | 0.9957 | >0.9999 |  |
|  |  |  |  |  |  |  |  |  |  |  |  |
| **B** |  |  |  |  |  |  |  |  |  |  |  |
| **One-way ANOVA** |  | **Holm-Šídák's test** | | | | | | | | | |
| **<0.0001** |  | **1** | **2** | **3** | **4** | **5** | **6** | **7** | **8** | **9** | **10** |
|  | **1** |  | **<0.0001** | >0.9999 | >0.9999 | **0.0190** | 0.5332 | 0.9944 | 0.9996 | 0.5269 | >0.9999 |
|  | **2** | **<0.0001** |  | **<0.0001** | **<0.0001** | **0.0005** | **<0.0001** | **<0.0001** | **<0.0001** | **<0.0001** | **<0.0001** |
|  | **3** | >0.9999 | **<0.0001** |  | >0.9999 | **0.0268** | 0.4616 | 0.9881 | 0.9996 | 0.4541 | >0.9999 |
|  | **4** | >0.9999 | **<0.0001** | >0.9999 |  | **0.0048** | 0.8122 | 0.9996 | >0.9999 | 0.8109 | >0.9999 |
|  | **5** | **0.0190** | **0.0005** | **0.0268** | **0.0048** |  | **<0.0001** | **0.0003** | **0.0019** | **<0.0001** | **0.0062** |
|  | **6** | 0.5332 | **<0.0001** | 0.4616 | 0.8122 | **<0.0001** |  | 0.9944 | 0.9271 | >0.9999 | 0.7836 |
|  | **7** | 0.9944 | **<0.0001** | 0.9881 | 0.9996 | **0.0003** | 0.9944 |  | >0.9999 | 0.9944 | 0.9996 |
|  | **8** | 0.9996 | **<0.0001** | 0.9996 | >0.9999 | **0.0019** | 0.9271 | >0.9999 |  | 0.9271 | >0.9999 |
|  | **9** | 0.5269 | **<0.0001** | 0.4541 | 0.8109 | **<0.0001** | >0.9999 | 0.9944 | 0.9271 |  | 0.7805 |
|  | **10** | >0.9999 | **<0.0001** | >0.9999 | >0.9999 | **0.0062** | 0.7836 | 0.9996 | >0.9999 | 0.7805 |  |

**Table 7. Transition probability from different USVs in males at PND 10**

Comparison among transition probabilities from different types of USVs within the *+/y* **(A)** and *-/y* **(B)** male groups. All p-values are shown in the table, bold when p < 0.05. One-way ANOVA followed by Holm-Šídák's post hoc test when necessary. 1= Complex, 2=Downward Ramp, 3= Inverted-U, 4= Upward Ramp, 5= Complex Trill, 6= Short, 7= Step Down, 8= Flat, 9= Step Up, 10=Trill.

| **A** |  |  |  |  |  |  |  |  |  |  |  |
| --- | --- | --- | --- | --- | --- | --- | --- | --- | --- | --- | --- |
| **One-way ANOVA** |  | **Holm-Šídák's test** | | | | | | | | | |
| **0.0002** |  | **1** | **2** | **3** | **4** | **5** | **6** | **7** | **8** | **9** | **10** |
|  | **1** |  | 0.4355 | 0.4877 | 0.9984 | >0.9999 | 0.9978 | 0.9982 | 0.9978 | 0.9917 | >0.9999 |
|  | **2** | 0.4355 |  | >0.9999 | **0.0336** | 0.0750 | **0.0235** | **0.0288** | 0.9978 | **0.0146** | 0.1007 |
|  | **3** | 0.4877 | >0.9999 |  | **0.0420** | 0.0942 | **0.0297** | **0.0354** | 0.9982 | **0.0191** | 0.1251 |
|  | **4** | 0.9984 | **0.0336** | **0.0420** |  | >0.9999 | >0.9999 | >0.9999 | 0.5198 | >0.9999 | >0.9999 |
|  | **5** | >0.9999 | 0.0750 | 0.0942 | >0.9999 |  | >0.9999 | >0.9999 | 0.7587 | >0.9999 | >0.9999 |
|  | **6** | 0.9978 | **0.0235** | **0.0297** | >0.9999 | >0.9999 |  | >0.9999 | 0.4380 | >0.9999 | >0.9999 |
|  | **7** | 0.9982 | **0.0288** | **0.0354** | >0.9999 | >0.9999 | >0.9999 |  | 0.4877 | >0.9999 | >0.9999 |
|  | **8** | 0.9978 | 0.9978 | 0.9982 | 0.5198 | 0.7587 | 0.4380 | 0.4877 |  | 0.3315 | 0.8357 |
|  | **9** | 0.9917 | **0.0146** | **0.0191** | >0.9999 | >0.9999 | >0.9999 | >0.9999 | 0.3315 |  | >0.9999 |
|  | **10** | >0.9999 | 0.1007 | 0.1251 | >0.9999 | >0.9999 | >0.9999 | >0.9999 | 0.8357 | >0.9999 |  |
|  |  |  |  |  |  |  |  |  |  |  |  |
| **B** |  |  |  |  |  |  |  |  |  |  |  |
| **One-way ANOVA** |  | **Holm-Šídák's test** | | | | | | | | | |
| **<0.0001** |  | **1** | **2** | **3** | **4** | **5** | **6** | **7** | **8** | **9** | **10** |
|  | **1** |  | 0.9962 | 0.8030 | **0.0030** | 0.9969 | **0.0032** | **0.0016** | 0.0519 | **0.0003** | **0.0448** |
|  | **2** | 0.9962 |  | 0.1332 | **<0.0001** | 0.7155 | **<0.0001** | **<0.0001** | **0.0015** | **<0.0001** | **0.0013** |
|  | **3** | 0.8030 | 0.1332 |  | 0.4480 | 0.9969 | 0.4540 | 0.3426 | 0.9556 | 0.1310 | 0.9545 |
|  | **4** | **0.0030** | **<0.0001** | 0.4480 |  | 0.0533 | 0.9993 | 0.9993 | 0.9969 | 0.9969 | 0.9969 |
|  | **5** | 0.9969 | 0.7155 | 0.9969 | 0.0533 |  | 0.0556 | **0.0330** | 0.4126 | **0.0082** | 0.3799 |
|  | **6** | **0.0032** | **<0.0001** | 0.4540 | 0.9993 | 0.0556 |  | 0.9993 | 0.9969 | 0.9969 | 0.9969 |
|  | **7** | **0.0016** | **<0.0001** | 0.3426 | 0.9993 | **0.0330** | 0.9993 |  | 0.9962 | 0.9969 | 0.9962 |
|  | **8** | 0.0519 | **0.0015** | 0.9556 | 0.9969 | 0.4126 | 0.9969 | 0.9962 |  | 0.9545 | 0.9993 |
|  | **9** | **0.0003** | **<0.0001** | 0.1310 | 0.9969 | **0.0082** | 0.9969 | 0.9969 | 0.9545 |  | 0.9556 |
|  | **10** | **0.0448** | **0.0013** | 0.9545 | 0.9969 | 0.3799 | 0.9969 | 0.9962 | 0.9993 | 0.9556 |  |
|  |  |  |  |  |  |  |  |  |  |  |  |
| **C** |  |  |  |  |  |  |  |  |  |  |  |
| **One-way ANOVA** |  | **Holm-Šídák's test** | | | | | | | | | |
| **0.0005** |  | **1** | **2** | **3** | **4** | **5** | **6** | **7** | **8** | **9** | **10** |
|  | **1** |  | 0.9199 | 0.9981 | 0.9867 | >0.9999 | 0.3356 | 0.7969 | 0.3862 | 0.3658 | 0.4283 |
|  | **2** | 0.9199 |  | 0.2716 | 0.1077 | 0.4739 | **0.0025** | **0.0168** | **0.0033** | **0.0029** | **0.0039** |
|  | **3** | 0.9981 | 0.2716 |  | >0.9999 | >0.9999 | 0.9523 | 0.9981 | 0.9660 | 0.9615 | 0.9751 |
|  | **4** | 0.9867 | 0.1077 | >0.9999 |  | >0.9999 | 0.9934 | >0.9999 | 0.9953 | 0.9948 | 0.9968 |
|  | **5** | >0.9999 | 0.4739 | >0.9999 | >0.9999 |  | 0.8320 | 0.9878 | 0.8697 | 0.8561 | 0.8963 |
|  | **6** | 0.3356 | **0.0025** | 0.9523 | 0.9934 | 0.8320 |  | >0.9999 | >0.9999 | >0.9999 | >0.9999 |
|  | **7** | 0.7969 | **0.0168** | 0.9981 | >0.9999 | 0.9878 | >0.9999 |  | >0.9999 | >0.9999 | >0.9999 |
|  | **8** | 0.3862 | **0.0033** | 0.9660 | 0.9953 | 0.8697 | >0.9999 | >0.9999 |  | >0.9999 | >0.9999 |
|  | **9** | 0.3658 | **0.0029** | 0.9615 | 0.9948 | 0.8561 | >0.9999 | >0.9999 | >0.9999 |  | >0.9999 |
|  | **10** | 0.4283 | **0.0039** | 0.9751 | 0.9968 | 0.8963 | >0.9999 | >0.9999 | >0.9999 | >0.9999 |  |

**Table 8. Transition probability from different USVs in females at PND 10**

Comparison among the transition probabilities from different types of USVs within the *+/+* **(A)**, *+/-* **(B)** and *-/-* **(C)** female groups. All p-values are shown in the table, bold when p < 0.05. One-way ANOVA followed by Holm-Šídák's post hoc test when necessary. 1= Complex, 2=Downward Ramp, 3= Inverted-U, 4= Upward Ramp, 5= Complex Trill, 6= Short, 7= Step Down, 8= Flat, 9= Step Up, 10=Trill.

|  | **Sex** | ***Fmr1*** | **Mean** | **SEM** | **N** | **Two-way ANOVA** | | | **Holm-Šídák's  test** | | | |
| --- | --- | --- | --- | --- | --- | --- | --- | --- | --- | --- | --- | --- |
|  |  |  |  |  |  | **Sex** | ***Fmr1*** | **Interaction** | ***+/y*  VS  *-/y*** | ***+/y*  VS  *+/+*** | ***-/y*  VS  *-/-*** | ***+/+*  VS  *-/-*** |
|  |  |  |  |  |  | **p-value** | | | | | | |
| Complex | M | *+/y* | 0.0427 | 0.0181 | 9 | 0.1981 | 0.3376 | 0.1868 |  |  |  |  |
|  | M | *-/y* | 0.0361 | 0.0123 | 14 |  |  |  |  |  |  |  |
|  | F | *+/+* | 0.0421 | 0.0136 | 7 |  |  |  |  |  |  |  |
|  | F | *-/-* | 0.0827 | 0.0262 | 6 |  |  |  |  |  |  |  |
| Downw. R. | M | *+/y* | 0.0661 | 0.0182 | 9 | 0.7263 | **0.0277** | 0.1431 | **0.0075** | 0.3697 | 0.4153 | 0.6149 |
|  | M | *-/y* | 0.1615 | 0.0158 | 14 |  |  |  |  |  |  |  |
|  | F | *+/+* | 0.1125 | 0.0290 | 7 |  |  |  |  |  |  |  |
|  | F | *-/-* | 0.1327 | 0.0442 | 6 |  |  |  |  |  |  |  |
| Inverted-U | M | *+/y* | 0.0310 | 0.0138 | 9 | **0.0325** | 0.2547 | 0.1478 | 0.7923 | **0.0286** | 0.5923 | 0.1998 |
|  | M | *-/y* | 0.0379 | 0.0080 | 14 |  |  |  |  |  |  |  |
|  | F | *+/+* | 0.1099 | 0.0456 | 7 |  |  |  |  |  |  |  |
|  | F | *-/-* | 0.0538 | 0.0124 | 6 |  |  |  |  |  |  |  |
| Upward R. | M | *+/y* | 0.0156 | 0.0108 | 9 | 0.7239 | 0.0710 | 0.4780 |  |  |  |  |
|  | M | *-/y* | 0.0297 | 0.0092 | 14 |  |  |  |  |  |  |  |
|  | F | *+/+* | 0.0111 | 0.0054 | 7 |  |  |  |  |  |  |  |
|  | F | *-/-* | 0.0430 | 0.0218 | 6 |  |  |  |  |  |  |  |
| Complex Tr. | M | *+/y* | 0.0352 | 0.0169 | 9 | 0.3174 | **0.0260** | 0.7772 | 0.0767 | 0.6147 | 0.5862 | 0.2056 |
|  | M | *-/y* | 0.0909 | 0.0215 | 14 |  |  |  |  |  |  |  |
|  | F | *+/+* | 0.0197 | 0.0084 | 7 |  |  |  |  |  |  |  |
|  | F | *-/-* | 0.0632 | 0.0183 | 6 |  |  |  |  |  |  |  |
| Short | M | *+/y* | 0.0600 | 0.0209 | 9 | **0.0452** | **0.0158** | **0.0167** | **0.0004** | **0.0060** | 0.7530 | 0.9889 |
|  | M | *-/y* | 0.0018 | 0.0018 | 14 |  |  |  |  |  |  |  |
|  | F | *+/+* | 0.0071 | 0.0045 | 7 |  |  |  |  |  |  |  |
|  | F | *-/-* | 0.0069 | 0.0069 | 6 |  |  |  |  |  |  |  |
| Step Down | M | *+/y* | 0.0037 | 0.0037 | 9 | 0.5352 | 0.1095 | 0.9804 |  |  |  |  |
|  | M | *-/y* | 0.0185 | 0.0082 | 14 |  |  |  |  |  |  |  |
|  | F | *+/+* | 0.0092 | 0.0050 | 7 |  |  |  |  |  |  |  |
|  | F | *-/-* | 0.0245 | 0.0156 | 6 |  |  |  |  |  |  |  |
| Flat | M | *+/y* | 0.0444 | 0.0255 | 9 | 0.6548 | **0.0240** | 0.1872 | 0.4078 | 0.3910 | 0.5239 | 0.0503 |
|  | M | *-/y* | 0.0257 | 0.0044 | 14 |  |  |  |  |  |  |  |
|  | F | *+/+* | 0.0771 | 0.0288 | 7 |  |  |  |  |  |  |  |
|  | F | *-/-* | 0.0094 | 0.0057 | 6 |  |  |  |  |  |  |  |
| Step Up | M | *+/y* | 0.0037 | 0.0037 | 9 | 0.3811 | 0.5626 | 0.1999 |  |  |  |  |
|  | M | *-/y* | 0.0014 | 0.0014 | 14 |  |  |  |  |  |  |  |
|  | F | *+/+* | 0.0024 | 0.0024 | 7 |  |  |  |  |  |  |  |
|  | F | *-/-* | 0.0083 | 0.0057 | 6 |  |  |  |  |  |  |  |
| Trill | M | *+/y* | 0.0133 | 0.0073 | 9 | 0.6681 | 0.8043 | 0.2005 |  |  |  |  |
|  | M | *-/y* | 0.0310 | 0.0110 | 14 |  |  |  |  |  |  |  |
|  | F | *+/+* | 0.0232 | 0.0096 | 7 |  |  |  |  |  |  |  |
|  | F | *-/-* | 0.0113 | 0.0113 | 6 |  |  |  |  |  |  |  |

**Table 9.** **Comparison of transition probability from different USVs**

Comparison transition probabilities from different types of USVs across sex and genotype. All p-values are shown in the table, bold when p < 0.05. Two-way ANOVA followed by Holm-Šídák's post hoc test when necessary.

| **A** |  |  |  |  |  |  |  |  |  |  |  |
| --- | --- | --- | --- | --- | --- | --- | --- | --- | --- | --- | --- |
| **One-way ANOVA** |  | **Holm-Šídák's test** | | | | | | | | | |
| 0.5950 |  | **1** | **2** | **3** | **4** | **5** | **6** | **7** | **8** | **9** | **10** |
|  | **1** |  |  |  |  |  |  |  |  |  |  |
|  | **2** |  |  |  |  |  |  |  |  |  |  |
|  | **3** |  |  |  |  |  |  |  |  |  |  |
|  | **4** |  |  |  |  |  |  |  |  |  |  |
|  | **5** |  |  |  |  |  |  |  |  |  |  |
|  | **6** |  |  |  |  |  |  |  |  |  |  |
|  | **7** |  |  |  |  |  |  |  |  |  |  |
|  | **8** |  |  |  |  |  |  |  |  |  |  |
|  | **9** |  |  |  |  |  |  |  |  |  |  |
|  | **10** |  |  |  |  |  |  |  |  |  |  |
|  |  |  |  |  |  |  |  |  |  |  |  |
| **B** |  |  |  |  |  |  |  |  |  |  |  |
| **One-way ANOVA** |  | **Holm-Šídák's test** | | | | | | | | | |
| **<0.0001** |  | **1** | **2** | **3** | **4** | **5** | **6** | **7** | **8** | **9** | **10** |
|  | **1** |  | 0.9990 | 0.9990 | 0.9990 | 0.9985 | 0.0871 | 0.9691 | 0.9990 | **0.0010** | 0.8405 |
|  | **2** | 0.9990 |  | 0.9990 | 0.9980 | 0.9980 | 0.0522 | 0.9259 | 0.9990 | **0.0005** | 0.7187 |
|  | **3** | 0.9990 | 0.9990 |  | 0.9990 | 0.9990 | 0.3179 | 0.9980 | 0.9970 | **0.0067** | 0.9876 |
|  | **4** | 0.9990 | 0.9980 | 0.9990 |  | 0.9990 | 0.4896 | 0.9990 | 0.9876 | **0.0154** | 0.9970 |
|  | **5** | 0.9985 | 0.9980 | 0.9990 | 0.9990 |  | 0.5742 | 0.9990 | 0.9768 | **0.0216** | 0.9980 |
|  | **6** | 0.0871 | 0.0522 | 0.3179 | 0.4896 | 0.5742 |  | 0.9642 | **0.0129** | 0.9894 | 0.9940 |
|  | **7** | 0.9691 | 0.9259 | 0.9980 | 0.9990 | 0.9990 | 0.9642 |  | 0.6522 | 0.1598 | 0.9990 |
|  | **8** | 0.9990 | 0.9990 | 0.9970 | 0.9876 | 0.9768 | **0.0129** | 0.6522 |  | **<0.0001** | 0.3782 |
|  | **9** | **0.0010** | **0.0005** | **0.0067** | **0.0154** | **0.0216** | 0.9894 | 0.1598 | **<0.0001** |  | 0.3577 |
|  | **10** | 0.8405 | 0.7187 | 0.9876 | 0.9970 | 0.9980 | 0.9940 | 0.9990 | 0.3782 | 0.3577 |  |

**Table 10. Transition probability to different USVs in males at PND 10**

Comparison among transition probabilities to different types of USVs within the *+/y* **(A)** and *-/y* **(B)** male groups. All p-values are shown in the table, bold when p < 0.05. One-way ANOVA followed by Holm-Šídák's post hoc test when necessary. 1= Complex, 2=Downward Ramp, 3= Inverted-U, 4= Upward Ramp, 5= Complex Trill, 6= Short, 7= Step Down, 8= Flat, 9= Step Up, 10=Trill.

| **A** |  |  |  |  |  |  |  |  |  |  |  |
| --- | --- | --- | --- | --- | --- | --- | --- | --- | --- | --- | --- |
| **One-way ANOVA** |  | **Holm-Šídák's test** | | | | | | | | | |
| 0.1879 |  | **1** | **2** | **3** | **4** | **5** | **6** | **7** | **8** | **9** | **10** |
|  | **1** |  |  |  |  |  |  |  |  |  |  |
|  | **2** |  |  |  |  |  |  |  |  |  |  |
|  | **3** |  |  |  |  |  |  |  |  |  |  |
|  | **4** |  |  |  |  |  |  |  |  |  |  |
|  | **5** |  |  |  |  |  |  |  |  |  |  |
|  | **6** |  |  |  |  |  |  |  |  |  |  |
|  | **7** |  |  |  |  |  |  |  |  |  |  |
|  | **8** |  |  |  |  |  |  |  |  |  |  |
|  | **9** |  |  |  |  |  |  |  |  |  |  |
|  | **10** |  |  |  |  |  |  |  |  |  |  |
|  |  |  |  |  |  |  |  |  |  |  |  |
| **B** |  |  |  |  |  |  |  |  |  |  |  |
| **One-way ANOVA** |  | **Holm-Šídák's test** | | | | | | | | | |
| **0.0007** |  | **1** | **2** | **3** | **4** | **5** | **6** | **7** | **8** | **9** | **10** |
|  | **1** |  | 0.9998 | >0.9999 | >0.9999 | 0.9999 | 0.1368 | 0.5601 | 0.9997 | 0.1570 | >0.9999 |
|  | **2** | 0.9998 |  | >0.9999 | 0.9901 | 0.9901 | **0.0115** | 0.0997 | 0.9148 | **0.0138** | 0.9901 |
|  | **3** | >0.9999 | >0.9999 |  | 0.9998 | 0.9987 | 0.0548 | 0.3266 | 0.9932 | 0.0642 | 0.9998 |
|  | **4** | >0.9999 | 0.9901 | 0.9998 |  | >0.9999 | 0.4407 | 0.9025 | >0.9999 | 0.4730 | >0.9999 |
|  | **5** | 0.9999 | 0.9638 | 0.9987 | >0.9999 |  | 0.5831 | 0.9625 | >0.9999 | 0.6244 | >0.9999 |
|  | **6** | 0.1368 | **0.0115** | 0.0548 | 0.4407 | 0.5831 |  | 0.9999 | 0.7199 | >0.9999 | 0.4407 |
|  | **7** | 0.5601 | 0.0997 | 0.3266 | 0.9025 | 0.9625 | 0.9999 |  | 0.9870 | 0.9999 | 0.9025 |
|  | **8** | 0.9997 | 0.9148 | 0.9932 | >0.9999 | >0.9999 | 0.7199 | 0.9870 |  | 0.7562 | >0.9999 |
|  | **9** | 0.1570 | **0.0138** | 0.0642 | 0.4730 | 0.6244 | >0.9999 | 0.9999 | 0.7562 |  | 0.4730 |
|  | **10** | >0.9999 | 0.9901 | 0.9998 | >0.9999 | >0.9999 | 0.4407 | 0.9025 | >0.9999 | 0.4730 |  |
|  |  |  |  |  |  |  |  |  |  |  |  |
| **C** |  |  |  |  |  |  |  |  |  |  |  |
| **One-way ANOVA** |  | **Holm-Šídák's test** | | | | | | | | | |
| **0.0428** |  | **1** | **2** | **3** | **4** | **5** | **6** | **7** | **8** | **9** | **10** |
|  | **1** |  | >0.9999 | >0.9999 | >0.9999 | >0.9999 | 0.8990 | 0.9998 | 0.9998 | 0.9987 | 0.9692 |
|  | **2** | >0.9999 |  | >0.9999 | >0.9999 | >0.9999 | 0.4523 | 0.9692 | >0.9999 | 0.9014 | 0.6722 |
|  | **3** | >0.9999 | >0.9999 |  | >0.9999 | >0.9999 | 0.3792 | 0.9551 | >0.9999 | 0.8771 | 0.5844 |
|  | **4** | >0.9999 | >0.9999 | >0.9999 |  | >0.9999 | 0.8771 | 0.9998 | 0.9998 | 0.9975 | 0.9551 |
|  | **5** | >0.9999 | >0.9999 | >0.9999 | >0.9999 |  | 0.8990 | 0.9998 | 0.9998 | 0.9987 | 0.9692 |
|  | **6** | 0.8990 | 0.4523 | 0.3792 | 0.8771 | 0.899 |  | 0.9998 | 0.2160 | >0.9999 | >0.9999 |
|  | **7** | 0.9998 | 0.9692 | 0.9551 | 0.9998 | 0.9998 | 0.9998 |  | 0.8771 | >0.9999 | >0.9999 |
|  | **8** | 0.9998 | >0.9999 | >0.9999 | 0.9998 | 0.9998 | 0.2160 | 0.8771 |  | 0.6933 | 0.3793 |
|  | **9** | 0.9987 | 0.9014 | 0.8771 | 0.9975 | 0.9987 | >0.9999 | >0.9999 | 0.6933 |  | >0.9999 |
|  | **10** | 0.9692 | 0.6722 | 0.5844 | 0.9551 | 0.9692 | >0.9999 | >0.9999 | 0.3793 | >0.9999 |  |

**Table 11. Transition probability to different USVs in females at PND 10**

Comparison among the transition probabilities to different types of USVs within the *+/+* **(A)**, *+/-* **(B)** and *-/-* **(C)** female groups. All p-values are shown in the table, bold when p < 0.05. One-way ANOVA followed by Holm-Šídák's post hoc test when necessary. 1= Complex, 2=Downward Ramp, 3= Inverted-U, 4= Upward Ramp, 5= Complex Trill, 6= Short, 7= Step Down, 8= Flat, 9= Step Up, 10=Trill.

|  | **Sex** | ***Fmr1*** | **Mean** | **SEM** | **N** | **Two-way ANOVA** | | | **Holm-Šídák's  test** | | | |
| --- | --- | --- | --- | --- | --- | --- | --- | --- | --- | --- | --- | --- |
|  |  |  |  |  |  | **Sex** | ***Fmr1*** | **Interaction** | ***+/y*  VS  *-/y*** | ***+/y*  VS  *+/+*** | ***-/y*  VS  *-/-*** | ***+/+*  VS  *-/-*** |
|  |  |  |  |  |  | **p-value** | | | | | | |
| Complex | M | *+/y* | 0.0470 | 0.0146 | 9 | 0.8813 | 0.9466 | 0.4185 |  |  |  |  |
|  | M | *-/y* | 0.0602 | 0.0111 | 14 |  |  |  |  |  |  |  |
|  | F | *+/+* | 0.0614 | 0.0155 | 7 |  |  |  |  |  |  |  |
|  | F | *-/-* | 0.0503 | 0.0174 | 6 |  |  |  |  |  |  |  |
| Downw. R. | M | *+/y* | 0.0509 | 0.0095 | 9 | 0.6376 | 0.389 | 0.7112 |  |  |  |  |
|  | M | *-/y* | 0.0625 | 0.0064 | 14 |  |  |  |  |  |  |  |
|  | F | *+/+* | 0.0588 | 0.0138 | 7 |  |  |  |  |  |  |  |
|  | F | *-/-* | 0.0635 | 0.0051 | 6 |  |  |  |  |  |  |  |
| Inverted-U | M | *+/y* | 0.0333 | 0.0147 | 9 | 0.1922 | 0.3336 | 0.6624 |  |  |  |  |
|  | M | *-/y* | 0.0533 | 0.0109 | 14 |  |  |  |  |  |  |  |
|  | F | *+/+* | 0.0582 | 0.0158 | 7 |  |  |  |  |  |  |  |
|  | F | *-/-* | 0.0657 | 0.0103 | 6 |  |  |  |  |  |  |  |
| Upward R. | M | *+/y* | 0.0233 | 0.0125 | 9 | 0.35 | 0.3528 | 0.4171 |  |  |  |  |
|  | M | *-/y* | 0.0499 | 0.0114 | 14 |  |  |  |  |  |  |  |
|  | F | *+/+* | 0.0500 | 0.0189 | 7 |  |  |  |  |  |  |  |
|  | F | *-/-* | 0.0518 | 0.0177 | 6 |  |  |  |  |  |  |  |
| Complex Tr. | M | *+/y* | 0.0302 | 0.0128 | 9 | 0.3288 | 0.5099 | 0.4036 |  |  |  |  |
|  | M | *-/y* | 0.0485 | 0.0074 | 14 |  |  |  |  |  |  |  |
|  | F | *+/+* | 0.0524 | 0.0164 | 7 |  |  |  |  |  |  |  |
|  | F | *-/-* | 0.0502 | 0.0123 | 6 |  |  |  |  |  |  |  |
| Short | M | *+/y* | 0.0311 | 0.0157 | 9 | 0.9406 | 0.1942 | 0.6936 |  |  |  |  |
|  | M | *-/y* | 0.0179 | 0.0100 | 14 |  |  |  |  |  |  |  |
|  | F | *+/+* | 0.0357 | 0.0180 | 7 |  |  |  |  |  |  |  |
|  | F | *-/-* | 0.0111 | 0.0111 | 6 |  |  |  |  |  |  |  |
| Step Down | M | *+/y* | 0.0222 | 0.0147 | 9 | 0.9353 | 0.5542 | 0.6313 |  |  |  |  |
|  | M | *-/y* | 0.0393 | 0.0121 | 14 |  |  |  |  |  |  |  |
|  | F | *+/+* | 0.0286 | 0.0184 | 7 |  |  |  |  |  |  |  |
|  | F | *-/-* | 0.0304 | 0.0167 | 6 |  |  |  |  |  |  |  |
| Flat | M | *+/y* | 0.0422 | 0.0158 | 9 | 0.9965 | 0.0504 | 0.8543 |  |  |  |  |
|  | M | *-/y* | 0.0685 | 0.0095 | 14 |  |  |  |  |  |  |  |
|  | F | *+/+* | 0.0395 | 0.0160 | 7 |  |  |  |  |  |  |  |
|  | F | *-/-* | 0.0711 | 0.0160 | 6 |  |  |  |  |  |  |  |
| Step Up | M | *+/y* | 0.0111 | 0.0111 | 9 | 0.4053 | 0.4053 | **0.0357** | 0.2763 | 0.3547 | 0.0718 | 0.125 |
|  | M | *-/y* | 0.0000 | 0.0000 | 14 |  |  |  |  |  |  |  |
|  | F | *+/+* | 0.0000 | 0.0000 | 7 |  |  |  |  |  |  |  |
|  | F | *-/-* | 0.0250 | 0.0171 | 6 |  |  |  |  |  |  |  |
| Trill | M | *+/y* | 0.0241 | 0.0128 | 9 | 0.6792 | 0.9283 | 0.4254 |  |  |  |  |
|  | M | *-/y* | 0.0345 | 0.0117 | 14 |  |  |  |  |  |  |  |
|  | F | *+/+* | 0.0298 | 0.0158 | 7 |  |  |  |  |  |  |  |
|  | F | *-/-* | 0.0167 | 0.0167 | 6 |  |  |  |  |  |  |  |

**Table 12.** **Comparison of transition probability to different USVs**

Comparison transition probabilities to different types of USVs across sex and genotype. All p-values are shown in the table. Two-way ANOVA followed by Holm-Šídák's post hoc test when necessary.

|  |  |  |  |  |  | **Two-way ANOVA** | | | **Holm-Šídák's  test** | | | |
| --- | --- | --- | --- | --- | --- | --- | --- | --- | --- | --- | --- | --- |
|  | **Sex** | ***Fmr1*** | **Mean** | **SEM** | **N** | **Sex** | ***Fmr1*** | **Interaction** | ***+/y*  VS  *-/y*** | ***+/y*  VS  *+/+*** | ***-/y*  VS  *-/-*** | ***+/+*  VS  *-/-*** |
|  |  |  |  |  |  | **p-value** | | | | | | |
| Tot distance (cm) | M | *+/y* | 215.0 | 37.70 | 10 | 0.9581 | 0.1142 | **0.0030** | 0.1952 | **0.0421** | **0.0402** | **0.0100** |
|  | M | *-/y* | 165.3 | 17.19 | 14 |  |  |  |  |  |  |  |
|  | F | *+/+* | 115.9 | 12.73 | 6 |  |  |  |  |  |  |  |
|  | F | *-/-* | 267.7 | 45.06 | 7 |  |  |  |  |  |  |  |
| Moving (s) | M | *+/y* | 110.5 | 15.38 | 10 | 0.8560 | **0.0316** | **0.0129** | 0.7498 | 0.1101 | 0.0840 | **0.0093** |
|  | M | *-/y* | 104.6 | 10.51 | 14 |  |  |  |  |  |  |  |
|  | F | *+/+* | 72.9 | 10.11 | 6 |  |  |  |  |  |  |  |
|  | F | *-/-* | 147.7 | 21.94 | 7 |  |  |  |  |  |  |  |
| Velocity (cm/s) | M | *+/y* | 0.72 | 0.1256 | 10 | 0.9513 | 0.1128 | **0.0030** | 0.1957 | **0.0424** | **0.0393** | **0.0098** |
|  | M | *-/y* | 0.55 | 0.0573 | 14 |  |  |  |  |  |  |  |
|  | F | *+/+* | 0.39 | 0.0424 | 6 |  |  |  |  |  |  |  |
|  | F | *-/-* | 0.89 | 0.1508 | 7 |  |  |  |  |  |  |  |
| Latency (s) | M | *+/y* | 20.81 | 3.61 | 10 | **0.0328** | **0.0354** | **0.0002** | 0.0922 | **0.0002** | 0.1367 | **0.0006** |
|  | M | *-/y* | 52.03 | 8.15 | 14 |  |  |  |  |  |  |  |
|  | F | *+/+* | 118.70 | 39.90 | 6 |  |  |  |  |  |  |  |
|  | F | *-/-* | 21.33 | 5.66 | 7 |  |  |  |  |  |  |  |
| Nest time (s) | M | *+/y* | 272.7 | 5.63 | 10 | 0.8399 | 0.8491 | **0.0007** | **0.0048** | **0.0236** | **0.0115** | **0.0342** |
|  | M | *-/y* | 169.9 | 27.32 | 14 |  |  |  |  |  |  |  |
|  | F | *+/+* | 180.2 | 39.99 | 6 |  |  |  |  |  |  |  |
|  | F | *-/-* | 273.0 | 5.66 | 7 |  |  |  |  |  |  |  |
| Nest entries (#) | M | *+/y* | 2.40 | 0.40 | 10 | 0.6859 | 0.1562 | 0.8649 |  |  |  |  |
|  | M | *-/y* | 4.00 | 0.98 | 14 |  |  |  |  |  |  |  |
|  | F | *+/+* | 2.17 | 0.75 | 6 |  |  |  |  |  |  |  |
|  | F | *-/-* | 3.43 | 1.21 | 7 |  |  |  |  |  |  |  |

**Table 13. Homing behavior in FXS mice.**

Comparison of total distance (cm), moving time (s), velocity (cm/s), latency to nest (s), time spent in the nest (s) and nest entries (#) among groups. All p-values are shown in the table, bold when p < 0.05. Two-way ANOVA followed by Holm-Šídák's post hoc test when necessary.
