# Supplementary figures and images for: Sex-specific modulation of early life vocalization and cognition by Fmr1 gene dosage in a mouse model of Fragile X Syndrome
Source: Biol Sex Differ. 2024 Feb 21;15:18. doi: 10.1186/s13293-024-00594-3 (PMC10880250; doi:10.1186/s13293-024-00594-3)

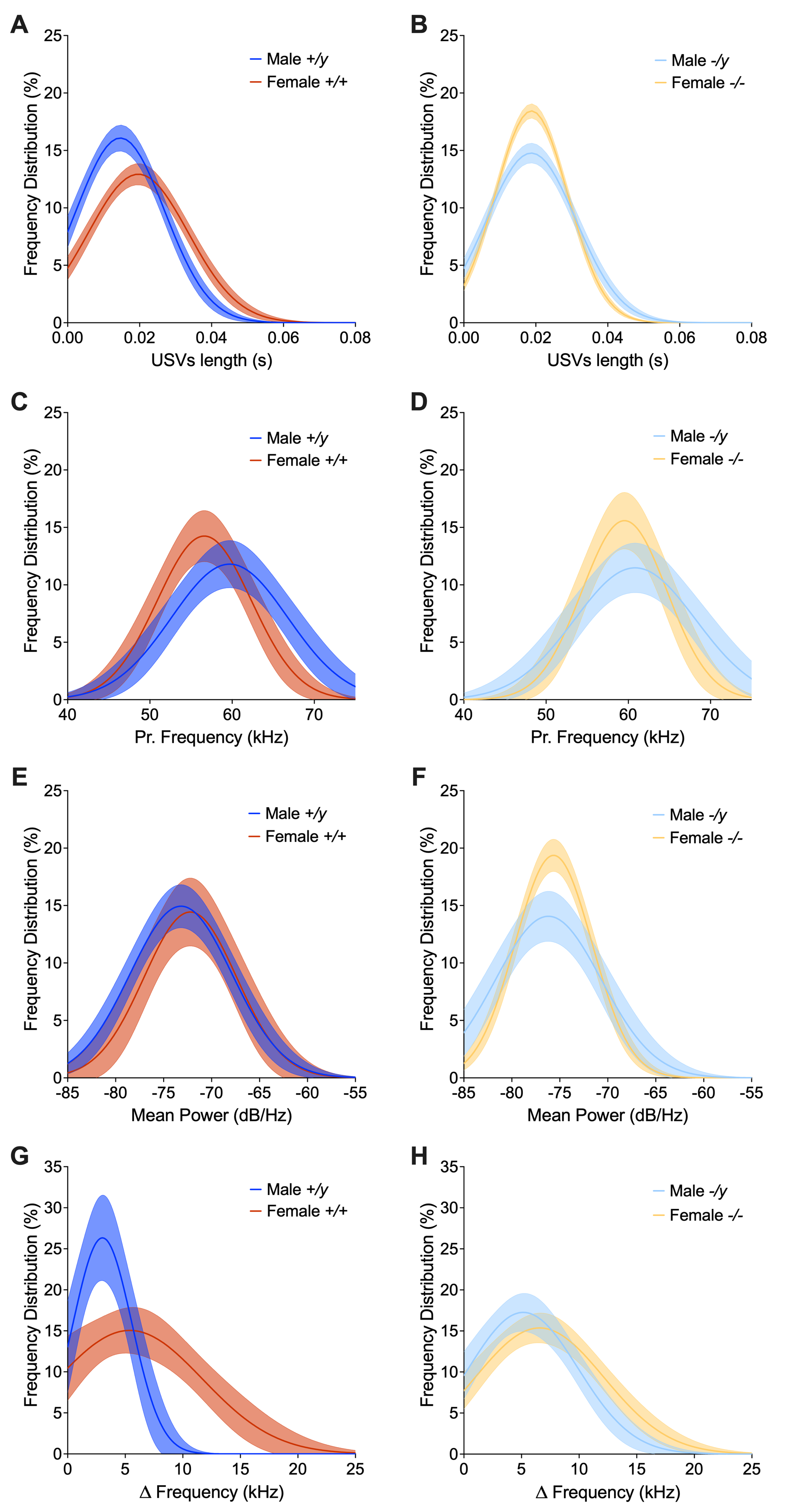

Supplement: Supplementary file 15 — Supplementary Material 15 [file 13293_2024_594_MOESM15_ESM.tiff]

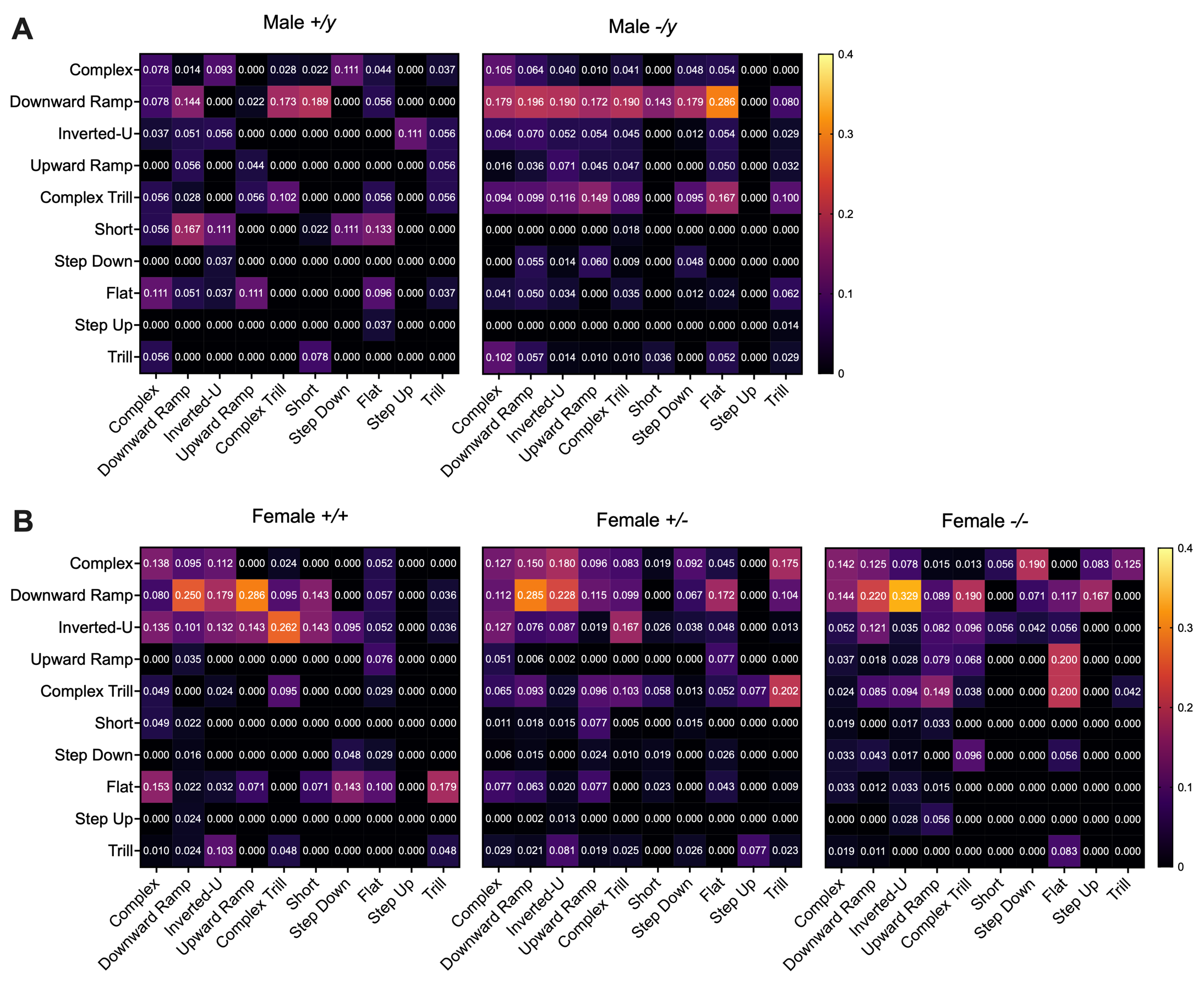

Supplement: Supplementary file 16 — Supplementary Material 16 [file 13293_2024_594_MOESM16_ESM.tiff]
